# Solid-Phase Synthesis of ɤ-Lactone and 1,2-Oxazine Derivatives and Their Efficient Chiral Analysis

**DOI:** 10.1371/journal.pone.0166558

**Published:** 2016-11-28

**Authors:** Sona Krupkova, Gonzalo Pazos Aguete, Leona Kocmanova, Tereza Volna, Martin Grepl, Lucie Novakova, Marvin John Miller, Jan Hlavac

**Affiliations:** 1 Institute of Molecular and Translation Medicine, Faculty of Medicine and Dentistry, Palacky University, Olomouc, Czech Republic; 2 Department of Organic Chemistry, Faculty of Science, Palacky University, Olomouc, Czech Republic; 3 Department of Analytical Chemistry, Faculty of Pharmacy in Hradec Králové, Charles University in Prague, Prague, Czech Republic; 4 Department of Chemistry and Biochemistry, University of Notre Dame, IN, United States of America; University of East Anglia, UNITED KINGDOM

## Abstract

Derivatives of 3-methyl-3,6-dihydro-2*H*-1,2-oxazine-6-carboxylic acid prepared by regioselective hetero Diels-Alder reaction of arylnitroso compounds with sorbic acid were used for solid-phase synthesis of a library of derivatives that included modification of carboxylic group, dihydroxylation of double bond and cleavage of N-O bond. Derivatives of 2,3,4-trihydroxyhexanoic acid obtained from 3,6-dihydro-2*H*-1,2-oxazines after double bond dihydroxylation and N-O cleavage were used for simple and stereoselective formation of chiral lactones derived from 3,4-dihydroxydihydrofuran-2(3*H*)-one. The final compounds obtained as a mixture of stereoisomers were analyzed with use of chiral HPLC and SFC. HPLC analyses were not successful for all derivatives or required lengthy chromatography. On the other hand SFC afforded much shorter analyses and was effective for all studied derivatives. The method of synthesis and analysis is thus suitable for future study of stereoselective synthesis of lactones and other derivatives from single oxazine derivatives and application of high-throughput synthesis on solid-support and combinatorial chemistry.

## Introduction

3,6-Dihydro-1,2-oxazine belongs to very interesting scaffold, which is compatible with a wide variety of transformations. These cycloadducts have become valuable key intermediates for the synthesis of biologically interesting molecules especially because they offer plethora of modification possibilities including N-O bond cleavage mediated by titanocene(III) chloride,[1] Mo(CO)_6_[2;3] or other reducing agents providing ready access to 1,4-amino alcohols.[4;5] Before or after N-O reduction, the double bond can be hydroborated or oxidized to give epoxides, diols or even diacids after complete cleavage. Epoxidation, hydroboration[6] or hydroxylation[7] has been used for the synthesis of aminosugars. Alternatively, the alkene has been used in a domino metathesis reaction to give isoxazolopyridines.[8] Additional modifications of 3,6-dihydro-1,2-oxazines have been reviewed and include applications for the syntheses of various biologically important compounds including carbocyclic nucleosides, azasugars, tropane alkaloids and other natural compounds.[9] Thus, although chemical transformations of oxazines have been extensively explored in solution, the only reported solid phase based oxazine conversions were described by Krchnak et al, who described acid-mediated cleavage of oxazine C-O bond.[10;11] Apart from wide variety of interesting products, 3,6-dihydro-1,2-oxazine derivatives were also used for the preparation of lactones via quite complex synthesis.[12;13] The lactone moiety occurs in wide range of natural biologically active compounds including compounds such as vitamin C,[14] kavain having anxiolytic and sedative effects,[15] helenalin,[16] the anti- inflammatory active component of Arnica or parthenolide[17;18] with anti-inflammatory, anticancer and antiviral properties, contained in feverfew (*Pyrethrum parthenium*).

Due to their biological properties, synthetic methods compatible with preparation of structurally varied lactones are of considerable interest. Many synthetic approaches to lactones have been reported[19–23] but only a few were developed for solid-phase chemistry[24–26] that could additionally facilitate the extensions of related molecular diversity via application of combinatorial chemistry in high throughput mode. In addition none of these solid-phase methods employs the oxazine core as the lactone precursor. Here we report the solid-phase syntheses of 3,6-dihydro-1,2-oxazines derived from hetero Diels-Alder (HDA) reactions of sorbic acid and arylnitroso derivatives and their further transformation to 3,6-dihydro-1,2-oxazines and their 4,5-dihydroxyanalogues, as well as stereoselective conversion to 3,4-dihydroxy-ɤ-lactones. Development of chiral separation of enantiomeric products with use of chiral HPLC and SFC, that would facilitate development of stereoselective synthetic routes in future, is also described.

## Results and Discussion

### Synthesis

**Fig 1:**
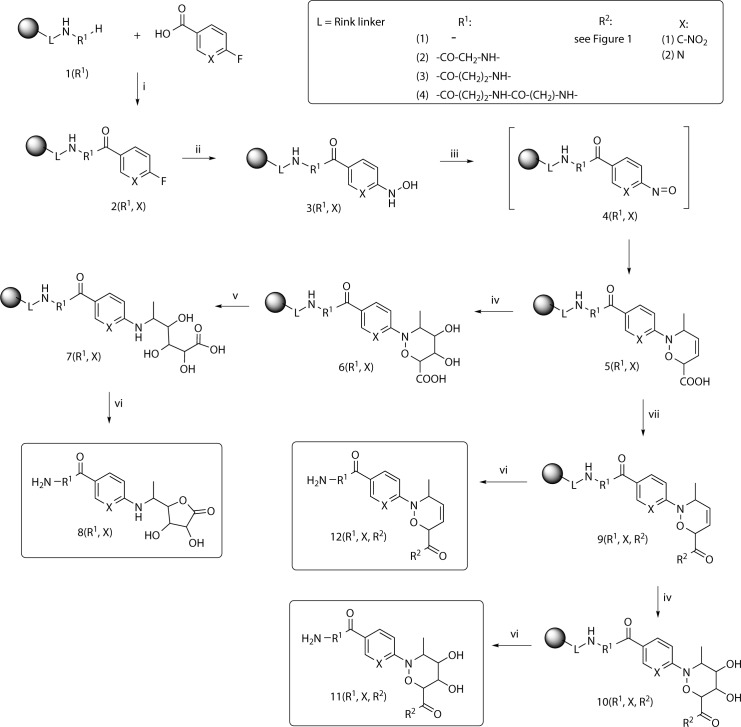
The synthetic pathway for preparation of 4,5-dihydroxy-3,6-dihydro-1,2-oxazines 11(R^1^,X,R^2^) and dihydroxy ɤ-lactones 8(R^1^,X). Reagents and conditions: i) HOBt, DIC, DMF/DCM, rt, o.n.; ii) NH_2_OH·HCl, pyridine, 75°C, o.n. (for X = C-NO_2_), DMSO, DBU, 80°C, 20 h (for X = N); iii) TBAPI, sorbic acid, DCM, rt, 3 h; iv) OsO_4_, NMMO, DCM, rt, o.n.; v) Mo(CO)_6_, H_2_O, DMF, 100°C, 1 h; vi) TFA/DCM (1:1), rt, 1 hr; vii) amine, DMAP, HOBt, DIC, DMF/DCM, rt.

The synthesis of the final ɤ-lactones **8(R**^**1**^**,X)** as well as oxazines **11(R**^**1**^**,X,R**^**2**^**)** and **12(R**^**1**^**,X, R**^**2**^**)** is depicted in Fig 1.

The synthesis of the starting oxazine system **5(R**^**1**^**,X)** was adopted from that described previously.[27] However, the products were diversified by incorporation of Rink resin-bound amino acid or dipeptide (Fig 1). The amino group of the Rink linker or immobilized amino acids **1(R**^**1**^**)** was acylated by fluoroaromatic carboxylic acids using traditional HOBt active esters to give derivative **2(R**^**1**^**,X)**. The use of two different acids (6-fluoronicotinic acid or 4-fluoro-3-nitrobenzoic acid) provided the second element of diversity. Nucleophilic aromatic substitution of fluorine afforded hydroxylamine derivatives **3(R**^**1**^**,X)** ready for subsequent oxidation to the intermediate nitroso compounds **4(R**^**1**^**,X)**. The nitroso dieonophiles were generated *in situ* by tetrabutyl ammonium(meta)periodate (TBAPI) oxidation and reacted with sorbic acid to give related 3,6-dihydro-1,2-oxazines **5(R**^**1**^**,X)**. Typically, the reaction was complete in 3 h. Although use of the unsymmetrical diene could give two regioisomeric cycloadducts, in this case only one isomer was detected, consistent with the literature precedents,[27;28] in which the authors reported the preferential formation of the regioisomer with bulkier substituents on the C6 carbon. The treatment of compounds **5(R**^**1**^**,X)** with OsO_4_ yielded the dihydroxyoxazines **6(R**^**1**^**,X)**. Following cleavage of N-O bond led to the trihydroxy derivatives **7(R**^**1**^**,X)**. The reaction was first performed with Mo(CO)_6_ in wet acetonitrile (MeCN) under the overnight reflux similarly to conditions described for reaction in solution.[2;3] The use of dimethylformamide (DMF) instead of MeCN enabled increasing of the reaction temperature and thus the full conversion was reached in one hour.

**Fig 2:**
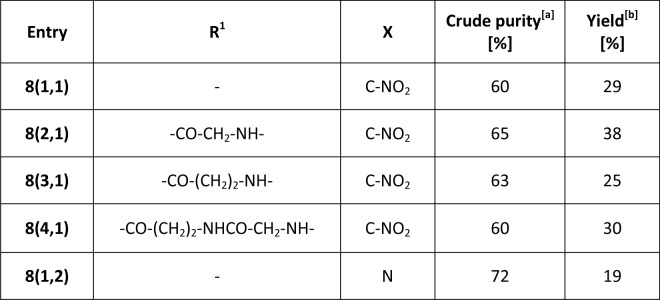
Summary of the final lactones 8(R^1^,X). [a] Purity was determined from LC traces with use of PDA detector; [b] Overall yield after the HPLC purification.

The final step, the formation of the lactone ring, was performed by cyclization of trihydroxy derivatives **7(R**^**1**^**,X)** in acidic cleavage cocktail during its releasing from the resin. The final lactones **8(R**^**1**^**,X)** were obtained after one-hour treatment of the resins **7(R**^**1**^**,X)** with 50% trifluoroacetic acid (TFA) in dichlormethane (DCM) at room temperature in acceptable yield (Fig 2).

**Fig 3:**
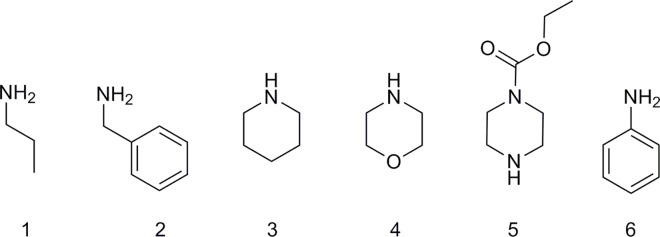
Amines used for synthesis of carboxamides 9(R^1^,X, R^2^).

Compounds **5(R**^**1**^**,X)** were subsequently converted to the corresponding amides **9(R**^**1**^**,X,R**^**2**^**)** by coupling with amines listed in Fig 3.

**Fig 4:**
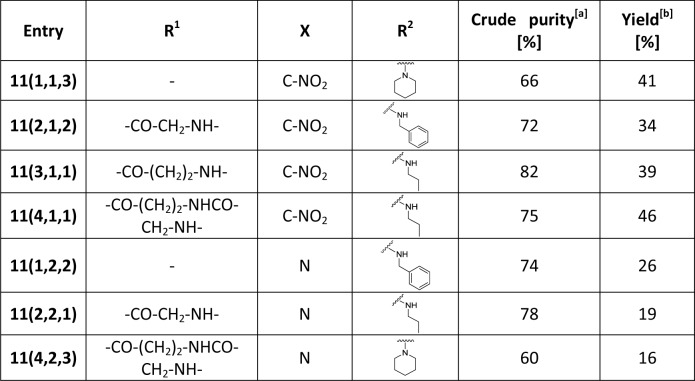
Summary of the final oxazines 11(R^1^,X,R^2^). [a] Purity was determined from LC traces with use of PDA detector; [b] Overall yield after the HPLC purification.

Further transformation consisted in dihydroxylation of the oxazine double bond. The smooth reaction with osmium tetroxide (OsO_4_) and *N*-methyl morpholine oxide (NMMO) as an oxidative agent in DCM afforded dihydroxy derivatives **10(R**^**1**^**,X,R**^**2**^**)**, which were subsequently cleaved from the resin to afford final dihydroxyoxazines **11(R**^**1**^**,X,R**^**2**^**)** in acceptable yields (see Fig 4).

**Fig 5:**
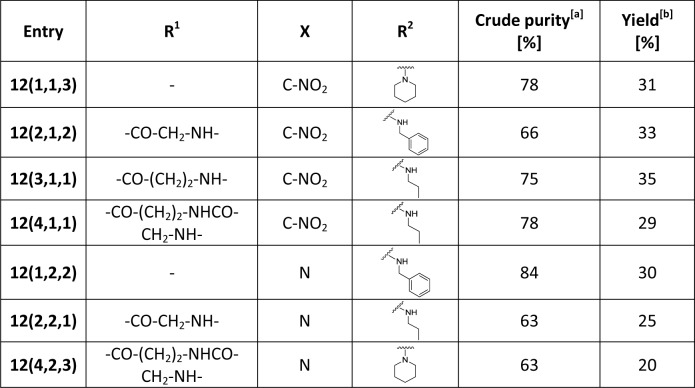
Summary of the final oxazines 12(R^1^,X,R^2^). [a] Purity was determined from LC traces with use of PDA detector; [b] Overall yield after the HPLC purification.

The other final compounds **12(R**^**1**^**,X,R**^**2**^**)** were obtained by cleavage of derivatives **9(R**^**1**^**,X,R**^**2**^**)** from the resin (see Fig 5).

The study was focused on the synthesis development with not full combination of used building blocks. The summary of the prepared compounds is showed in Figs 2, 4 and 5.

### Stereochemistry

**Fig 6:**
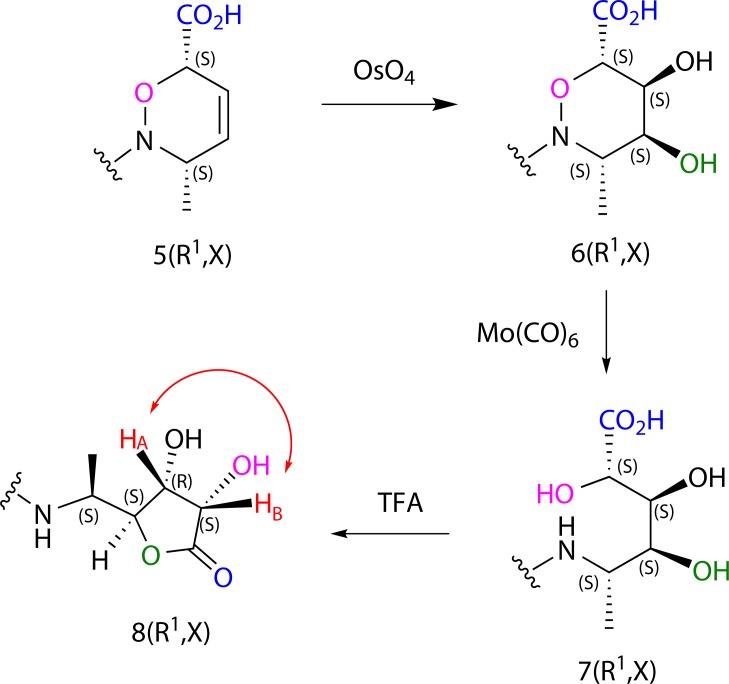
Stereoselective formation of lactone 8(R^1^,X) demonstrated on one enantiomer of oxazine 5(R^1^,X).

We supposed that the steric hindrance of carboxylic and methyl group in oxazine **5(R**^**1**^**,X)** directed the dihydroxylation with osmium tetraoxide to the opposite part of the molecule to afford dihydroxyderivative **6(R**^**1**^**,X)** (and subsequently **7(R**^**1**^**,X)**) with high stereoselectivity. Taking this fact into account subsequent cyclization of carboxylic group and γ-hydroxygroup of derivative **7(R**^**1**^**,X)** can afford the final lactone **8(R**^**1**^**,X)** in major form having the two hydroxyl groups in *cis*-configuration (Fig 6).

**Fig 7:**
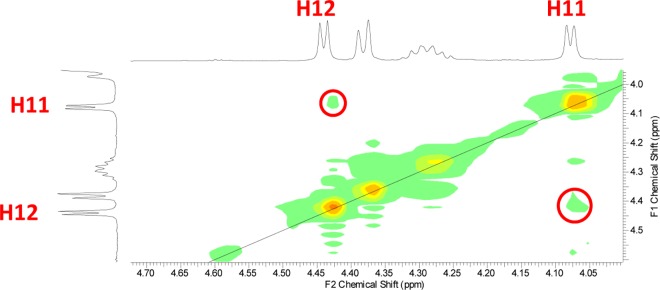
The ROESY experiment for derivative 8(1,1) with spatial interaction between H_A_ and H_B_.

Because the oxazine **5(R**^**1**^**,X)** is formed as the racemic mixture, we should get mainly two enantiomers of dihydroxyderivative **6(R**^**1**^**,X)** and finally two enantiomers of lactone **8(R**^**1**^**,X)**. Due to this fact it is finally possible to expect the formation of lactones predominantly in mixture of only two enantiomers, where stereoisomery comes from HDA reaction itself. Stereoselectivity of the final lactones is thus directed only in step of HDA reaction. To confirm the stereoselectivity of lactone **8(R**^**1**^**,X)** formation, we did the ROESY NMR experiment for derivative **8(1,1)** which showed the spatial interaction between H_A_ and H_B_ protons (Fig 7), giving the evidence of *cis*-configuration.

### Chiral separation

For the checking of the optical purity as well as a stereoselective control of the reaction it is necessary to develop suitable analytical methods, which must be i) able to determine the optical purity of final products after isolation ii) able to determine optical purity of compound of interest in crude reaction mixtures to give response for modification of the conditions for possible stereoselective synthesis iii) fast enough to reflect synthetic quickness and high-throughput approach and thus enable rapid optimization of reaction conditions as well as rapid control of optical purity.

**Fig 8:**
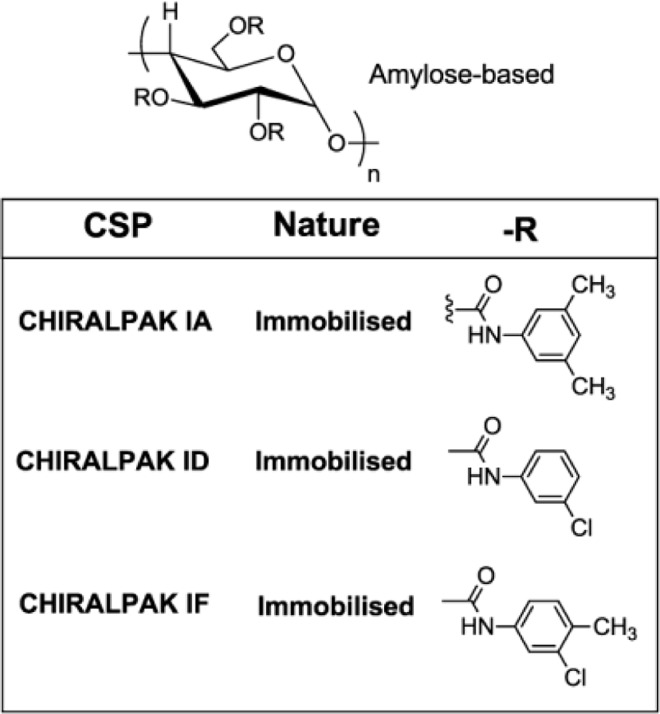
Structures of the chiral selectors present on the amylose-derived CSPs.

In previous report we studied in detail analytical conditions for chiral analysis of various 3,5-dihydro-1,2-oxazines prepared on solid support.[29] For study of enantiomers resolution of oxazines **11(R**^**1**^**,X,R**^**2**^**)** and **12(R**^**1**^**,X,R**^**2**^**)** as well as lactones **8(R**^**1**^**,X)** we used this knowledge and chose the Chiracel columns CHIRALPAK IA, CHIRALPAK ID and CHIRALPAK IF, where the sorbents are based on amylose (see Fig 8).

The HPLC separation conditions were studied for selected representatives of oxazines **11(R**^**1**^**,X,R**^**2**^**)** and **12(R**^**1**^**,X,R**^**2**^**)** (see Table 1). The analytical conditions were first optimized for pure compounds to achieve sufficient resolution and as fast analysis as possible. In the beginning of the study we performed screening of columns by mixture of hexane/alcohol. For this purpose ethanol and isopropanol were tested and the results showed the use of ethanol with lower elution solvent power more suitable for the analysis. Ethanol provided good enantiomers separations, but long time analysis was needed. While, the use of isopropanol decreased the time of analysis, but the resolution was too low. From this reason ethanol was used for majority of the HPLC analyses. Very important role was played by TFA used as an additive. In all cases mobile phase without this additive afforded the analysis with broad and hardly detectable peaks. However, the adding of TFA to the mobile phase significantly improved the separation and peak shapes.

**Table 1:** The results of chiral HPLC separation of oxazines 11(R^1^,X,R^2^) and 12(R^1^,X,R^2^) and lactones 8(R^1^,X).^[a]^

Product	Column	Mobile phase mixture	Proportion	Time of analysis (min)	Rs	α
**12(3,1,1)**	IA	Hexane/EtOH/TFA	85/15/0.1	60	0.64	1.08
**12(1,1,3)**	ID	A:Hexane B:2-PrOH	Gradient 30–50%	35	2.47	1.44
**12(4,1,1)**	IA, ID, IF	Hexane/EtOH/TFA	various	45	—	—
**11(3,1,1)**	IA	A:Hexane B:EtOH	Gradient 30–50%	60	2.50	1.47
**11(1,1,3)**	IA	Hexane/EtOH/TFA	75/25/0.1	22	0.90	1.18
**11(4,1,1)**	IA	Hexane/EtOH/TFA	50/50/0.1	60	1.37	1.49
**8(2,1)**	IF	A:Hexane/TFA B:EtOH	Gradient 30–50%	35	2.22	5.90
**8(4,1)**	IF	A:Hexane/TFA B:EtOH	Gradient 30–50%	35	3.83	4.32
**8(3,1)**	IA	Hexane/EtOH/TFA	60/40/0,1	55	3.98	2.51
**8(1,1)**	IA	A:Hexane/TFA B:EtOH	Gradient 40–50%	35	7.15	4.07
**8(1,2)**	ID	A:Hexane/TFA B:EtOH	Gradient 40–50%	30	3.05	3.5

[a] In all cases the two peaks of enantiomers were observed. The only exception is derivative **12(4,1,1)**, where enantiomers were not separated.

When comparing columns IA and ID, which have the same dimension, the column IA provided the elution of the sample in lower retention time. Therefore column IA was more preferable in the majority of the separations. Since in one case of derivative **12(4,1,1)** enantiomers were not resolved with any of these two columns, another column using 3-chloro-4-methylaniline as a sorbent (column IF–see Fig 8), successfully applied in separation of lactones **8(R**^**1**^**,X)** (see later) was included to the screening. Unfortunately, the sufficient resolution was accomplished neither with this column. The HPLC conditions for chiral separation tested for selected derivatives of the compounds **11(R**^**1**^**,X,R**^**2**^**)**, **12(R**^**1**^**,X,R**^**2**^**)** and **8(R**^**1**^**,X)** are summarized in Table 1.

**Fig 9:**
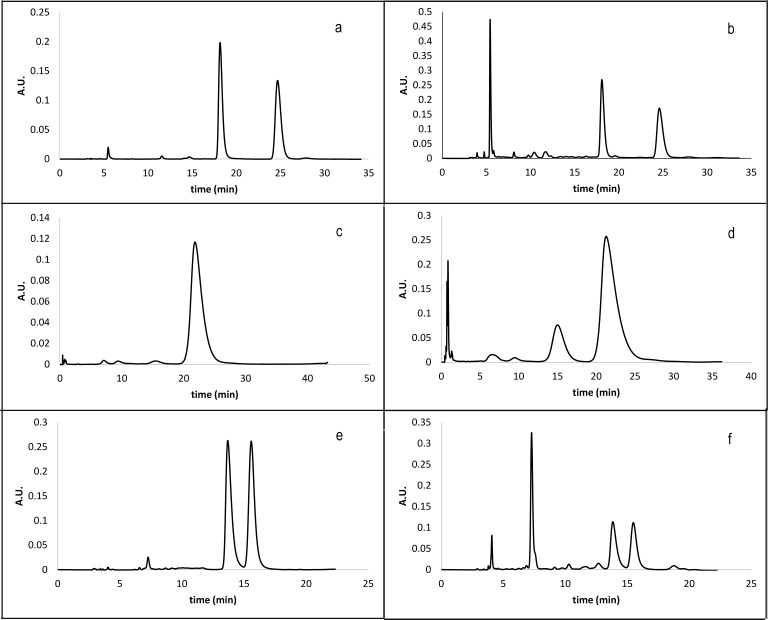
Examples of HPLC chromatograms of pure and crude oxazines 11(R^1^,X,R^2^) and 12(R^1^,X,R^2^). a) **12(1,1,3)** pure compound; b) **12(1,1,3)** crude mixture; c) **12(4,1,1)** pure compound (no chiral separation); d) **12(4,1,1)** crude mixture (no chiral separation); e) **11(1,1,3)** pure compound; f) **11(1,1,3)** crude mixture.

For future development of conditions for stereoselective synthesis of the mentioned oxazines it is necessary to have an analytical method robust and fast enough also for monitoring of stereoisomers in the reaction. Therefore we tried to apply the developed analytical conditions for separation of enantiomers of derivatives **11(R**^**1**^**,X,R**^**2**^**)** and **12(R**^**1**^**,X,R**^**2**^**)** in crude reaction mixtures. The chiral separation of derivatives **12(R**^**1**^**,X,R**^**2**^**)** was implemented to the study despite the fact that the amidation of oxazine **5(R**^**1**^**,X)** does not contribute to stereoisomery. But it could do, when HDA reaction is performed with amides of sorbic acid in a future study. For both compounds **11(R**^**1**^**,X,R**^**2**^**)** and **12(R**^**1**^**,X,R**^**2**^**)** we found out, that the separation conditions were suitable also for crude mixtures, when impurities were sufficiently separated (Fig 9).

The HPLC separation afforded good separation for all lactones and two oxazines mixtures. For three racemic mixtures the Rs was lower than standard 1.5 and one derivative was not separated at all (Table 1). The time of the analyses was 22–60 minutes, what limits the applicability in development and monitoring of stereoselective synthesis in high-throughput mode–main advantage of solid-phase synthesis. Therefore we decided to apply supercritical fluid chromatography (SFC), modern method for easier and fast separation especially of chiral compounds. For the optimization study we chose the same sorbent as for HPLC based on amylose. Methanol was used as the organic modifier. The suitable resolution and elution was achieved with alcohol content about 15–60%. As the additive 0.1% of DEA/TFA mixture was supplemented to the mobile phase. Unlike the HPLC, where the derivative **12(4,1,1)** was not possible to separate, the SFC method provided the separation of enantiomers with Rs factor 1.83 (see Table 2).

**Table 2:** The results of chiral SFC separation of oxazines 11(R^1^,X,R^2^) and 12(R^1^,X,R^2^) and lactones 8(R^1^,X).^[a]^

Product	Column	Mobile phase mixture	Proportion	Time of analysis (min)	Rs	α
**12(3,1,1)**	IA3	CO_2_/MeOH/TFA+DEA	70/30/0.1	7	1.46	1.32
**12(1,1,3)**	ID3	A:CO_2_ B:MeOH/TFA+DEA	Gradient 15–35%	9	0.99	1.06
**12(4,1,1)**	ID3	CO_2_/MeOH/TFA+DEA	60/40/0.1	7	1.83	1.38
**11(3,1,1)**	ID3	A:CO_2_ B:MeOH/TFA+DEA	Gradient 15–35%	9	1.52	1.13
**11(1,1,3)**	IA3	CO_2_/MeOH/TFA+DEA	75/25/0.1	7	0.84	1.16
**11(4,1,1)**	ID3	CO_2_/MeOH/TFA+DEA	60/40/0.1	7	2.17	2.29
**8(2,1)**	ID3	A:CO_2_ B:MeOH/TFA+DEA	Gradient 40–55%	7	2.22	2.45
**8(4,1)**	ID3	A:CO_2_ B:MeOH/TFA+DEA	Gradient 40–55%	7	1.34	2.14
**8(3,1)**	ID3	A:CO_2_ B:MeOH/TFA+DEA	Gradient 30–55%	7	1.70	2.17
**8(1,1)**	ID3	A:CO_2_ B:MeOH/TFA+DEA	Gradient 40–60%	7	5.8	4.76
**8(1,2)**	ID3	A:CO_2_ B:MeOH/TFA+DEA	Gradient 40–55%	7	3.85	3.9

[a] In all cases the two peaks of enantiomers were observed.

**Fig 10:**
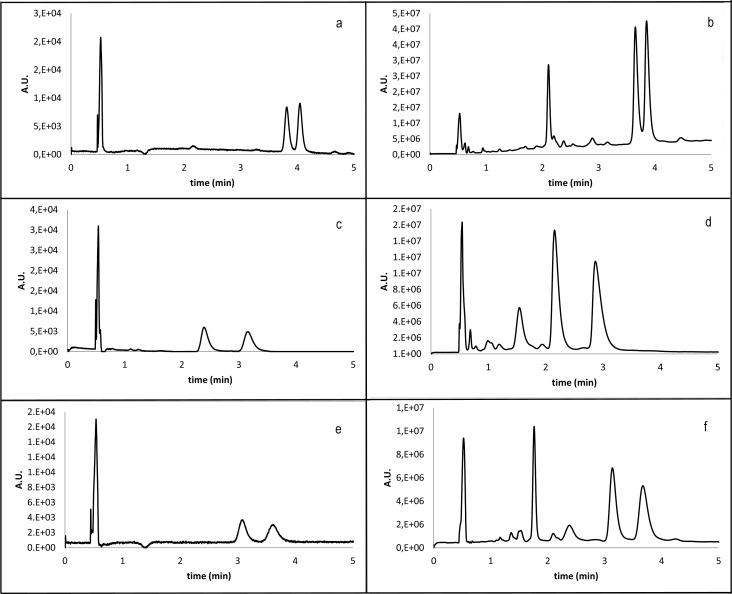
Example of SFC chromatograms of pure and crude mixtures of oxazines 11(R^1^,X,R^2^) and 12(R^1^,X,R^2^). a) **12(1,1,3)** pure compound; b) **12(1,1,3)** crude mixture; c) **12(4,1,1)** pure compound; d) **12(4,1,1)** crude mixture; e) **11(1,1,3)** pure compound; f) **11(1,1,3)** crude mixture.

The time of analyses varied between 7 and 9 minutes what means that the SFC analysis is up to 10x faster than classical HPLC with keeping or improvement of good resolution of peaks of enantiomers. The examples of oxazine separation are shown in Fig 10.

**Fig 11:**
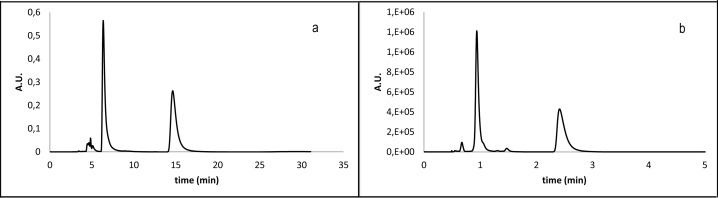
Comparison of HPLC (a) and SFC (b) method for analysis of lactone 8(1,2).

Because the stereoisomery of lactone **8(R**^**1**^**,X,R**^**2**^**)** reflects stereoisomery of derivative **5(R**^**1**^**,X)**, we studied conditions only for pure derivatives **8(R**^**1**^**,X)** to develop method for the final enantiomeric purity control of lactones **8(R**^**1**^**,X)** as the final compounds. Fig 11 demonstrates the rapidity of the SFC method in comparison to HPLC.

## Conclusion

We used solid supported hetero-Diels-Alder cycloaddition of arylnitroso derivatives and sorbic acid for the synthesis of oxazine derivatives and their subsequent transformation. The stereoselective hydroxylation of double bond of oxazine cycle following by N-O cleavage provided trihydroxy precursors for ɤ-lactones cyclization. The lactone formation is keeping configuration of all chiral centers and therefore the method is suitable for stereoselective synthesis of substituted 3,4-dihydroxy-ƴ-lactones. The same precursors are then used for synthesis of derivatives of 3,6-dihydro-1,2-oxazines. For stereoselectivity control analytical methods based on chiral HPLC and SFC separation were developed and compared. While the HPLC analyses suffer from the low versatility of chiral columns, longer time of analysis and generally lower resolution, SFC was found to be able separate all derivatives in very short time with acceptable resolution and application of only two sorbents. The synthetic and analytical conditions are thus possible to use for development of stereoselective synthesis of oxazine as well as ƴ-lactone derivatives and the products of their transformation on solid support.

## Materials and Methods

### Apparatus

HPLC chiral analyses were performed using an HPLC Alliance Waters e2695 system equipped with a Waters PDA detector 2998 and several 250 x 4.6 mm (i.d.) Chiracel columns, including CHIRALPAK IA, CHIRALPAK ID and CHIRALPAK IF with sorbents based on amylose immobilized on 5 μm silica particles. Different mobile phase systems were investigated in this study. The eluting strength of the mobile phases was adjusted in such a way so that each racemic compound could be eluted within a reasonable time window. The proportion of each mobile phase component or mobile phase additive was always measured by volume. The chromatographic runs were performed at a flow rate of 1.0 ml/min and at a column temperature of 25°C.

SFC chiral analyses were performed using an Acquity UPC2 system (Waters) consisting of a binary solvent manager, sample manager, column manager, column heater, convergence manager, PDA detector 2998, QDa mass detector and 4.6x100mm, 3 μm particle chiral analytical columns CHIRALPAK IA3 and CHIRALPAK ID3,. The eluting strength of the mobile phases was adjusted in such a way so that each racemic compound could be eluted within a reasonable time window. The chromatographic runs were performed at a flow rate of 2.5 ml/min and at a column temperature of 38°C.

LC/MS analyses were performed by UHPLC/MS using an Acquity UHPLC chromatograph equipped with a PDA detector and a single quadrupole mass spectrometer (Waters) with an X-Select C18 column at 30°C at a flow rate of 600 μl min^-1^. The mobile phase consisted of (A) 0.01 M ammonium acetate in water and (B) acetonitrile, with B linearly programmed to shift from 10% to 80% over the course of 2.5 min and then maintained this concentration for 1.5 min. The column was re-equilibrated at 10% B for 1 min. The ESI ionization operated at a discharge current of 5 μA, a vaporizer temperature of 350°C and a capillary temperature of 200°C.

Purification was performed by semipreparative HPLC using a Waters 1500 series HPLC equipped with an Autosampler 2707, a Binary HPLC pump 1525, a Waters Photodiode Array Detector 2998 and a Waters Fraction Collector III with a 20x100 mm, 5 μm particle YMC C18 reverse phase column. The mobile phase consisted of acetonitrile and a 10 mM aqueous ammonium acetate gradient over 6 min.

NMR spectra were measured in DMSO-*d6* using a Jeol ECX-500SS (500 MHz, ^1^H) spectrometer and Jeol ECA-400 II (400 MHz, ^1^H). Chemical shifts (δ) are reported in parts per million (ppm) relative to DMSO resonance signal, and coupling constants (*J*) are reported in Hertz (Hz).

HRMS analysis was performed using an Orbitrap Elite high-resolution mass spectrometer (Thermo Fischer Scientific, MA,USA) operating in positive full scan mode (120 000 FWMH) with a mass range of 200–900 m/z. The settings for electrospray ionization were as follows: an oven temperature of 300°C, a sheath gas of 8 arb. units and a source voltage of 1.5 kV. The acquired data were internally calibrated with diisooctyl phthalate as a contaminant in methanol (m/z 391.2843). Samples were diluted to a final concentration of 20 μmol l^-1^ with 0.1% formic acid in water and methanol (50: 50, v/v). The samples were injected by direct infusion into the mass spectrometer.

### Sample preparation

Samples for UHPLC/MS analysis were prepared by treatment of the analytical sample of the resin (~ 5 mg) with cleavage cocktail 50% TFA in DCM for 30 min. The cleavage cocktail was evaporated by a stream of nitrogen and sample was extracted into 1 mL of 50% MeOH/water and analyzed by LC/MS.

For HPLC purification of final compounds, resin (~ 0.5 g) was treated with cleavage cocktail 50% TFA in DCM for 1 hour. The solution was collected and the resin was washed 3 times with 50% TFA in DCM. The combined extracts were evaporated under a stream of nitrogen and dissolved in MeOH/DMSO (4/1).

Crude samples for HPLC chiral analysis and SFC chiral analysis were prepared by cleaving 20 mg of dried resin in 50% TFA in DCM for 30 minutes and evaporating the solvent with a stream of nitrogen. The samples were then extracted into 1 mL of HPLC grade EtOH/hexane (1:1) or EtOH/THF (1:1) for MS analysis and filtered before being used for chiral analysis.

Pure samples for HPLC chiral analysis and SFC chiral analysis were prepared at a concentration of 1mg/mL in HPLC grade EtOH/hexane (1:1).

### Synthetic procedures

#### Amino acid (dipeptide) linker (resin 1(R^1^))

Rink resin (1 g) was washed 3x with DCM and 3 x with DMF, treated with 50% piperidine in DMF and the slurry was shaken for 15 min. The resin was washed 5 x with DMF and 3 x with DCM and the solution of amino acid (2 mmol), HOBt·H_2_O (2 mmol) and DIC (2 mmol) in DMF/DCM (10 mL, 1:1) was added and the slurry was shaken for 3 h. The resin was washed 3 x with DMF and 3 x with DCM. For the synthesis of dipeptide **1(4)** the procedure was repeated to bind the second amino acid.

#### Acylation with carboxylic acid (resin 2(R^1^,X))

The resin **1(R**^**1**^**)** (1 g) was washed 3 x with DCM and 3 x with DMF and treated with 50% piperidine in DMF for 15 min. The resin was washed 5 x with DMF and 3 x with DCM and a solution of carboxylic acid (3 mmol), HOBt·H_2_O (3 mmol) and DIC (3 mmol) in DMF (10 mL) was added. The resin slurry was shaken overnight. Then the resin was washed 3 x with DMF and 3 x with DCM.

Resins 2(1,1) and 2(1,2) were prepared according to previously described procedure.[9]

#### Substitution with hydroxylamine hydrochloride (resin 3(R^1^,X))

Resins **3(R**^**1**^**,1)** were prepared according to previously described procedure.[27]

The appropriate resin **3(R**^**1**^**,2)** was prepared from appropriate resin **2(R**^**1**^**,2)** (1 g), which was washed 3x with DCM and 3 x with DMSO and treated with a solution of NH_2_OH·HCl (14 mmol) and DBU (6 mmol) in DMSO (10 mL). The slurry was shaken at 80°C for 20 h. The resin was washed 3 x with DMSO, 3 x with DMF and 3 x with DCM.

#### HDA reaction with sorbic acid (resin 5(R^1^,X))

Resin **3(R**^**1**^**,X)** (1 g) was washed 3 x with DCM and treated with a solution of sorbic acid (3.5 mmol) and TBAPI (1.75 mmol) in DCM (10 mL). The slurry was shaken for 3 h at room temperature. The resin was washed 3 x with DCM, 3 x with THF and 3 x with DCM.

#### Dihydroxylation of alkene bond (resin 6(R^1^,X) and resin 10(R^1^,X, R^2^))

Resin **5(R**^**1**^**,X)** or **9(R**^**1**^**,X,R**^**2**^**)** was washed 3 x with DCM and treated with a solution of OsO_4_ 2.5 wt. % in *tert*-butanol (0.25 mL) and 4-methylmorpholine *N*-oxide 50% wt. in water (0.75 mL) in DCM (10 mL). The slurry was shaken overnight at room temperature. The resin was washed 5 x with DCM.

#### N-O bond cleavage (resin 7(R^1^,X))

Resin **6(R**^**1**^**,X)** (1 g) was washed 3 x with DCM and 3 x with DMF and treated with a solution of [Mo(CO)_6_] (2 mmol) in H_2_O/DMF (10 mL, 1:9). The slurry was heated at 100°C for 1 h. The resin was washed 5 x with DMF and 3 x with DCM.

#### Amidation of carboxylic acid (resin 9(R^1^,X,R^2^))

Resin **5(R**^**1**^**,X)** (1 g) was washed 3 x with DCM and 3 x with DMF and treated with a solution of amine (3 mmol), HOBt·H_2_O (3 mmol), DIC (3 mmol) and DMAP (0.5 mmol) in DMF/DCM (10 mL, 1:1). The resin slurry was shaken for 24 h at room temperature. The resin was washed 3 x with DCM, 3 x with DMF and 3 x with DCM.

#### Preparation of the lactone (8(R^1^,X))

Lactones **8(R**^**1**^**,X)** were formed during the cleavage of resin **7(R**^**1**^**,X)** under the standard conditions (see HPLC purification of final compounds in section Sample preparation).

#### Preparation of the compound 11(R^1^,X,R^2^)

Resin **9(R**^**1**^**,X,R**^**2**^**)** was cleaved under standard conditions (see HPLC purification of final compounds in section Sample preparation).

#### Preparation of the compound 12(R^1^,X,R^2^)

Resin **10(R**^**1**^**,X,R**^**2**^**)** was cleaved under standard conditions (see HPLC purification of final compounds in section Sample preparation).

### Analytical data

#### 4-((1-(3,4-Dihydroxy-5-oxotetrahydrofuran-2-yl)ethyl)amino)-3-nitrobenzamide 8(1,1)

Yield 14 mg (29%). ^1^H NMR (500 MHz, DMSO*-d*_6_, 25°C, TMS): δ = 1.25 (d, *J* = 6.5 Hz, 3 H, CH_3_), 4.08 (d, *J* = 5.3 Hz, 1 H, C^3^-H), 4.25–4.32 (m, 1H, C^6^-H), 4.38 (d, *J* = 7.4 Hz, 1 H, C^2^-H), 4.44 (d, *J* = 5.3 Hz, 1 H, C^4^-H), 5.49 (br. s., 1 H, OH), 5.82 (br. s., 1 H, OH), 7.22 (d, *J* = 9.2 Hz, 1 H, C^9^-H), 7.28 (br. s., 1 H, NH_2_), 7.95–7.99 (m, C^10^-H, NH_2_), 8.09 (d, *J* = 9.2 Hz, 1 H, NH), 8.62 ppm (d, *J* = 2.1 Hz, 1 H, C^12^-H); ^13^C NMR (126 MHz, DMSO*-d*_6_, 25°C, TMS): δ = 17.3(C^17^), 48.7(C^6^), 68.6(C^3,4^), 87.1(C^2^), 115.0(C^9^), 122.0(C^11^), 127.1(C^12^), 131.5(C^13^), 135.5(C^10^), 146.1(C^8^), 166.3(C^18^), 176.1(C^5^) ppm; HRMS (ESI): *m/z* calcd for C_13_H_15_N_3_O_7_+H^+^: 326.0983 [*M* + H] ^+^; found 326.0983.

The structure of the compound is available in Supporting Information–S42 Fig.

#### *N*-(2-Amino-2-oxoethyl)-4-((1-(3,4-dihydroxy-5-oxotetrahydrofuran-2-yl)ethyl)amino)-3-nitrobenzamide 8(2,1)

Yield 22 mg (38%). ^1^H NMR (500 MHz, DMSO*-d*_6_, 25°C, TMS): δ = 1.29 (d, *J* = 6.3 Hz, 3 H), 3.79 (d, *J* = 6.3 Hz, 2 H), 4.12 (t, *J* = 5.2 Hz, 1 H), 4.37–4.31 (m, 1 H), 4.43 (d, *J* = 7.5 Hz, 1 H), 4.48 (dd, *J* = 6.9, 5.2 Hz, 1H), 5.52 (d, *J* = 4.6 Hz, 1 H), 5.86 (d, *J* = 7.5 Hz, 1 H), 7.02 (br. s., 1 H), 7.29 (d, *J* = 9.2 Hz, 1 H), 7.37 (br. s., 1 H), 8.02 (d, *J* = 8.6 Hz, 1 H), 8.15 (d, *J* = 9.2 Hz, 1 H), 8.68 (s, 1 H), 8.76 ppm (t, *J* = 5.7 Hz, 1 H); ^13^C NMR (126 MHz, DMSO*-d*6, 25°C, TMS): δ = 17.3, 42.9, 48.7, 68.6, 87.1, 115.1, 121.8, 126.9, 131.5, 135.4, 146.1, 164.9, 171.6, 176.1 ppm; HRMS calcd for C_15_H_18_N_4_O_8_+H^+^: 383.1197 [*M* + H]^+^; found 383.1198.

#### *N*-(3-Amino-3-oxopropyl)-4-((1-(3,4-dihydroxy-5-oxotetrahydrofuran-2-yl)ethyl)amino)-3-nitrobenzamide 8(3,1)

Yield 15 mg (25%). ^1^H NMR (500 MHz, DMSO*-d*_6_, 25°C, TMS): δ = 1.25 (d, *J* = 6.3 Hz, 3 H), 2.30 (t, *J* = 7.2 Hz, 2 H), 3.35–3.41 (m, 2 H), 4.07 (d, *J* = 5.7 Hz, 1 H), 4.23–4.32 (m, 1 H), 4.38 (d, *J* = 7.5 Hz, 1 H), 4.44 (d, *J* = 5.2 Hz, 1 H), 5.49 (br. s., 1 H), 5.82 (br. s., 1 H), 6.78 (br. s., 1 H), 7.23 (d, *J* = 9.2 Hz, 1 H), 7.30 (br. s., 1 H), 7.95 (dd, *J* = 9.2, 2.3 Hz, 1 H), 8.09 (d, *J* = 9.2 Hz, 1 H), 8.54 (t, *J* = 5.4 Hz, 1 H), 8.60 ppm (d, *J* = 1.7 Hz, 1 H); ^13^C NMR (126 MHz, DMSO*-d*_6_, 25°C, TMS) δ 17.3, 35.5, 36.5, 48.7, 68.6, 87.1, 115.1, 122.1, 126.6, 131.5, 135.2, 146.0, 164.6, 173.1, 176.1 ppm; HRMS calcd for C_16_H_20_N_4_O_8_+H^+^: 397.1354 [*M* + H]^+^; found 397.1352.

#### *N*-(2-((3-Amino-3-oxopropyl)amino)-2-oxoethyl)-4-((1-(3,4-dihydroxy-5-oxotetrahydrofuran-2-yl)ethyl)amino)-3-nitrobenzamide 8(4,1)

Yield 20 mg (30%). ^1^H NMR (500 MHz, DMSO*-d*_6_, 25°C, TMS): δ = 1.29 (d, *J* = 6.3 Hz, 3 H), 2.22 (t, *J* = 7.2 Hz, 2 H), 3.21–3.29 (m, 2 H), 3.80 (d, *J* = 5.7 Hz, 2 H), 4.12 (t, *J* = 4.6 Hz, 1 H), 4.29–4.37 (m, 1 H), 4.43 (d, *J* = 7.4 Hz, 1 H), 4.45 (dd, *J* = 6.9, 5.2 Hz, 1 H), 5.52 (d, *J* = 4.6 Hz, 1 H), 5.86 (d, *J* = 7.4 Hz, 1 H), 6.81 (br. s., 1 H), 7.26–7.37 (m, 2 H), 7.92 (t, *J* = 5.4 Hz, 1 H), 8.02 (d, *J* = 8.0 Hz, 1 H), 8.15 (d, *J* = 9.2 Hz, 1 H), 8.68 (d, *J* = 1.7 Hz, 1 H), 8.80 ppm (t, *J* = 5.7 Hz, 1 H); ^13^C NMR (126 MHz, DMSO*-d*_6_, 25°C, TMS): δ = 17.3, 35.5, 36.5, 48.7, 68.6, 87.1, 115.1, 122.1, 126.6, 131.5, 135.2, 146.0, 164.6, 173.0, 173.1, 176.1 ppm; HRMS calcd for C_18_H_23_N_5_O_9_+H^+^: 454.1569 [*M* + H]^+^; found 454.1569.

#### 6-((1-(3,4-Dihydroxy-5-oxotetrahydrofuran-2-yl)ethyl)amino)nikotinamide 8(1,2)

Yield 8 mg (19%). ^1^H NMR (400 MHz, DMSO*-d*_6_, 25°C, TMS): δ = 1.17 (d, *J* = 6.4 Hz, 3 H), 4.08 (d, *J* = 5.5 Hz, 1 H), 4.12 (d, *J* = 7.8 Hz, 1 H), 4.30–4.39 (m, 1 H), 4.48 (d, *J* = 5.0 Hz, 1 H), 6.51 (d, *J* = 8.7 Hz, 1 H), 7.05 (br. s., 1 H), 7.12 (d, *J* = 8.7 Hz, 1 H), 7.69 (br. s., 1 H), 7.83 (dd, *J* = 8.7, 2.3 Hz, 1 H), 8.52 ppm (d, *J* = 2.3 Hz, 1 H); ^13^C NMR (126 MHz, DMSO*-d*_6_, 25°C, TMS): δ = 17.8, 46.4, 68.8, 88.3, 108.0, 118.7, 136.8, 149.0, 160.0, 167.4, 176.5 ppm; HRMS calcd for C_12_H_15_N_3_O_5_+H^+^: 282.1084 [*M* + H]^+^; found 282.1085.

#### 2-(4-((3-Amino-3-oxopropyl)carbamoyl)-2-nitrophenyl)-4,5-dihydroxy-3-methyl-*N*-propyl-1,2-oxazinane-6-carboxamide 11(3,1,1)

Yield 26 mg (39%). ^1^H NMR (500 MHz, DMSO-*d*_6_, 25°C, TMS): δ = 0.83 (t, *J* = 7.5 Hz, 3 H, C^28^-H_3_), 1.10 (d, *J* = 6.9 Hz, 3 H, C^22^-H_3_), 1.4 (sxt, *J* = 7.5 Hz, 2 H, C^27^-H_2_), 2.30 (t, *J* = 7.2 Hz, 2 H, C^4^-H_2_), 2.94–3.06 (m, 2 H, C^26^-H_2_), 3.34–3.42 (m, 2 H, C^5^-H_2_), 3.71 (t, *J* = 2.9 Hz, 1 H, C^20^-H), 3.85 (dd, *J* = 9.2, 2.9 Hz, 1 H, C^19^-H), 4.04–4.09 (m, 1 H, C^21^-H), 4.25 (d, *J* = 9.2 Hz, 1 H, C^18^-H), 6.79 (br. s., 1 H, NH_2_), 7.22 (d, *J* = 8.6 Hz, 1 H, C^11^-H), 7.31 (br. s., 1 H, NH_2_), 7.79 (t, *J* = 5.7 Hz, 1 H, N^25^-H), 7.93 (dd, *J* = 9.2, 1.7 Hz, 1 H, C^10^-H), 8.05 (d, *J* = 1.7 Hz, 1 H, C^15^-H), 8.56 ppm (t, *J* = 5.4 Hz, 1 H, N^6^-H); ^13^C NMR (126 MHz, DMSO*-d*_6_, 25°C, TMS): δ = 11.5(C^28^), 11.8(C^22^), 22.8(C^27^), 35.4(C^4^), 36.6(C^5^), 40.7(C^26^), 59.5(C^21^), 65.4(C^19^), 70.0(C^20^), 80.1(C^18^), 116.9(C^11^), 124.4(C^15^), 127.3(C^9^), 131.6(C^10^), 140.2(C^13^), 142.0(C^12^), 164.3(C^7^), 167.1(C^23^), 173.0(C^2^) ppm; HRMS calcd for C_19_H_27_N_5_O_8_+H^+^: 454.1932 [*M* + H]^+^; found 454.1933.

The structure of the compound is available in Supporting Information–S43 Fig.

#### 2-(4-((2-Amino-2-oxoethyl)carbamoyl)-2-nitrophenyl)-*N*-benzyl-4,5-dihydroxy-3-methyl-1,2-oxazinane-6-carboxamide 11(2,1,2)

Yield 25 mg (34%). ^1^H NMR (500 MHz, DMSO-*d*_6_, 25°C, TMS): δ = 1.14 (d, *J* = 6.9 Hz, 3 H), 3.74–3.77 (m, 3 H), 3.90 (d, *J* = 8.6 Hz, 1 H), 4.09–4.15 (m, 1 H), 4.29 (d, *J* = 6.3 Hz, 2 H), 4.38 (d, *J* = 9.2 Hz, 1 H), 4.86 (br. s., 1 H), 5.08 (br. s., 1 H), 7.00 (br. s., 1 H), 7.17–7.29 (m, 6 H), 7.35 (br. s., 1 H), 7.97 (dd, *J* = 8.6, 1.7 Hz, 1 H), 8.13 (d, *J* = 1.7 Hz, 1 H), 8.43 (t, *J* = 6.0 Hz, 1 H), 8.75 ppm (t, *J* = 5.7 Hz, 1 H); ^13^C NMR (126 MHz, DMSO*-d*_6_, 25°C, TMS): δ = 11.4, 42.4, 43.0, 59.5, 65.6, 70.0, 80.2, 116.7, 124.8, 126.9, 127.1, 127.4, 128.6, 131.9, 139.6, 140.1, 142.0, 164.7, 167.4, 171.4 ppm; HRMS calcd for C_22_H_25_N_5_O_8_+H^+^: 488.1776 [*M* + H]^+^; found 488.1778.

#### 4-(4,5-Dihydroxy-3-methyl-6-(piperidine-1-carbonyl)-1,2-oxazinan-2-yl)-3-nitrobenzamide 11(1,1,3)

Yield 25 mg (41%). ^1^H NMR (500 MHz, DMSO-*d*_6_, 25°C, TMS): δ = 1.08 (d, *J* = 6.9 Hz, 3 H,), 1.38–1.61 (m, 6 H), 3.33–3.42 (m, 2 H), 3.45–3.51 (m, 2 H), 3.76 (m, 1 H), 3.87–3.92 (m, 1 H), 4.06–4.08 (m, 1 H), 4.82 (d, *J* = 9.2 Hz, 1 H), 5.01 (d, *J* = 6.9 Hz, 1 H), 5.04 (d, *J* = 4.0 Hz, 1 H), 7.22 (d, *J* = 8.6 Hz, 1 H), 7.41 (br. s., 1 H), 7.96 (dd, *J* = 8.9, 2.0 Hz, 1 H), 8.00 (br. s., 1 H), 8.05 ppm (d, *J* = 2.3 Hz, 1 H); ^13^C NMR (126 MHz, DMSO*-d*_6_, 25°C, TMS): δ = 11.6, 24.6, 25.9, 26.4, 42.9, 46.8, 59.5, 66.1, 70.3, 75.6, 117.2, 124.6, 131.9, 142.0, 165.7, 166.0 ppm; HRMS calcd for C_18_H_24_N_4_O_7_+H^+^: 409.1718 [*M* + H]^+^; found 409.1717.

#### 2-(4-((2-((3-Amino-3-oxopropyl)amino)-2-oxoethyl)carbamoyl)-2-nitrophenyl)-4,5-dihydroxy-3-methyl-*N*-propyl-1,2-oxazinane-6-carboxamide 11(4,1,1)

Yield 35 mg (46%). ^1^H NMR (400 MHz, DMSO-*d*_6_, 25°C, TMS): δ = 0.83 (t, *J* = 7.3 Hz, 3 H), 1.11 (d, *J* = 6.4 Hz, 3 H), 1.33–1.45 (m, 2 H), 2.19 (t, *J* = 7.1 Hz, 2 H), 2.94–3.04 (m, 2 H), 3.21 (q, *J* = 6.9 Hz, 2 H), 3.69–3.73 (m, 1 H), 3.77 (d, *J* = 6.0 Hz, 2 H), 3.85 (dd, *J* = 9.6, 3.2 Hz, 1 H), 4.08–4.11 (m, 1 H), 4.26 (d, *J* = 9.2 Hz, 1 H), 6.79 (br. s., 1 H), 7.23 (d, *J* = 9.2 Hz, 1 H), 7.29 (br. s., 1 H), 7.82 (t, *J* = 5.7 Hz, 1 H), 7.89–7.98 (m, 2 H), 8.09 (d, *J* = 1.8 Hz, 1 H), 8.82 ppm (t, *J* = 6.0 Hz, 1 H); ^13^C NMR (126 MHz, DMSO*-d*_6_, 25°C, TMS): δ = 11.5, 11.8, 22.8, 35.5, 35.7, 40.7, 43.2, 59.4, 65.4, 70.0, 80.1, 116.7, 124.7, 126.8, 131.9, 140.1, 142.1, 164.7, 167.0, 169.2, 173.1 ppm; HRMS calcd for C_21_H_30_N_6_O_9_+H^+^: 511.2147 [*M* + H]^+^; found 511.2145.

#### 2-(5-((2-Amino-2-oxoethyl)carbamoyl)pyridin-2-yl)-4,5-dihydroxy-3-methyl-*N*-propyl-1,2-oxazinane-6-carboxamide 11(2,2,1)

Yield 11 mg (19%). ^1^H NMR (400 MHz, DMSO-*d*_6_, 25°C, TMS): δ = 0.84 (t, *J* = 7.6 Hz, 3 H), 1.11 (d, *J* = 6.9 Hz, 3 H), 1.40–1.47 (m, 2 H), 3.03–3.10 (m, 2 H), 3.73–3.76 (m, 3 H), 3.98 (dd, *J* = 9.6, 3.2 Hz, 1 H), 4.30 (d, *J* = 10.1 Hz, 1 H), 4.78–4.82 (m, 1 H), 6.86 (d, *J* = 8.7 Hz, 1 H), 7.00 (br. s, 1 H), 7.35 (br. s., 1 H), 8.04 (dd, *J* = 8.7, 2.2 Hz, 1 H), 8.28 (t, *J* = 5.5 Hz, 1 H), 8.58 (t, *J* = 6.0 Hz, 1 H), 8.63 ppm (d, *J* = 1.8 Hz, 1 H);^13^C NMR (126 MHz, DMSO*-d*_6_, 25°C, TMS): δ = 11.9, 13.2, 22.7, 40.8, 42.8, 57.1, 65.4, 69.9, 79.0, 107.2, 121.1, 137.5, 148.2, 161.2, 165.5, 167.4, 171.7 ppm; HRMS calcd for C_17_H_25_N_5_O_6_+H^+^: 396.1878 [*M* + H]^+^; found 396.1873.

#### *N*-Benzyl-2-(5-carbamoylpyridin-2-yl)-4,5-dihydroxy-3-methyl-1,2-oxazinane-6-carboxamide 11(1,2,2)

Yield 15 mg (26%). ^1^H NMR (400 MHz, DMSO-*d*_6_, 25°C, TMS): δ = 1.13 (d, *J* = 6.9 Hz, 3 H), 3.76 (t, *J* = 2.9 Hz, 1 H), 4.04 (dd, *J* = 9.9, 3.2 Hz, 1 H), 4.28–4.43 (m, 3 H), 4.82 (m, 1 H), 6.88 (d, *J* = 8.7 Hz, 1 H), 7.18–7.34 (m, 6 H), 7.83 (br. s., 1 H), 8.03 (dd, *J* = 8.9, 2.3 Hz, 1 H), 8.62 (d, *J* = 2.3 Hz, 1 H), 8.83 ppm (t, *J* = 6.1 Hz, 1 H); ^13^C NMR (126 MHz, DMSO*-d*_6_, 25°C, TMS): δ = 13.3, 42.5, 57.1, 65.4, 69.9, 79.0, 107.1, 121.2, 127.3, 127.6, 128.8, 137.7, 139.6, 148.5, 161.3, 167.0, 167.7 ppm; HRMS calcd for C_19_H_22_N_4_O_5_+H^+^: 387.1663 [*M* + H]^+^; found 387.1666.

#### *N*-(3-((2-Amino-2-oxoethyl)amino)-3-oxopropyl)-6-(4,5-dihydroxy-3-methyl-6-(piperidine-1-carbonyl)-1,2-oxazinan-2-yl)nikotinamide 11(4,2,3)

Yield 12 mg (16%). ^1^H NMR (400 MHz, DMSO-*d*_6_, 25°C, TMS): δ = 1.05 (d, *J* = 6.9 Hz, 3 H), 1.43–1.49 (m, 2 H), 1.51–1.60 (m, 4 H), 2.37 (t, *J* = 7.1 Hz, 2 H), 3.38–3.61 (m, 8 H), 3.77 (t, *J* = 2.8 Hz, 1 H), 4.08 (dd, *J* = 9.6, 3.2 Hz, 1 H), 4.74–4.80 (m, 2 H), 6.85 (d, *J* = 8.7 Hz, 1 H), 7.02 (br. s., 1 H), 7.29 (br. s., 1 H), 8.01 (dd, *J* = 8.9, 2.1 Hz, 1 H), 8.10 (t, *J* = 5.7 Hz, 1 H), 8.38 (t, *J* = 5.5 Hz, 1 H), 8.60 ppm (d, *J* = 1.8 Hz, 1 H); ^13^C NMR (126 MHz, DMSO*-d*_6_, 25°C, TMS): δ = 12.7, 24.6, 25.8, 27.0, 35.9, 36.4, 42.4, 42.9, 46.6, 57.7, 65.1, 70.0, 74.9, 107.7, 121.8, 137.5, 148.1, 161.3, 165.1, 165.2, 171.4, 171.7 ppm; HRMS calcd for C_22_H_32_N_6_O_7_+H^+^: 493.2405 [*M* + H]^+^; found 493.2404.

#### 2-(4-((3-Amino-3-oxopropyl)carbamoyl)-2-nitrophenyl)-3-methyl-*N*-propyl-3,6-dihydro-2*H*-1,2-oxazine-6-carboxamide 12(3,1,1)

Yield 22 mg (35%). ^1^H NMR (500 MHz, DMSO-*d*_6_, 25°C, TMS): δ = 0.79 (t, *J* = 7.5 Hz, 3 H, C^28^-H_3_), 1.17 (d, *J* = 6.3 Hz, 3 H, C^22^-H_3_), 1.38 (sxt, *J* = 7.5 Hz, 2 H, C^27^-H_2_), 2.31 (t, *J* = 7.2 Hz, 2 H, C^4^-H_2_), 2.91–2.97 (m, 1 H, C^26^-H), 3.04–3.10 (m, 1 H, C^26^-H), 3.37–3.43 (m, 2 H, C^5^-H_2_), 4.34–4.40 (m, 1 H, C^21^-H), 4.77–4.82 (m, 1 H, C^18^-H), 5.80–5.86 (m, 1 H, C^19^-H), 6.06 (m, 1 H, C^20^-H), 6.79 (br. s., 1 H, NH_2_), 7.30 (br. s., 1 H, NH_2_), 7.38 (d, *J* = 8.6 Hz, 1 H, C^11^-H), 7.53 (t, *J* = 6.0 Hz, 1 H, N^25^-H), 8.00 (dd, *J* = 8.6, 2.3 Hz, 1 H, C^10^-H), 8.13 (d, *J* = 2.3 Hz, 1 H, C^15^-H), 8.60 ppm (t, *J* = 5.4 Hz, 1 H, N^6^-H); ^13^C NMR (126 MHz, DMSO*-d*_6_, 25°C, TMS): δ = 11.6 (C^28^), 14.8(C^22^), 22.7(C^27^), 35.4(C^4^), 36.6(C^5^), 40.1(C^26^), 53.3(C^21^), 78.8(C^18^), 118.2(C^11^), 124.3(C^15^+C^19^), 128.4(C^9^), 130.2(C^20^), 131.6(C^10^), 140.5(C^13^), 141.5(C^12^), 164.3(C^7^), 166.8(C^23^), 172.9(C^2^) ppm; HRMS calcd for C_19_H_25_N_5_O_6_+H^+^: 420.1878 [*M* + H]^+^; found 420.1900.

The structure of the compound is available in Supporting Information–S44 Fig.

#### 4-(3-Methyl-6-(piperidine-1-carbonyl)-3,6-dihydro-2*H*-1,2-oxazin-2-yl)-3-nitrobenzamide 12(1,1,3)

Yield 17 mg (31%). ^1^H NMR (500 MHz, DMSO-*d*_6_, 25°C, TMS): δ = 1.08 (d, *J* = 6.3 Hz, 3 H), 1.36–1.47 (m, 4 H), 1.47–1.60 (m, 2 H), 3.35–3.46 (m, 4 H), 4.23–4.38 (m, 1 H), 5.31 (d, *J* = 1.7 Hz, 1 H), 5.83–5.85 (m, 1 H), 6.05 (m, 1 H), 7.38 (d, *J* = 9.12 Hz, 1 H), 7.48 (br. s., 1 H), 8.01–8.11 (m, 2 H), 8.17 ppm (d, *J* = 1.7 Hz, 1 H); ^13^C NMR (126 MHz, DMSO*-d*_6_, 25°C, TMS): δ = 15.0, 24.5, 25.8, 26.6, 43.1, 46.4, 54.2, 77.0, 119.0, 124.2, 124.6, 128.9, 129.9, 132.1, 140.8, 142.2, 164.6, 165.9 ppm; HRMS calcd for C_18_H_22_N_4_O_5_+H^+^: 375.1663 [*M* + H]^+^; found 375.1614.

#### 2-(4-((2-Amino-2-oxoethyl)carbamoyl)-2-nitrophenyl)-*N*-benzyl-3-methyl-3,6-dihydro-2*H*-1,2-oxazine-6-carboxamide 12(2,1,2)

Yield 22 mg (33%). ^1^H NMR (400 MHz, DMSO-*d*_6_, 25°C, TMS): δ = 1.20 (d, *J* = 6.2 Hz, 3 H), 3.77 (d, *J* = 6.2 Hz, 2 H), 4.18 (dd, *J* = 15.3, 5.7 Hz, 1 H), 4.34 (dd, *J* = 15.5, 6.3 Hz, 1 H), 4.38–4.45 (m, 1 H), 4.89 4.91 (m, 1 H), 5.85–5.87 (m, 1 H), 6.07–6.14 (m, 1 H), 7.03 (br. s., 1 H), 7.17–7.22 (m, 3 H), 7.25–7.31 (m, 2 H), 7.39 (br. s., 1 H), 7.42 (d, *J* = 8.7 Hz, 1 H), 8.04 (dd, *J* = 8.8, 2.0 Hz, 1 H), 8.20 (d, *J* = 1.8 Hz, 1 H), 8.26 (t, *J* = 6.1 Hz, 1 H), 8.82 ppm (t, *J* = 6.0 Hz, 1 H); ^13^C NMR (126 MHz, DMSO*-d*_6_, 25°C, TMS): δ = 14.7, 42.2, 43.0, 53.2, 78.9, 118.1, 124.1, 124.6, 127.2, 127.3, 128.0, 128.7, 130.5, 131.8, 139.7, 140.4, 141.6, 164.6, 167.1, 171.4 ppm; HRMS calcd for C_22_H_23_N_5_O_6_+H^+^: 454.1721 [*M* + H]^+^; found 454.1720.

#### 2-(4-((2-((3-Amino-3-oxopropyl)amino)-2-oxoethyl)carbamoyl)-2-nitrophenyl)-3-methyl-*N*-propyl-3,6-dihydro-2*H*-1,2-oxazine-6-carboxamide 12(4,1,1)

Yield 21 mg (29%). ^1^H NMR (500 MHz, DMSO-*d*_6_, 25°C, TMS): δ = 0.79 (t, *J* = 7.5 Hz, 3 H), 1.18 (d, *J* = 6.3 Hz, 3 H), 1.38 (sxt, *J* = 7.5 Hz, 2 H), 2.19 (t, *J* = 7.2 Hz, 2 H), 2.91–2.97 (m, 1 H), 3.04–3.10 (m, 1 H), 3.21 (m, 2 H), 3.75–3.80 (m, 2 H), 4.35–4.44 (m, 1 H), 4.76–4.83 (m, 1 H), 5.79–5.87 (m, 1 H), 6.02–6.12 (m, 1 H), 6.78 (br. s., 1 H), 7.28 (br. s., 1 H), 7.40 (d, *J* = 8.6 Hz, 1 H), 7.54 (t, *J* = 6.0 Hz, 1 H), 7.93 (t, *J* = 5.7 Hz, 1 H), 8.03 (dd, *J* = 8.9, 2.00 Hz, 1 H), 8.17 (d, *J* = 1.7 Hz, 1 H), 8.84 ppm (t, *J* = 6.0 Hz, 1 H); ^13^C NMR (126 MHz, DMSO*-d*_6_, 25°C, TMS): δ = 11.6, 14.8, 22.8, 35.5, 35.7, 41.0, 43.2, 53.2, 78.8, 118.1, 124.3, 124.5, 127.9, 130.2, 131.8, 140.4, 141.6, 164.6, 166.8, 169.2, 173.0 ppm; HRMS calcd for C_21_H_28_N_6_O_7_+H^+^: 477.2092 [*M* + H]^+^; found 477.2091.

#### 2-(5-((2-Amino-2-oxoethyl)carbamoyl)pyridin-2-yl)-3-methyl-*N*-propyl-3,6-dihydro-2*H*-1,2-oxazine-6-carboxamide 12(2,2,1)

Yield 14 mg (25%). ^1^H NMR (500 MHz, DMSO-*d*_6_, 25°C, TMS): δ = 0.81 (t, *J* = 7.5 Hz, 3 H), 1.17 (d, *J* = 6.3 Hz, 3 H), 1.44 (sxt, *J* = 7.2 Hz, 2 H), 3.00–3.13 (m, 2 H), 3.77 (d, *J* = 6.3 Hz, 2 H), 4.86–4.91 (m, 1 H), 4.97–4.99 (m, 1 H), 5.85–5.90 (m, 1 H), 6.05–6.10 (m, 1 H), 6.99 (br. s., 1 H), 7.21 (d, *J* = 8.6 Hz, 1 H), 7.34 (br. s., 1 H), 8.00 (t, *J* = 6.0 Hz, 1 H), 8.11 (dd, *J* = 8.6, 2.3 Hz, 1 H), 8.61 (t, *J* = 6.0 Hz, 1 H), 8.70 ppm (d, *J* = 1.7 Hz, 1 H); ^13^C NMR (126 MHz, DMSO*-d*_6_, 25°C, TMS): δ = 11.8, 16.4, 22.8, 40.8, 42.8, 51.0, 77.6, 108.5, 122.2, 124.2, 130.5, 137.7, 148.3, 160.7, 165.7, 167.1, 171.6 ppm; HRMS calcd for C_17_H_23_N_5_O_4_+H^+^: 362.1823 [*M* + H]^+^; found 362.1823.

#### *N*-Benzyl-2-(5-carbamoylpyridin-2-yl)-3-methyl-3,6-dihydro-2*H*-1,2-oxazine-6-carboxamide 12(1,2,2)

Yield 16 mg (30%). ^1^H NMR (400 MHz, DMSO-*d*_6_, 25°C, TMS): δ = 1.18 (d, *J* = 6.4 Hz, 3 H), 4.30–4.35 (m, 2 H), 4.86–4.91 (m, 1 H), 5.07–5.09 (m, 1 H), 5.90–5.93 (m, 1 H), 6.07–6.13 (m, 1 H), 7.18–7.33 (m, 7 H), 7.89 (br. s., 1 H), 8.10 (dd, *J* = 8.7, 2.3 Hz, 1 H), 8.60 (t, *J* = 6.0 Hz, 1 H), 8.69 ppm (d, *J* = 2.3 Hz, 1 H); ^13^C NMR (126 MHz, DMSO*-d*_6_, 25°C, TMS): δ = 16.4, 42.5, 51.1, 77.7, 108.7, 122.3, 124.1, 127.3, 127.6, 128.8, 130.8, 137.9, 139.8, 148.5, 160.7, 166.9, 167.4 ppm; HRMS calcd for C_19_H_20_N_4_O_3_+H^+^: 353.1608 [*M* + H]^+^; found 353.1610.

#### *N*-(3-((2-Amino-2-oxoethyl)amino)-3-oxopropyl)-6-(3-methyl-6-(piperidine-1-carbonyl)-3,6-dihydro-2*H*-1,2-oxazin-2-yl)nikotinamide 12(4,2,3)

Yield 14 mg (20%). ^1^H NMR (500 MHz, DMSO-*d*_6_, 25°C, TMS): δ = 1.09 (d, *J* = 6.3 Hz, 3 H), 1.47–1.52 (m, 2 H), 1.59–1.65 (m, 4 H), 2.38 (t, *J* = 7.2 Hz, 2 H), 3.40–3.48 (m, 4 H), 3.52–3.67 (m, 4 H), 4.79–4.85 (m, 1 H), 5.47–5.50 (m, 1 H), 5.86–5.90 (m, 1 H), 6.05–6.08 (m, 1 H), 7.00 (m, 2 H), 7.26 (br. s., 1 H), 8.04–8.11 (m, 2 H), 8.41 (t, *J* = 5.7 Hz, 1 H), 8.66 ppm (d, *J* = 2.3 Hz, 1 H); ^13^C NMR (126 MHz, DMSO*-d*_6_, 25°C, TMS): δ = 15.7, 24.5, 25.8, 26.9, 35.9, 36.5, 42.4, 43.0, 46.6, 51.5, 76.5, 108.0, 122.5, 124.4, 129.7, 137.8, 148.2, 160.7, 164.6, 165.2, 171.4, 171.7 ppm; HRMS calcd for C_22_H_30_N_6_O_5_+H^+^: 459.2350 [*M* + H]^+^; found 459.2349.

## Supporting Information

S1 Fig HPLC chromatograms of pure and crude oxazines 11 and 12. a) **12(3,1,1)** pure compound; b) **12(3,1,1)** crude mixture; c) **11(3,1,1)** pure compound; d) **11(3,1,1)** crude mixture; e) **11(4,1,1)** pure compound; f) **11(4,1,1)** crude mixture.
(TIF)

S2 Fig SFC chromatograms of pure and crude oxazines 11 and 12. a) **12(3,1,1)** pure compound; b) **12(3,1,1)** crude mixture; c) **11(3,1,1)** pure compound; d) **11(3,1,1)** crude mixture; e) **11(4,1,1)** pure compound; f) **11(4,1,1)** crude mixture.
(TIF)

S3 Fig HPLC and SFC chromatograms of lactones 8. a) **8(2,1)** HPLC; b) **8(2,1)** SFC; c) **8(4,1)** HPLC; d) **8(4,1)** SFC; e) **8(3,1)** HPLC; f) **8(3,1)** SFC; g) **8(1,1)** HPLC; h) **8(1,1)** SFC.
(TIF)

S4 Fig ^1^H NMR spectrum of 4-((1-(3,4-Dihydroxy-5-oxotetrahydrofuran-2-yl)ethyl)amino)-3-nitrobenzamide 8(1,1). (TIF)

S5 Fig ^13^C NMR spectrum of 4-((1-(3,4-Dihydroxy-5-oxotetrahydrofuran-2-yl)ethyl)amino)-3-nitrobenzamide 8(1,1). (TIF)

S6 Fig ^1^H NMR spectrum of *N*-(2-Amino-2-oxoethyl)-4-((1-(3,4-dihydroxy-5-oxotetrahydrofuran-2-yl)ethyl)amino)-3-nitrobenzamide 8(2,1). (TIF)

S7 Fig ^13^C NMR spectrum of *N*-(2-Amino-2-oxoethyl)-4-((1-(3,4-dihydroxy-5-oxotetrahydrofuran-2-yl)ethyl)amino)-3-nitrobenzamide 8(2,1). (TIF)

S8 Fig ^1^H NMR spectrum of *N*-(3-Amino-3-oxopropyl)-4-((1-(3,4-dihydroxy-5-oxotetrahydrofuran-2-yl)ethyl)amino)-3-nitrobenzamide 8(3,1). (TIF)

S9 Fig ^13^C NMR spectrum of *N*-(3-Amino-3-oxopropyl)-4-((1-(3,4-dihydroxy-5-oxotetrahydrofuran-2-yl)ethyl)amino)-3-nitrobenzamide 8(3,1). (TIF)

S10 Fig ^1^H NMR spectrum of *N*-(2-((3-Amino-3-oxopropyl)amino)-2-oxoethyl)-4-((1-(3,4-dihydroxy-5-oxotetrahydrofuran-2-yl)ethyl)amino)-3-nitrobenzamide 8(4,1). (TIF)

S11 Fig ^13^C NMR spectrum of *N*-(2-((3-Amino-3-oxopropyl)amino)-2-oxoethyl)-4-((1-(3,4-dihydroxy-5-oxotetrahydrofuran-2-yl)ethyl)amino)-3-nitrobenzamide 8(4,1). (TIF)

S12 Fig ^1^H NMR spectrum of 6-((1-(3,4-Dihydroxy-5-oxotetrahydrofuran-2-yl)ethyl)amino)nikotinamide 8(1,2). (TIF)

S13 Fig ^13^C NMR spectrum of 6-((1-(3,4-Dihydroxy-5-oxotetrahydrofuran-2-yl)ethyl)amino)nikotinamide 8(1,2). (TIF)

S14 Fig ^1^H NMR spectrum of 2-(4-((3-Amino-3-oxopropyl)carbamoyl)-2-nitrophenyl)-4,5-dihydroxy-3-methyl-*N*-propyl-1,2-oxazinane-6-carboxamide 11(3,1,1). (TIF)

S15 Fig ^13^C NMR spectrum of 2-(4-((3-Amino-3-oxopropyl)carbamoyl)-2-nitrophenyl)-4,5-dihydroxy-3-methyl-*N*-propyl-1,2-oxazinane-6-carboxamide 11(3,1,1). (TIF)

S16 Fig ^1^H NMR spectrum of 2-(4-((2-Amino-2-oxoethyl)carbamoyl)-2-nitrophenyl)-*N*-benzyl-4,5-dihydroxy-3-methyl-1,2-oxazinane-6-carboxamide 11(2,1,2). (TIF)

S17 Fig ^13^C NMR spectrum of 2-(4-((2-Amino-2-oxoethyl)carbamoyl)-2-nitrophenyl)-*N*-benzyl-4,5-dihydroxy-3-methyl-1,2-oxazinane-6-carboxamide 11(2,1,2). (TIF)

S18 Fig ^1^H NMR spectrum of 4-(4,5-Dihydroxy-3-methyl-6-(piperidine-1-carbonyl)-1,2-oxazinan-2-yl)-3-nitrobenzamide 11(1,1,3). (TIF)

S19 Fig ^13^C NMR spectrum of 4-(4,5-Dihydroxy-3-methyl-6-(piperidine-1-carbonyl)-1,2-oxazinan-2-yl)-3-nitrobenzamide 11(1,1,3). (TIF)

S20 Fig ^1^H NMR spectrum of 2-(4-((2-((3-Amino-3-oxopropyl)amino)-2-oxoethyl)carbamoyl)-2-nitrophenyl)-4,5-dihydroxy-3-methyl-*N*-propyl-1,2-oxazinane-6-carboxamide 11(4,1,1). (TIF)

S21 Fig ^13^C NMR spectrum of 2-(4-((2-((3-Amino-3-oxopropyl)amino)-2-oxoethyl)carbamoyl)-2-nitrophenyl)-4,5-dihydroxy-3-methyl-*N*-propyl-1,2-oxazinane-6-carboxamide 11(4,1,1). (TIF)

S22 Fig ^1^H NMR spectrum of 2-(5-((2-Amino-2-oxoethyl)carbamoyl)pyridin-2-yl)-4,5-dihydroxy-3-methyl-*N*-propyl-1,2-oxazinane-6-carboxamide 11(2,2,1). (TIF)

S23 Fig ^13^C NMR spectrum of 2-(5-((2-Amino-2-oxoethyl)carbamoyl)pyridin-2-yl)-4,5-dihydroxy-3-methyl-*N*-propyl-1,2-oxazinane-6-carboxamide 11(2,2,1). (TIF)

S24 Fig ^1^H NMR spectrum of *N*-Benzyl-2-(5-carbamoylpyridin-2-yl)-4,5-dihydroxy-3-methyl-1,2-oxazinane-6-carboxamide 11(1,2,2). (TIF)

S25 Fig ^13^C NMR spectrum of *N*-Benzyl-2-(5-carbamoylpyridin-2-yl)-4,5-dihydroxy-3-methyl-1,2-oxazinane-6-carboxamide 11(1,2,2). (TIF)

S26 Fig ^1^H NMR spectrum of *N*-(3-((2-Amino-2-oxoethyl)amino)-3-oxopropyl)-6-(4,5-dihydroxy-3-methyl-6-(piperidine-1-carbonyl)-1,2-oxazinan-2-yl)nikotinamide 11(4,2,3). (TIF)

S27 Fig ^13^C NMR spectrum of *N*-(3-((2-Amino-2-oxoethyl)amino)-3-oxopropyl)-6-(4,5-dihydroxy-3-methyl-6-(piperidine-1-carbonyl)-1,2-oxazinan-2-yl)nikotinamide 11(4,2,3). (TIF)

S28 Fig ^1^H NMR spectrum of 2-(4-((3-Amino-3-oxopropyl)carbamoyl)-2-nitrophenyl)-3-methyl-*N*-propyl-3,6-dihydro-2*H*-1,2-oxazine-6-carboxamide 12(3,1,1). (TIF)

S29 Fig ^13^C NMR spectrum of 2-(4-((3-Amino-3-oxopropyl)carbamoyl)-2-nitrophenyl)-3-methyl-*N*-propyl-3,6-dihydro-2*H*-1,2-oxazine-6-carboxamide 12(3,1,1). (TIF)

S30 Fig ^1^H NMR spectrum of 4-(3-Methyl-6-(piperidine-1-carbonyl)-3,6-dihydro-2*H*-1,2-oxazin-2-yl)-3-nitrobenzamide 12(1,1,3). (TIF)

S31 Fig ^1^C NMR spectrum of 4-(3-Methyl-6-(piperidine-1-carbonyl)-3,6-dihydro-2*H*-1,2-oxazin-2-yl)-3-nitrobenzamide 12(1,1,3). (TIF)

S32 Fig ^1^H NMR spectrum of 2-(4-((2-Amino-2-oxoethyl)carbamoyl)-2-nitrophenyl)-*N*-benzyl-3-methyl-3,6-dihydro-2*H*-1,2-oxazine-6-carboxamide 12(2,1,2). (TIF)

S33 Fig ^13^C NMR spectrum of -(4-((2-Amino-2-oxoethyl)carbamoyl)-2-nitrophenyl)-*N*-benzyl-3-methyl-3,6-dihydro-2*H*-1,2-oxazine-6-carboxamide 12(2,1,2). (TIF)

S34 Fig ^1^H NMR spectrum of 2-(4-((2-((3-Amino-3-oxopropyl)amino)-2-oxoethyl)carbamoyl)-2-nitrophenyl)-3-methyl-*N*-propyl-3,6-dihydro-2*H*-1,2-oxazine-6-carboxamide 12(4,1,1). (TIF)

S35 Fig ^13^C NMR spectrum of 2-(4-((2-((3-Amino-3-oxopropyl)amino)-2-oxoethyl)carbamoyl)-2-nitrophenyl)-3-methyl-*N*-propyl-3,6-dihydro-2*H*-1,2-oxazine-6-carboxamide 12(4,1,1). (TIF)

S36 Fig ^1^H NMR spectrum of 2-(5-((2-Amino-2-oxoethyl)carbamoyl)pyridin-2-yl)-3-methyl-*N*-propyl-3,6-dihydro-2*H*-1,2-oxazine-6-carboxamide 12(2,2,1). (TIF)

S37 Fig ^13^C NMR spectrum of 2-(5-((2-Amino-2-oxoethyl)carbamoyl)pyridin-2-yl)-3-methyl-*N*-propyl-3,6-dihydro-2*H*-1,2-oxazine-6-carboxamide 12(2,2,1). (TIF)

S38 Fig ^1^H NMR spectrum of *N*-Benzyl-2-(5-carbamoylpyridin-2-yl)-4,5-dihydroxy-3-methyl-1,2-oxazinane-6-carboxamide 12(1,2,2). (TIF)

S39 Fig ^13^C NMR spectrum of *N*-Benzyl-2-(5-carbamoylpyridin-2-yl)-4,5-dihydroxy-3-methyl-1,2-oxazinane-6-carboxamide 12(1,2,2). (TIF)

S40 Fig ^1^H NMR spectrum of *N*-(3-((2-Amino-2-oxoethyl)amino)-3-oxopropyl)-6-(3-methyl-6-(piperidine-1-carbonyl)-3,6-dihydro-2*H*-1,2-oxazin-2-yl)nikotinamide 12(4,2,3). (TIF)

S41 Fig ^13^C NMR spectrum of *N*-(3-((2-Amino-2-oxoethyl)amino)-3-oxopropyl)-6-(3-methyl-6-(piperidine-1-carbonyl)-3,6-dihydro-2*H*-1,2-oxazin-2-yl)nikotinamide 12(4,2,3) (TIF)

S42 Fig Structure of 4-((1-(3,4-Dihydroxy-5-oxotetrahydrofuran-2-yl)ethyl)amino)-3-nitrobenzamide 8(1,1). (TIF)

S43 Fig Structure of 2-(4-((3-Amino-3-oxopropyl)carbamoyl)-2-nitrophenyl)-4,5-dihydroxy-3-methyl-*N*-propyl-1,2-oxazinane-6-carboxamide 11(3,1,1). (TIF)

S44 Fig 2-(4-((3-Amino-3-oxopropyl)carbamoyl)-2-nitrophenyl)-3-methyl-*N*-propyl-3,6-dihydro-2*H*-1,2-oxazine-6-carboxamide 12(3,1,1.) (TIF)

## References

[1] Cesario C, Tardibono LP, Miller MJ. Titanocene(III) Chloride-Mediated Reductions of Oxazines, Hydroxamic Acids, and N-Hydroxy Carbamates. J Org Chem. 2009; 74(1): 448–51. 10.1021/jo802184y 19053586 PMC2725442

[2] Cicchi S, Goti A, Brandi A, Guarna A, De Sarlo F. 1,3-Amino alcohols by reductive cleavage of isoxazolidines with molybdenum hexacarbonyl. Tetrahedron Lett. 1990; 31(23): 3351–4.

[3] Werner L, Hudlicky JR, Wernerova M, Hudlicky T. Synthesis of 1,2- and 1,4-amino alcohols from 1,3-dienes via oxazines. Rearrangements of 1,4-amino alcohol derivatives to oxazolines. Tetrahedron. 2010; 66(21): 3761–9.

[4] Keck GE, Wager TT, McHardy SF. Reductive cleavage of N-O bonds in hydroxylamines and hydroxamic acid derivatives using samarium diiodide. Tetrahedron. 1999; 55(40): 11755–72.

[5] Yekta S, Prisyazhnyuk V, Reissig HU. Simple modifications of enantiopure 1,2-oxazines leading to building blocks for carbohydrate and peptide mimetics. Synlett. 2007; (13): 2069–72.

[6] Dekaris V, Pulz R, Al-Harrasi A, Lentz D, Reissig HU. Stereoselective Syntheses of Aza, Amino and Imino Sugar Derivatives by Hydroboration of 3,6-Dihydro-2H-1,2-oxazines as Key Reaction. European J Org Chem. 2011; (17): 3210–9.

[7] Defoin A, Sarazin H, Strehler C, Streith J. De novo asymmetric synthesis of two 5-amino-5,6-dideoxy-D-allose derivatives. Tetrahedron Lett. 1994; 35(31): 5653–6.

[8] Calvet G, Blanchard N, Kouklovsky C. Domino Metathesis of 3,6-Dihydro-1,2-oxazine: Access to Isoxazolo[2,3-a]pyridin-7-ones. Organic Lett. 2007; 9(8): 1485–8. 10.1021/ol0702066 17352486

[9] Bodnar BS, Miller MJ. The nitrosocarbonyl hetero-Diels-Alder reaction as a useful tool for organic syntheses. Angew Chem Int Ed Engl. 2011; 50(25): 5630–47. 10.1002/anie.201005764 21520360 PMC4079088

[10] Krchnak V, Waring KR, Noll BC, Moellmann U, Dahse HM, Miller MJ. Evolution of Natural Product Scaffolds by Acyl- and Arylnitroso Hetero-Diels-Alder Reactions: New Chemistry on Piperine. J Org Chem. 2008; 73(12): 4559–67. 10.1021/jo8004827 18489157

[11] Krchnak V, Moellmann U, Dahse HM, Miller MJ. Solid-Supported Nitroso Hetero-Diels-Alder Reactions. 3. Acid-Mediated Transformation of Cycloadducts by Scission of the Oxazine C-O Bonds. J Comb Chem. 2008; 10(1): 112–117. 10.1021/cc700142d 18067270

[12] Riediker M, Graf W. Synthetic application of epoxynitrones. II. Synthesis of steroidal -methylidene-Î3-lactones. Helv Chim Acta. 1979; 62(5): 1586–1602.

[13] Ruettimann A, Ginsburg D. Propellanes. XXXII. Preparation of propellane lactones by means of the chloronitrone reaction. Helv Chim Acta. 1975; 58(8): 2237–2239.

[14] Shao LD, Wu YN, Xu J, He J, Zhao Y, Peng LY, et al Synthesis of L-ascorbic acid lactone derivatives. Nat Prod Bioprospect. 2014; 4(3): 181–8. 10.1007/s13659-014-0022-6 24955300 PMC4050306

[15] Amaral PA, Gouault N, Le Roch M, Eifler-Lima VL, David M. Towards synthesis of kavalactone derivatives. Tetrahedron Lett. 2008; 49(47): 6607–9.

[16] Jimenez V, Kemmerling U, Paredes R, Maya JD, Sosa MA, Galanti N. Natural sesquiterpene lactones induce programmed cell death in Trypanosoma cruzi: A new therapeutic target? Phytomedicine. 2014; 21(11): 1411–8. 10.1016/j.phymed.2014.06.005 25022207

[17] Ghantous A, Sinjab A, Herceg Z, Darwiche N. Parthenolide: from plant shoots to cancer roots. Drug Discov Today. 2013; 18(17–18): 894–905. 10.1016/j.drudis.2013.05.005 23688583

[18] Hwang DR, Wu YS, Chang CW, Lien TW, Chen WC, Tan UK, et al Synthesis and anti-viral activity of a series of sesquiterpene lactones and analogues in the subgenomic HCV replicon system. Bioorg Med Chem. 2006; 14(1): 83–91. 10.1016/j.bmc.2005.07.055 16140536

[19] Albrecht A, Albrecht L, Janecki T. Recent Advances in the Synthesis of α-Alkylidene-Substituted δ-Lactones, γ-Lactams and δ-Lactams. European J Org Chem. 2011; (15): 2747–66.

[20] Boucard V, Broustal G, Campagne JM. Synthetic approaches to α,β-unsaturated δ-lactones and lactols. European J Org Chem. 2007; (2): 225–36.

[21] Collins I. Saturated and unsaturated lactones. J Chem Soc Perkin 1. 1999; (11): 1377–96.

[22] Grieco PA. Methods for the synthesis of α-methylene lactones. Synthesis. 1975; (2): 67–82.

[23] Petragnani N, Ferraz HMC, Silva GVJ. Advances in the synthesis of α-methylene lactones. Synthesis. 1986; (3): 157–83.

[24] Ma S, Duan D, Shi Z. Palladium(0)-catalyzed cyclization reaction of polymer-supported aryl iodides with 1,2-allenyl carboxylic acids. A facile solid-phase synthesis of butenolides. Organic Lett. 2000; 2(10): 1419–22. 10.1021/ol0057481 10814462

[25] Sheng SR, Xu L, Zhang XL, Liu XL, Wei MH. Solid-Phase Synthesis of Substituted Butenolides and Butyrolactones Using a Traceless Sulfone Linker. J Comb Chem. 2006; 8(6): 805–7. 10.1021/cc060104r 17096567

[26] Wang CL, Sheng SR, Cheng X, Cai MZ. Solid-phase organic synthesis of 5-iodomethyl-dihydrofuran-2-ones with recyclable polymer-supported selenium bromide. Synth Commun. 2012; 42(3): 320–7.

[27] Krchnak V, Moellmann U, Dahse HM, Miller MJ. Solid-Supported Nitroso Hetero Diels-Alder Reactions. 2. Arylnitroso Dienophiles: Scope and Limitations. J Comb Chem. 2008; 10(1): 104–11. 10.1021/cc7001414 18062671

[28] Kresze G, Firl J, Braun H. Addition reactions of the nitroso group. XI. Steric effect on the orientation in diene synthesis with nitroso aromatics. Tetrahedron. 1969; 25(18): 4481–6.

[29] Harrison A, Melchionna M, Franco P, Hlavac J. Solid-phase synthesis and analysis of 3,6-dihydro-2H-1,2-oxazines in their stereo- and regioisomer mixtures. New J Chem. 2014; 38(11): 5491–5499.

