# Supplementary figures and images for: Solid-Phase Synthesis of ɤ-Lactone and 1,2-Oxazine Derivatives and Their Efficient Chiral Analysis
Source: PLoS One. 2016 Nov 28;11(11):e0166558. doi: 10.1371/journal.pone.0166558 (PMC5125624; doi:10.1371/journal.pone.0166558)

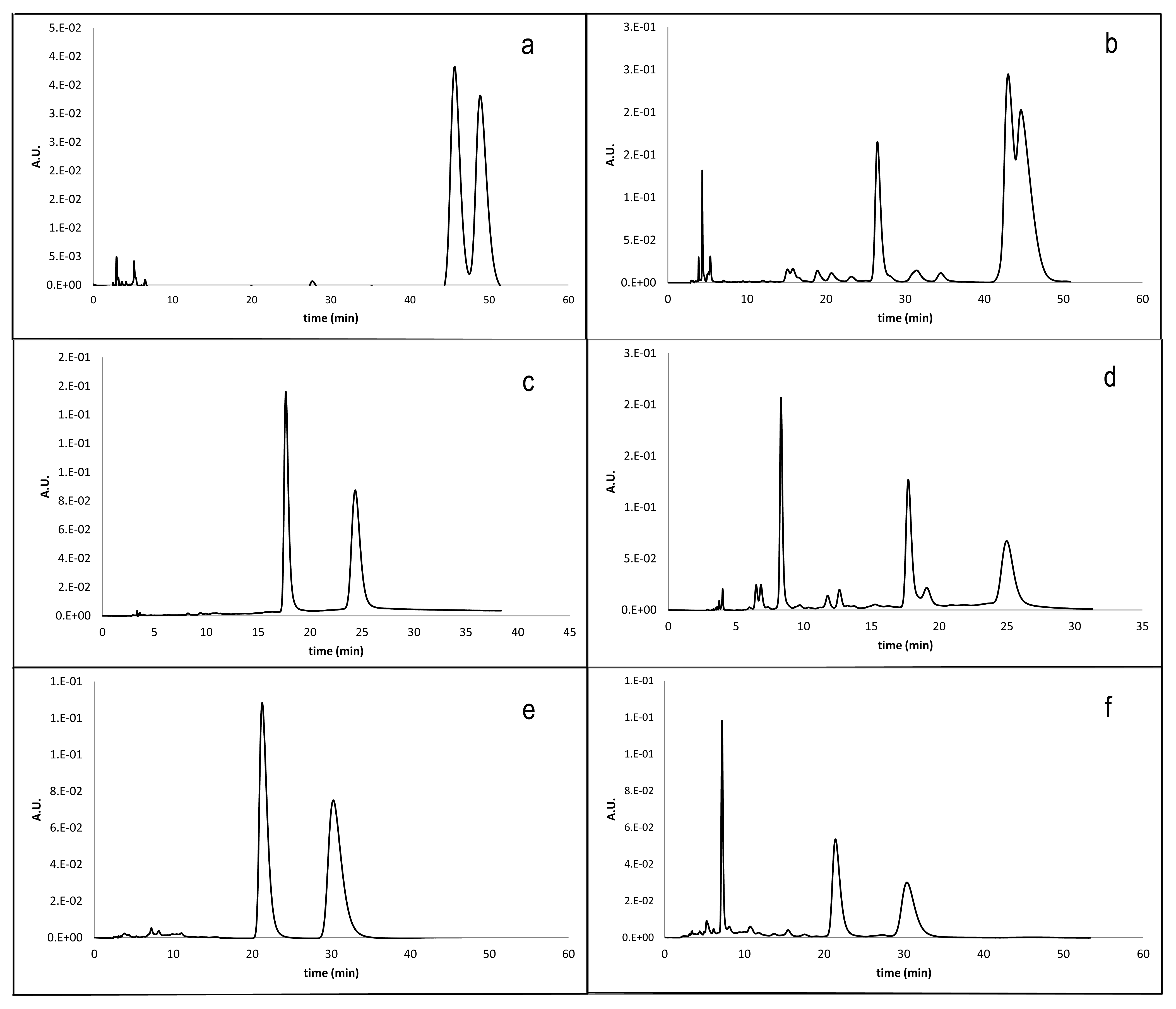

Supplement: S1 Fig — a) 12(3,1,1) pure compound; b) 12(3,1,1) crude mixture; c) 11(3,1,1) pure compound; d) 11(3,1,1) crude mixture; e) 11(4,1,1) pure compound; f) 11(4,1,1) crude mixture. (TIF) [file pone.0166558.s001.tif]

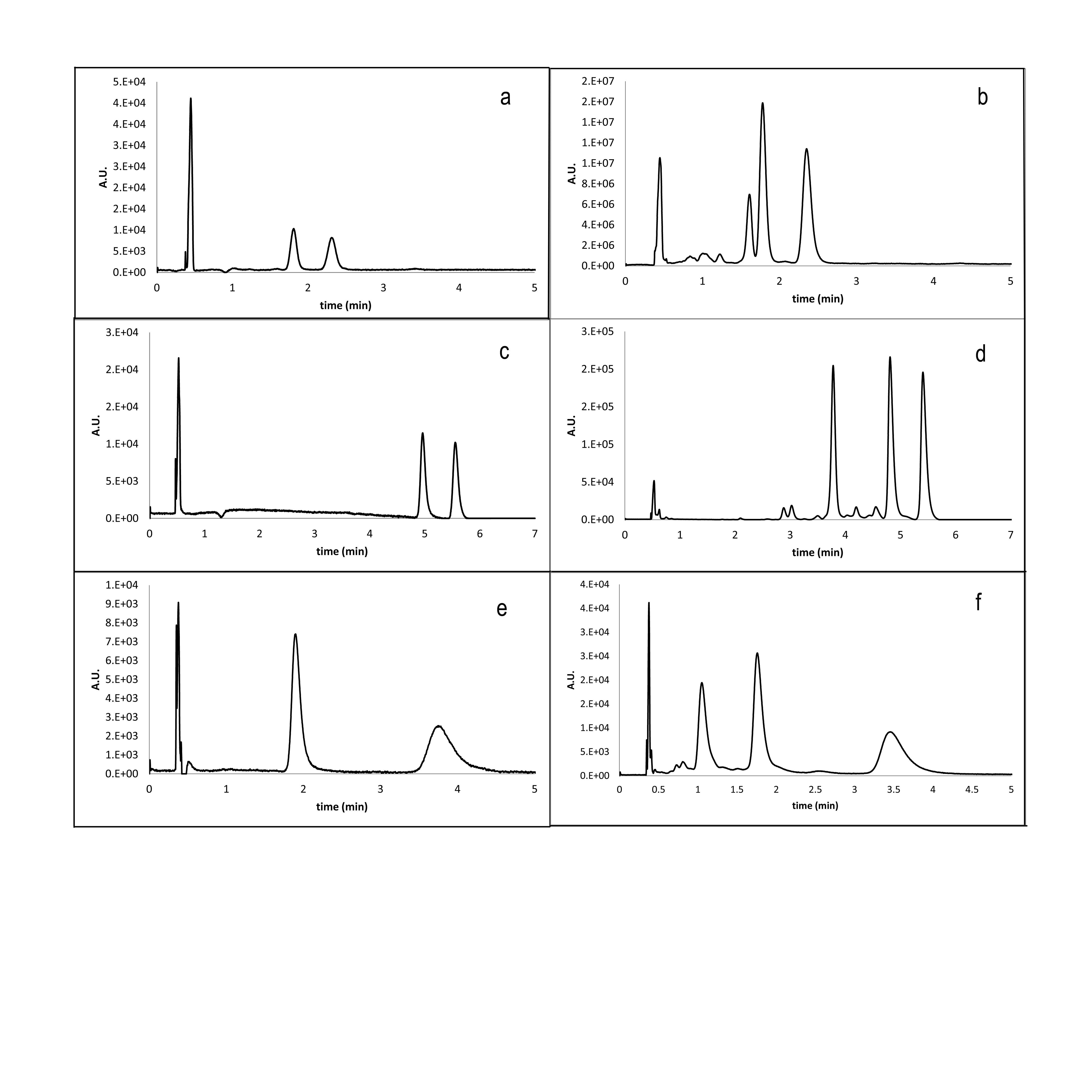

Supplement: S2 Fig — a) 12(3,1,1) pure compound; b) 12(3,1,1) crude mixture; c) 11(3,1,1) pure compound; d) 11(3,1,1) crude mixture; e) 11(4,1,1) pure compound; f) 11(4,1,1) crude mixture. (TIF) [file pone.0166558.s002.tif]

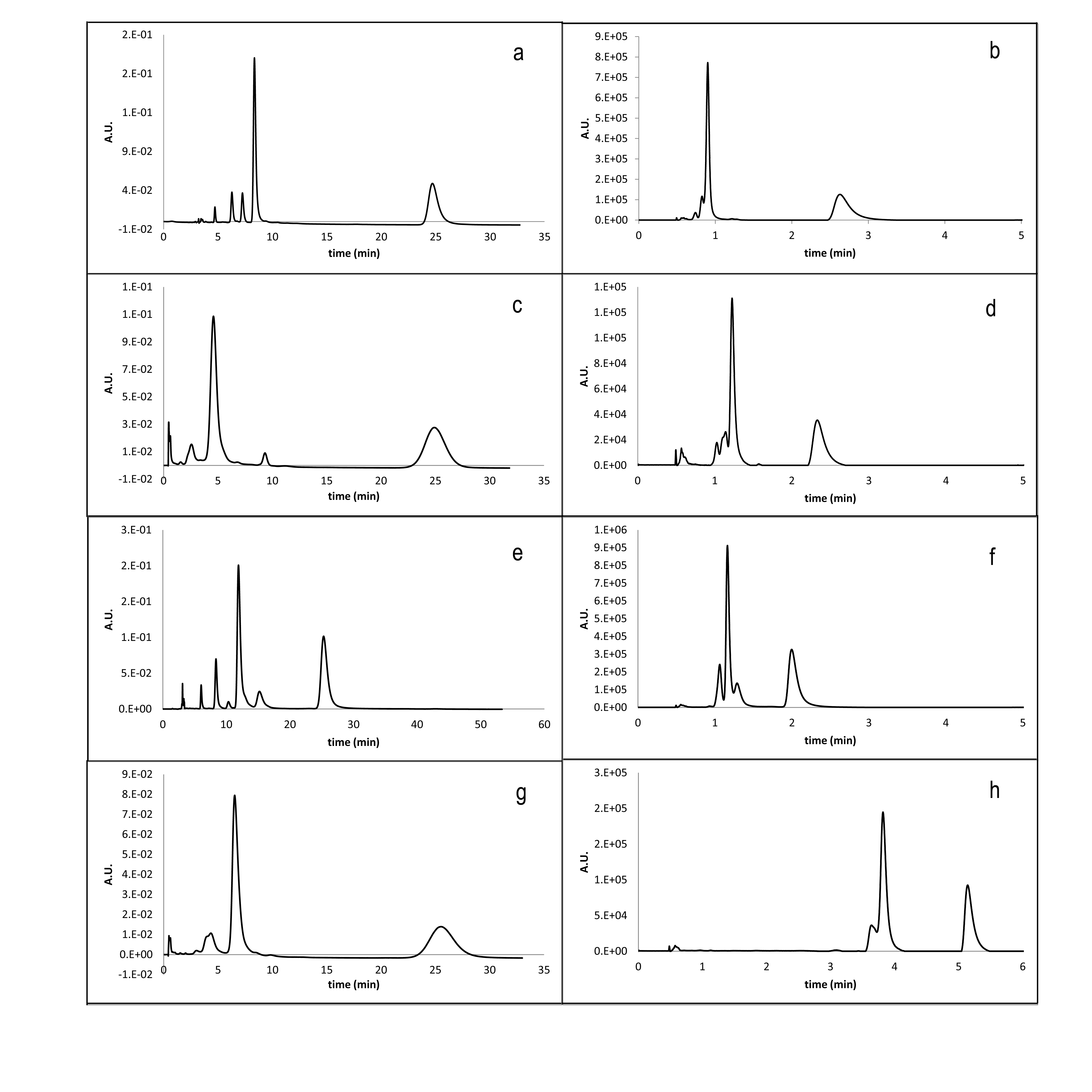

Supplement: S3 Fig — a) 8(2,1) HPLC; b) 8(2,1) SFC; c) 8(4,1) HPLC; d) 8(4,1) SFC; e) 8(3,1) HPLC; f) 8(3,1) SFC; g) 8(1,1) HPLC; h) 8(1,1) SFC. (TIF) [file pone.0166558.s003.tif]

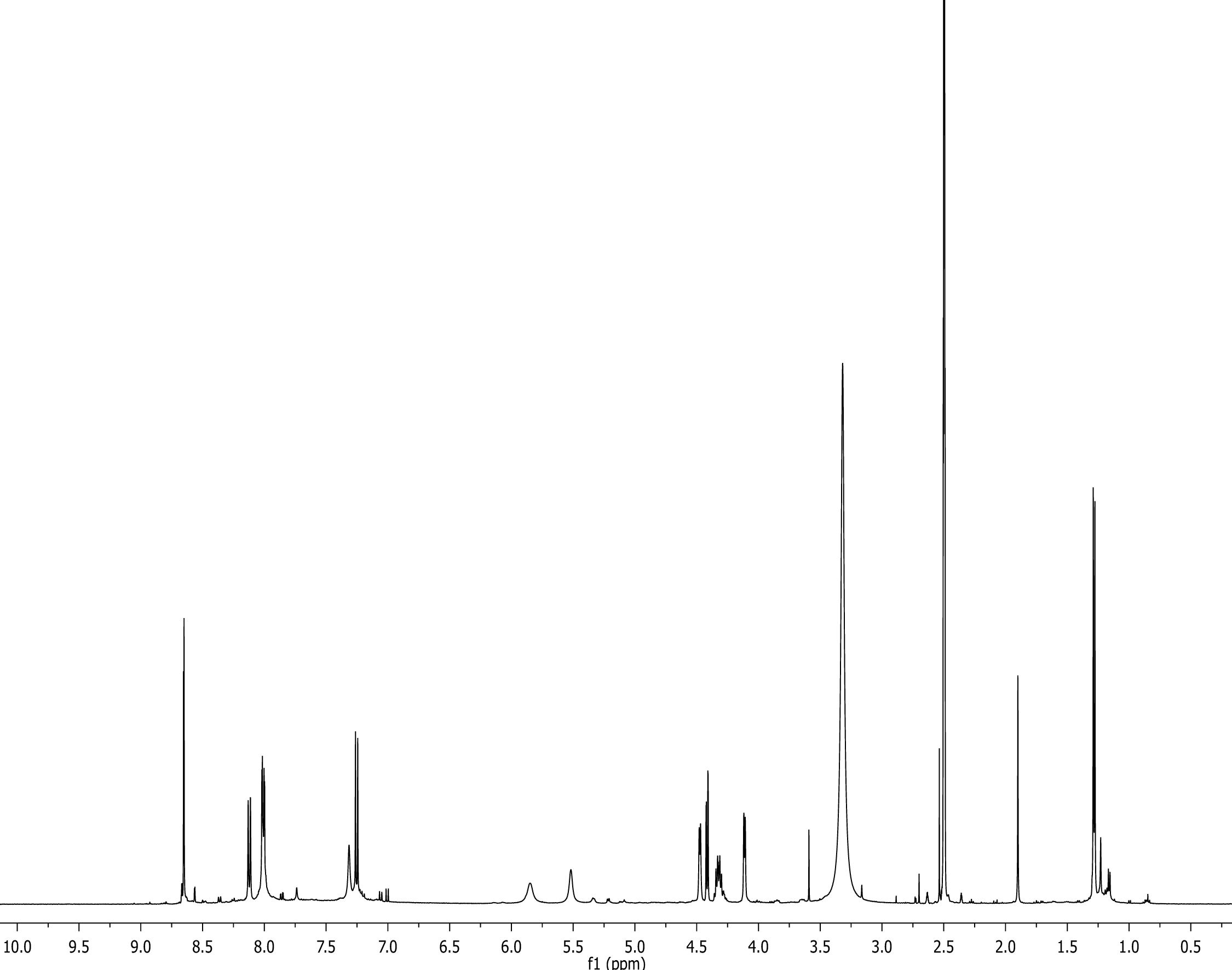

Supplement: S4 Fig — (TIF) [file pone.0166558.s004.tif]

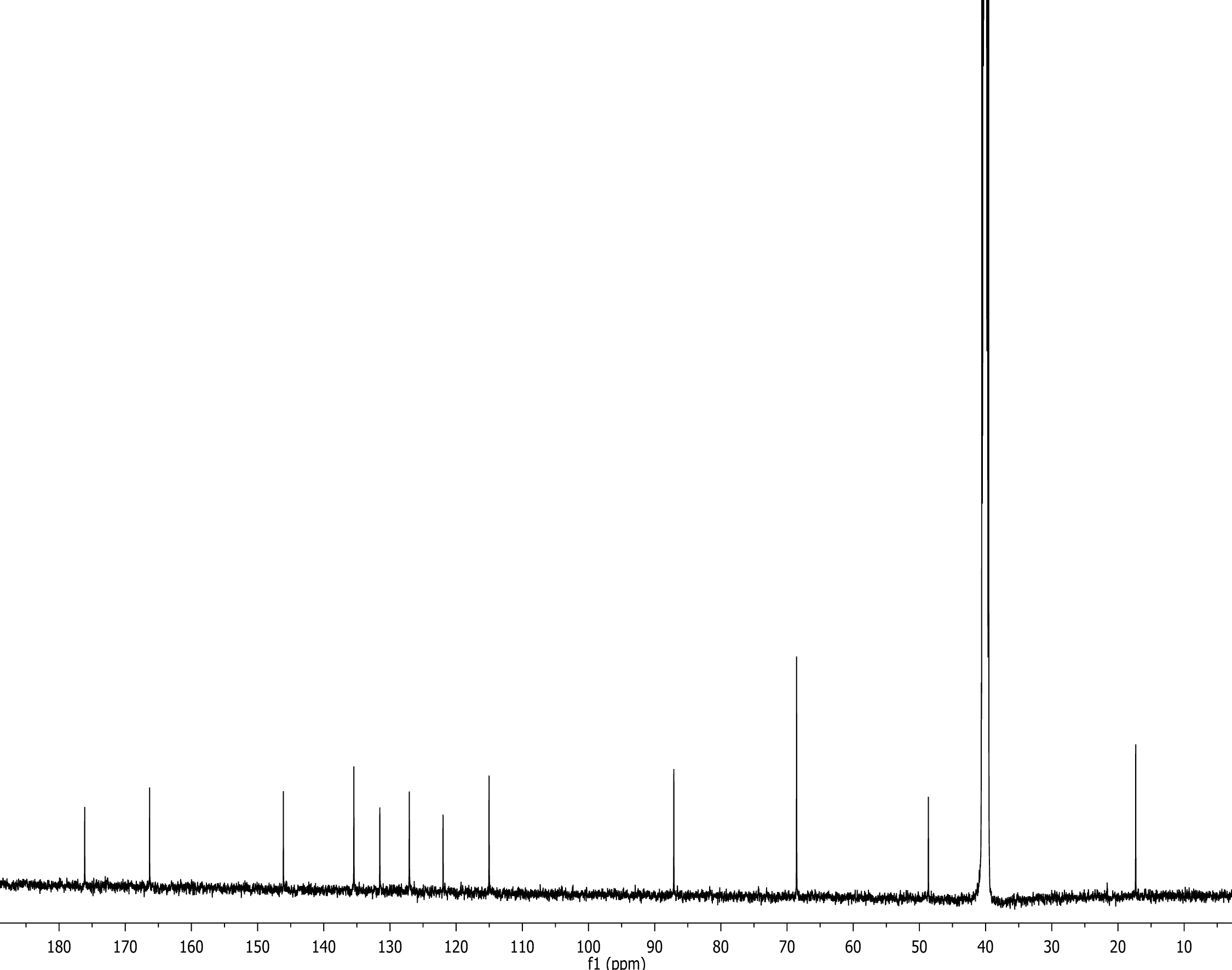

Supplement: S5 Fig — (TIF) [file pone.0166558.s005.tif]

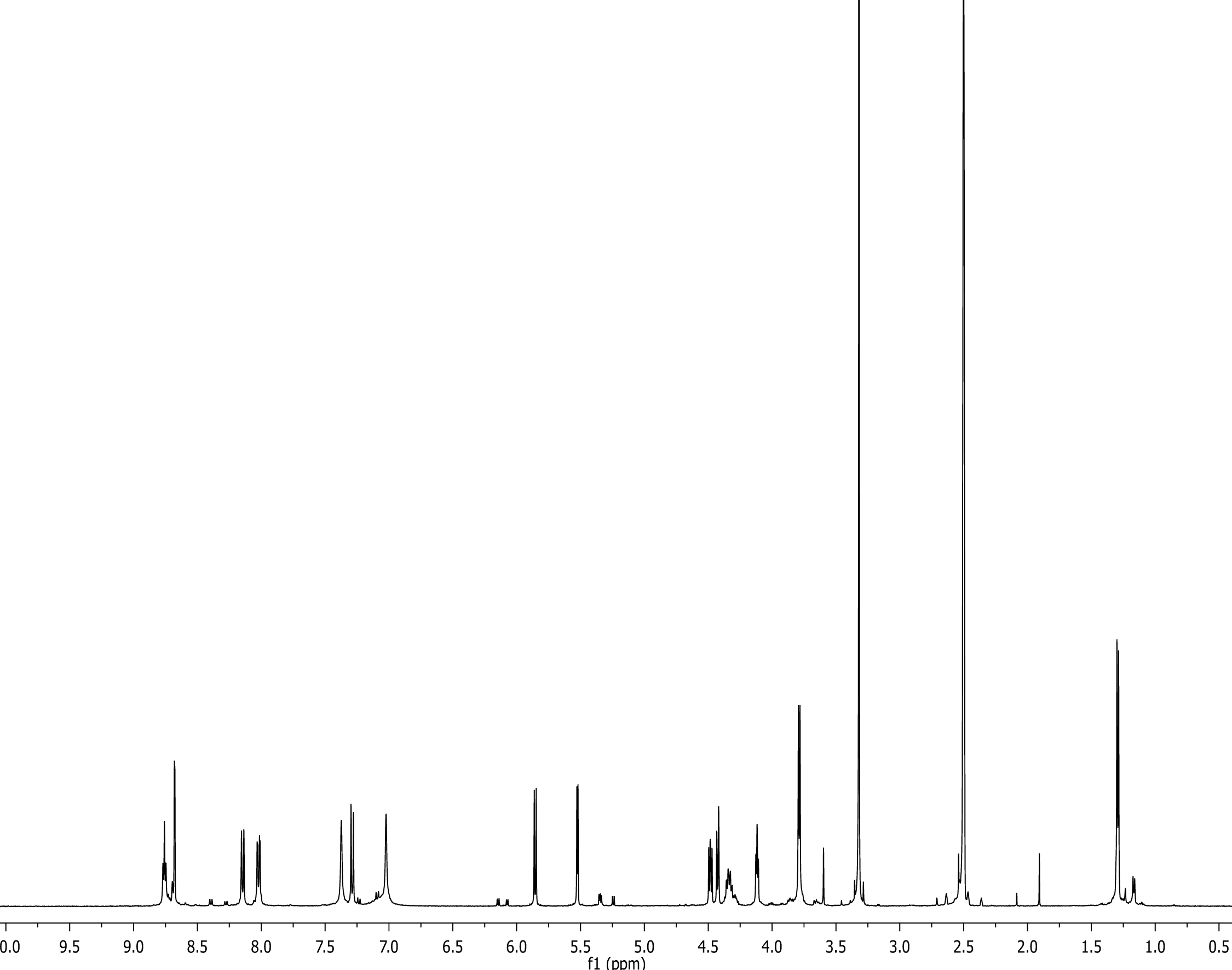

Supplement: S6 Fig — (TIF) [file pone.0166558.s006.tif]

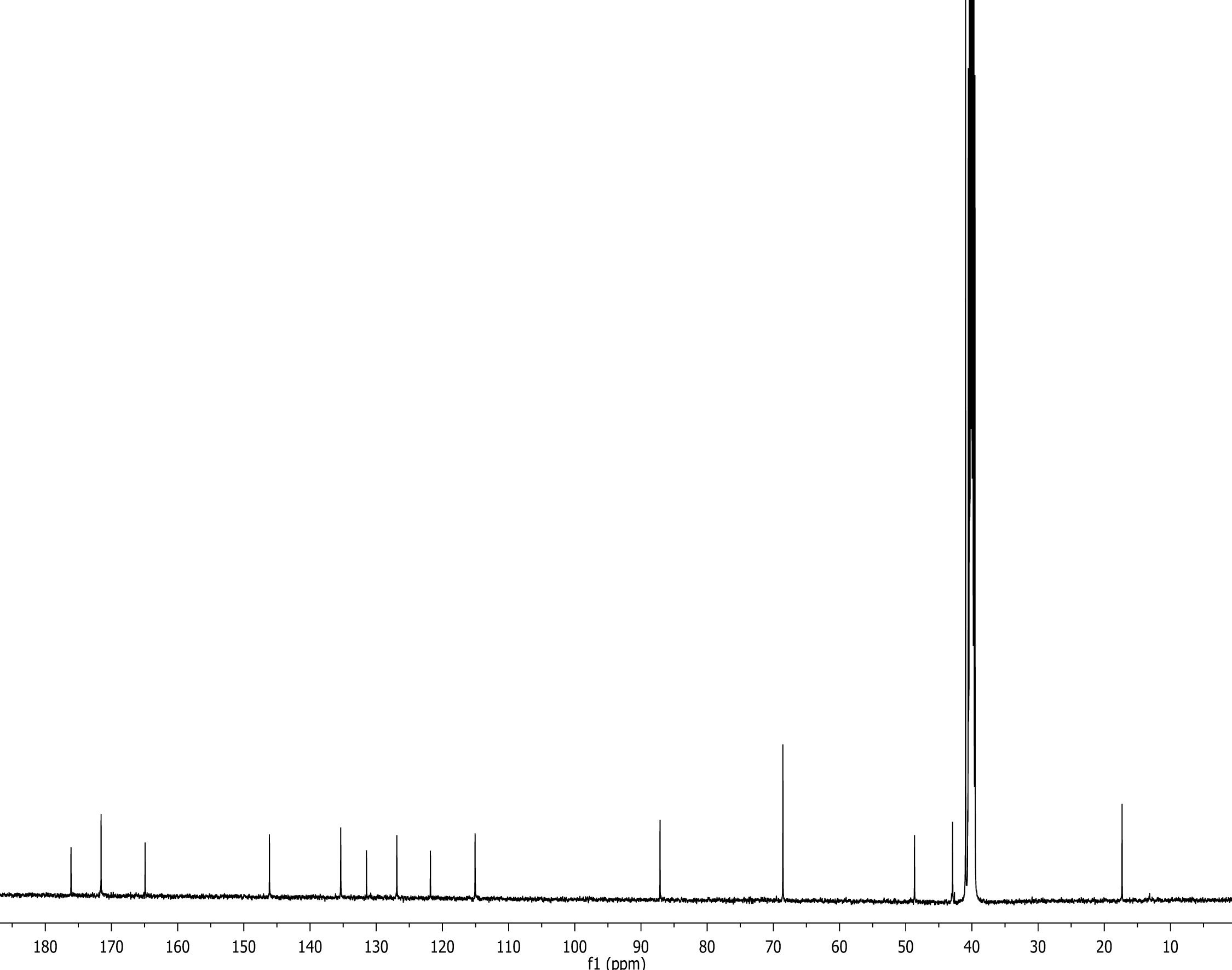

Supplement: S7 Fig — (TIF) [file pone.0166558.s007.tif]

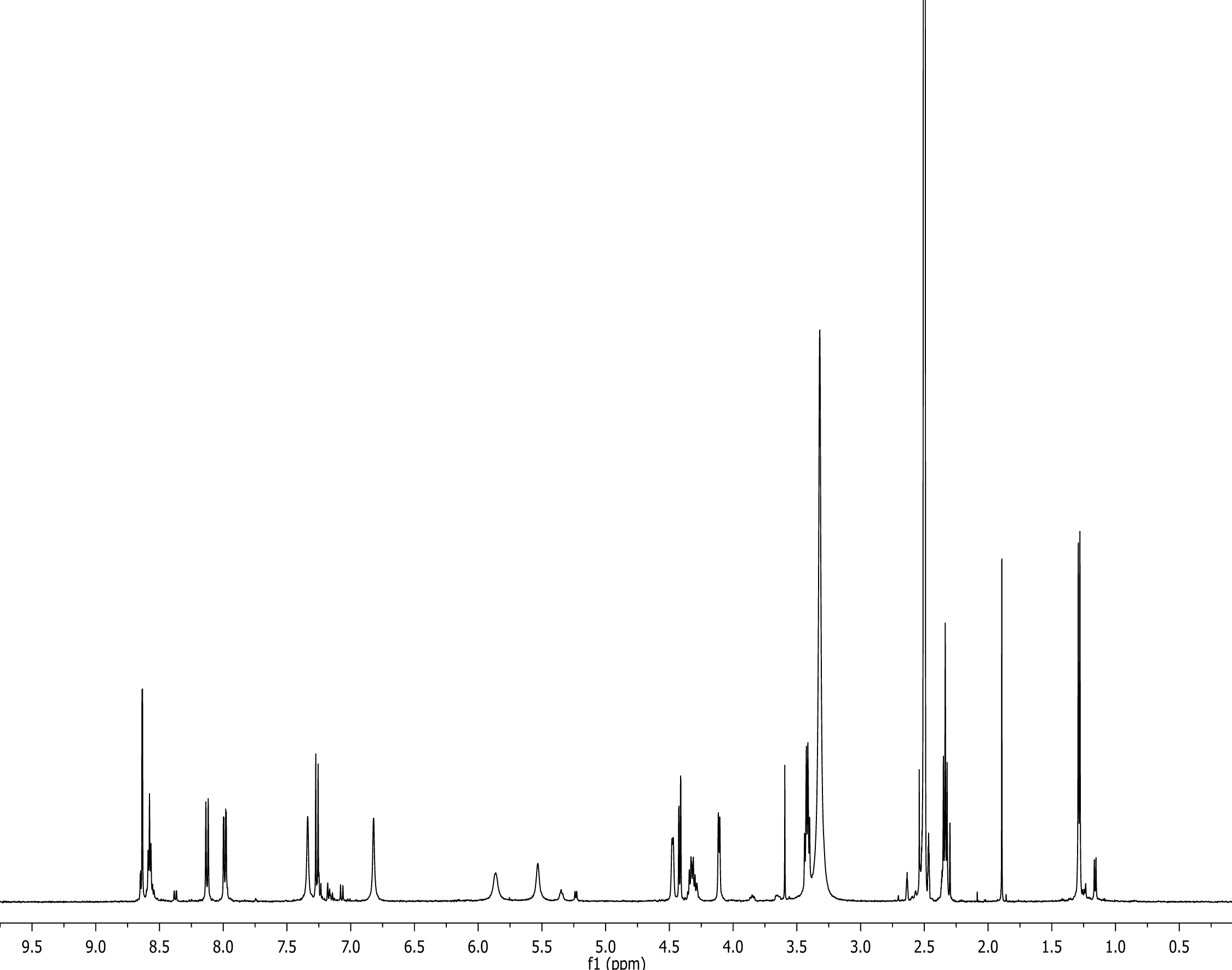

Supplement: S8 Fig — (TIF) [file pone.0166558.s008.tif]

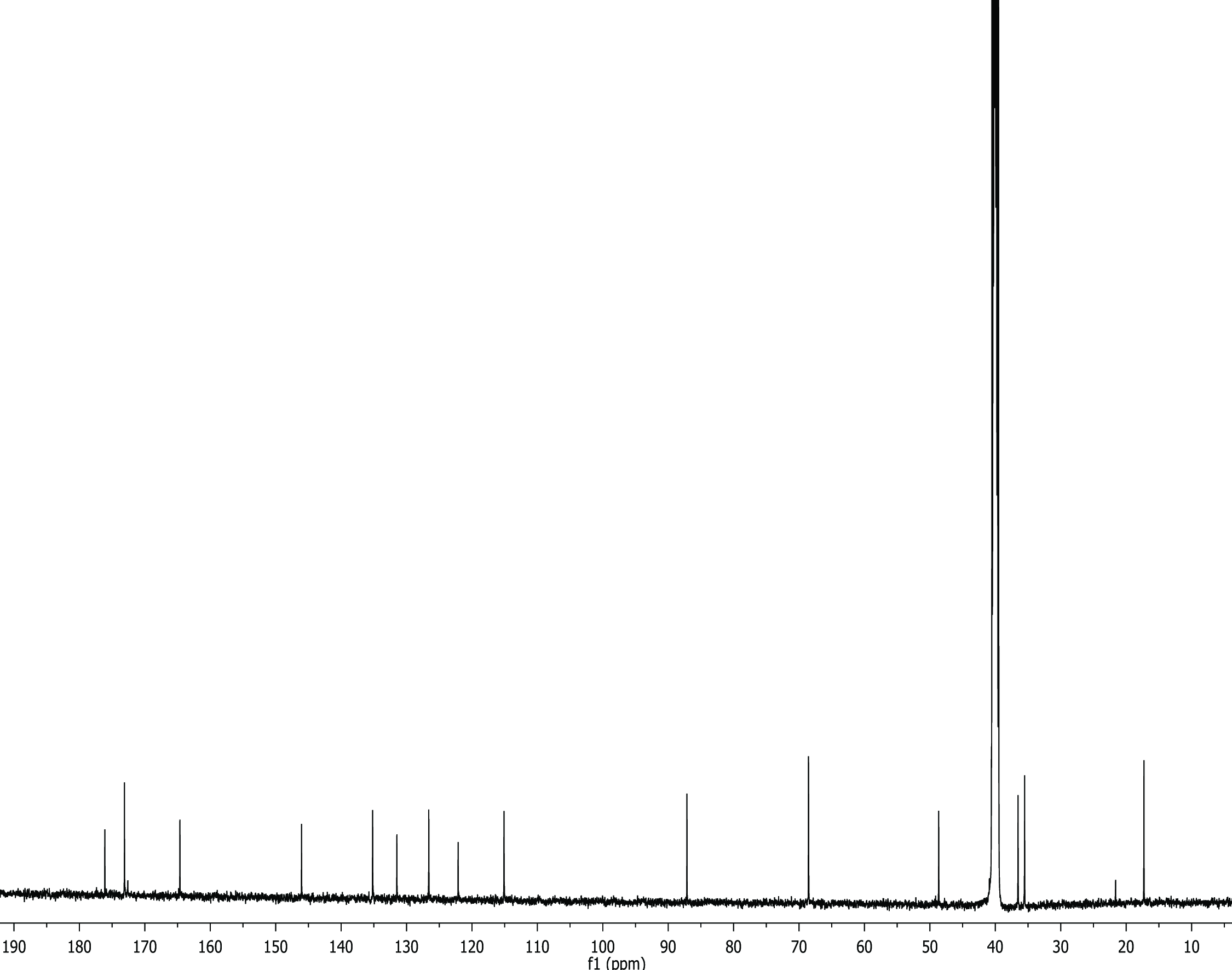

Supplement: S9 Fig — (TIF) [file pone.0166558.s009.tif]

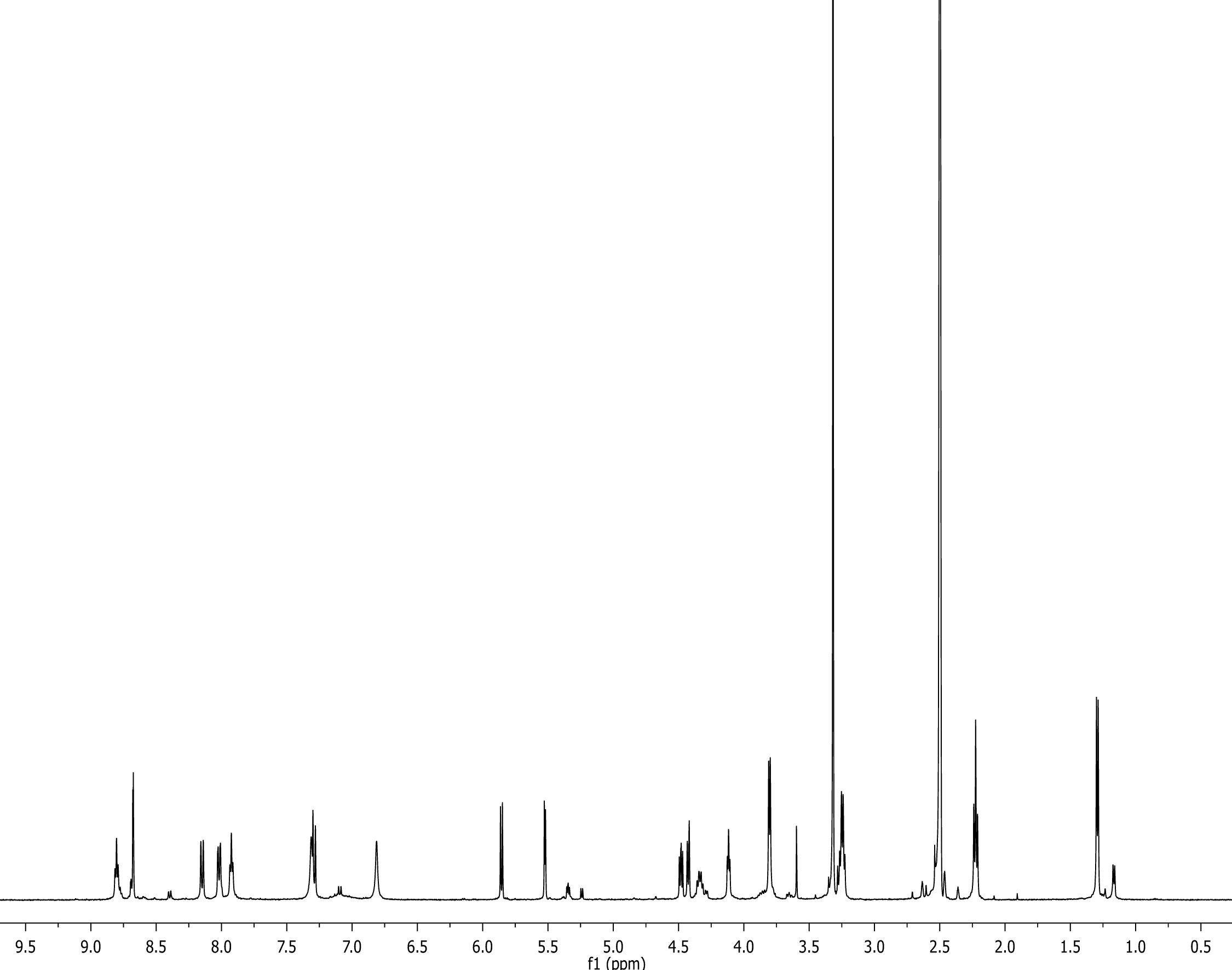

Supplement: S10 Fig — (TIF) [file pone.0166558.s010.tif]

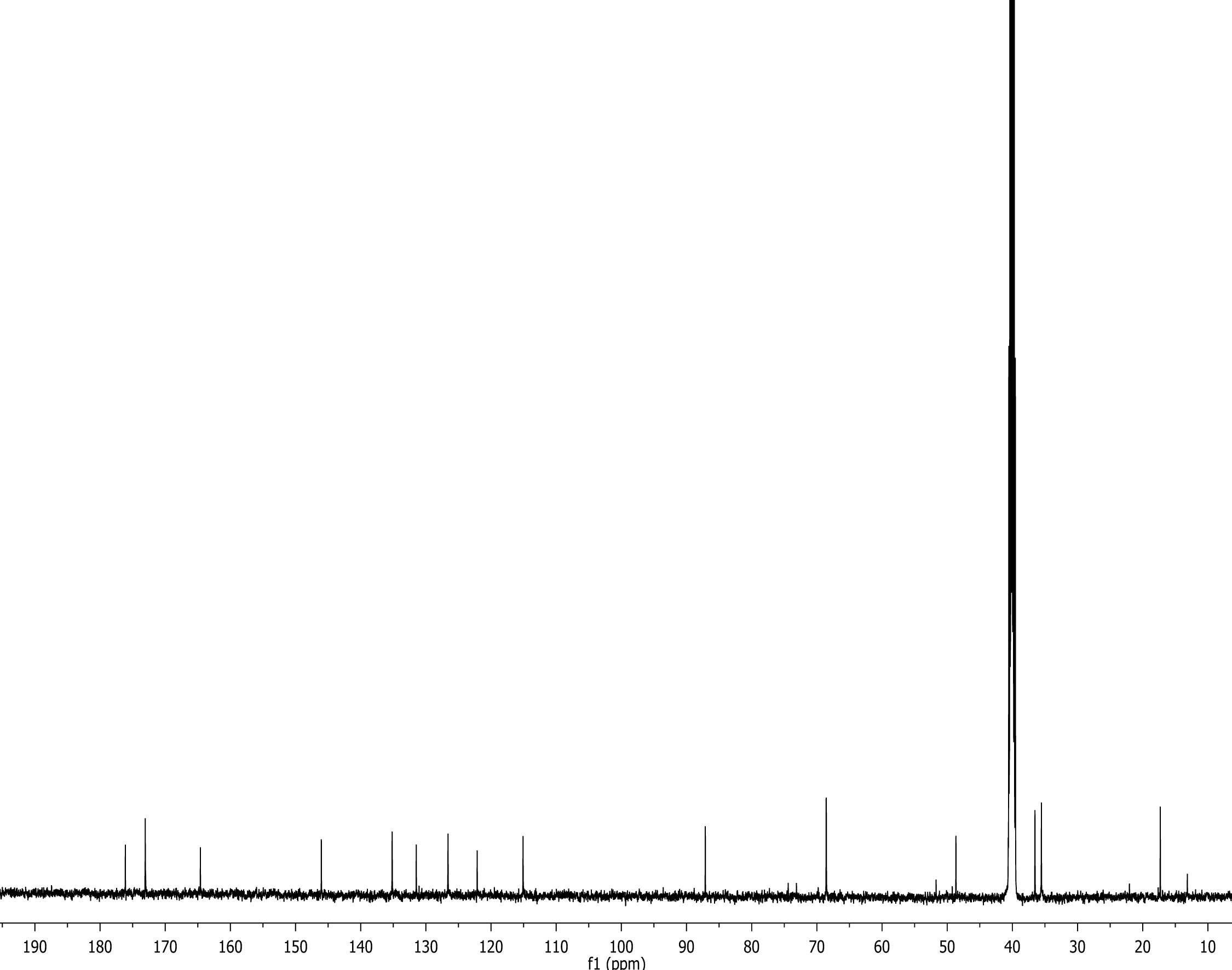

Supplement: S11 Fig — (TIF) [file pone.0166558.s011.tif]

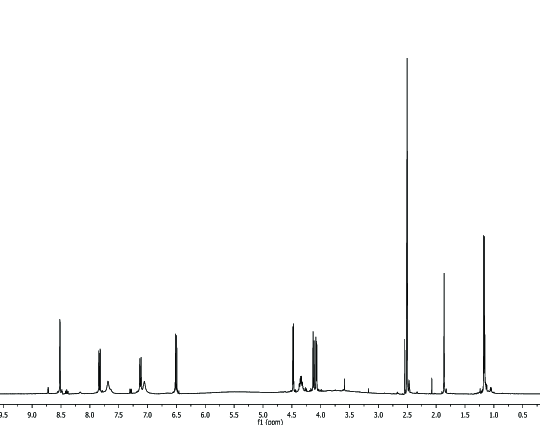

Supplement: S12 Fig — (TIF) [file pone.0166558.s012.tif]

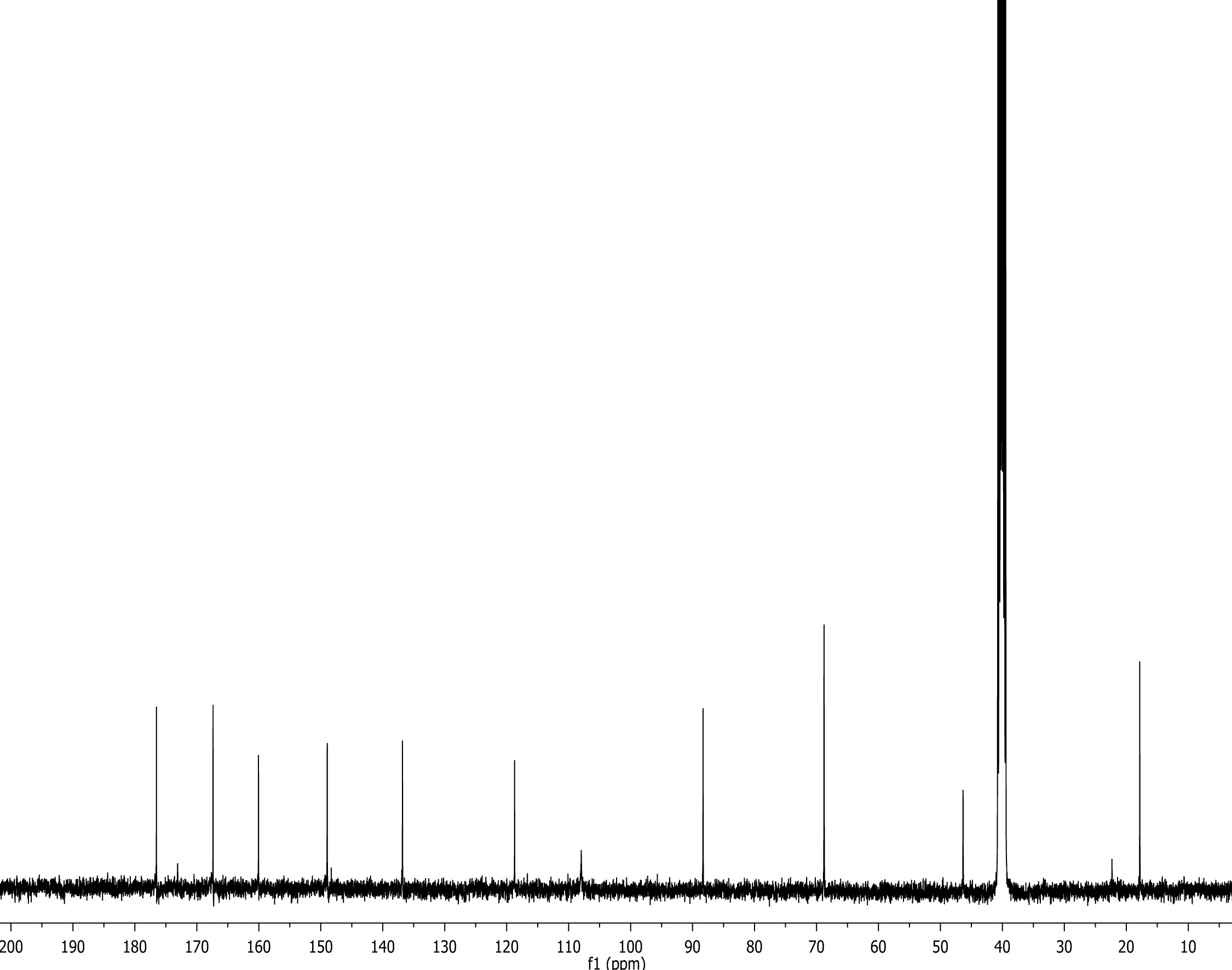

Supplement: S13 Fig — (TIF) [file pone.0166558.s013.tif]

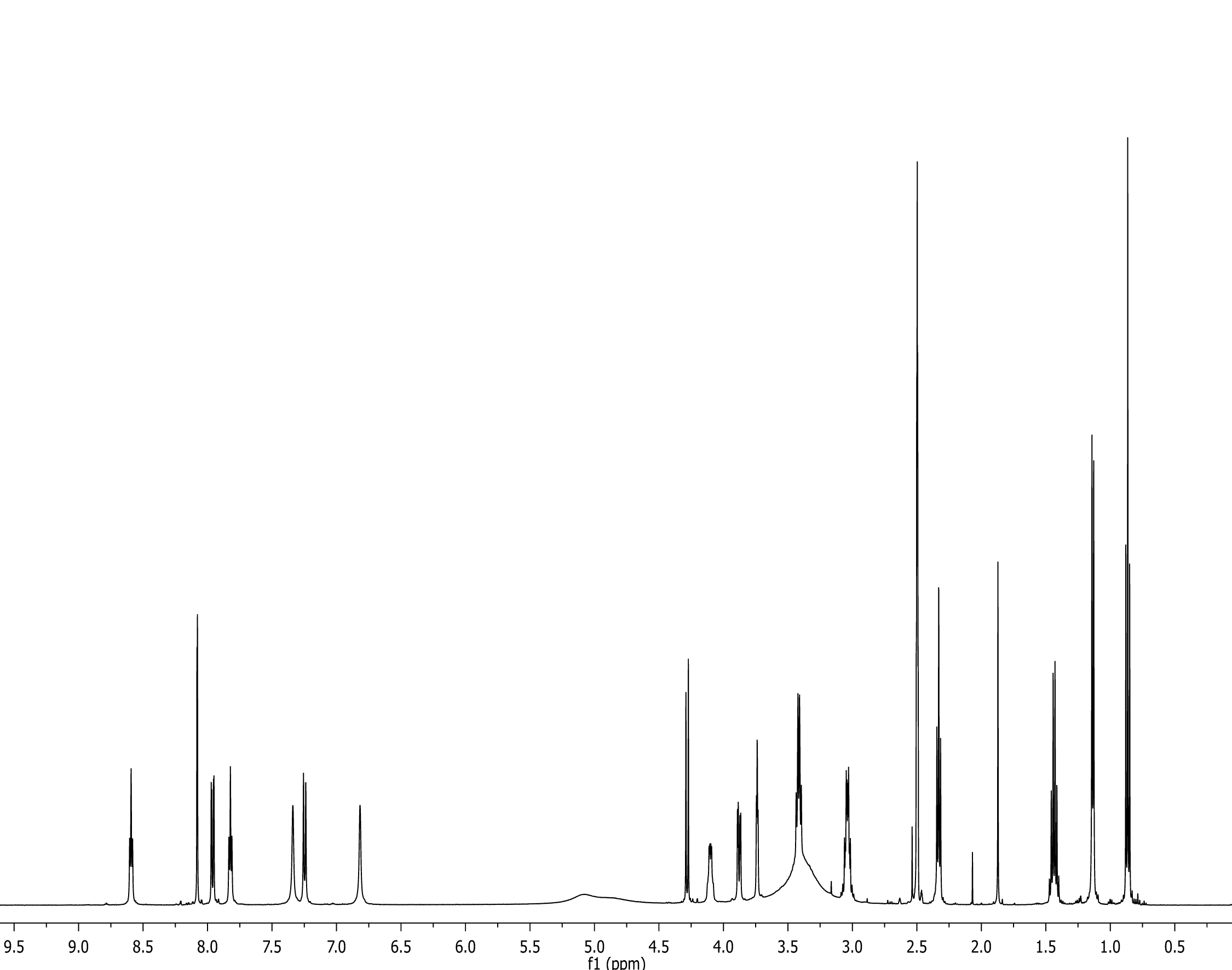

Supplement: S14 Fig — (TIF) [file pone.0166558.s014.tif]

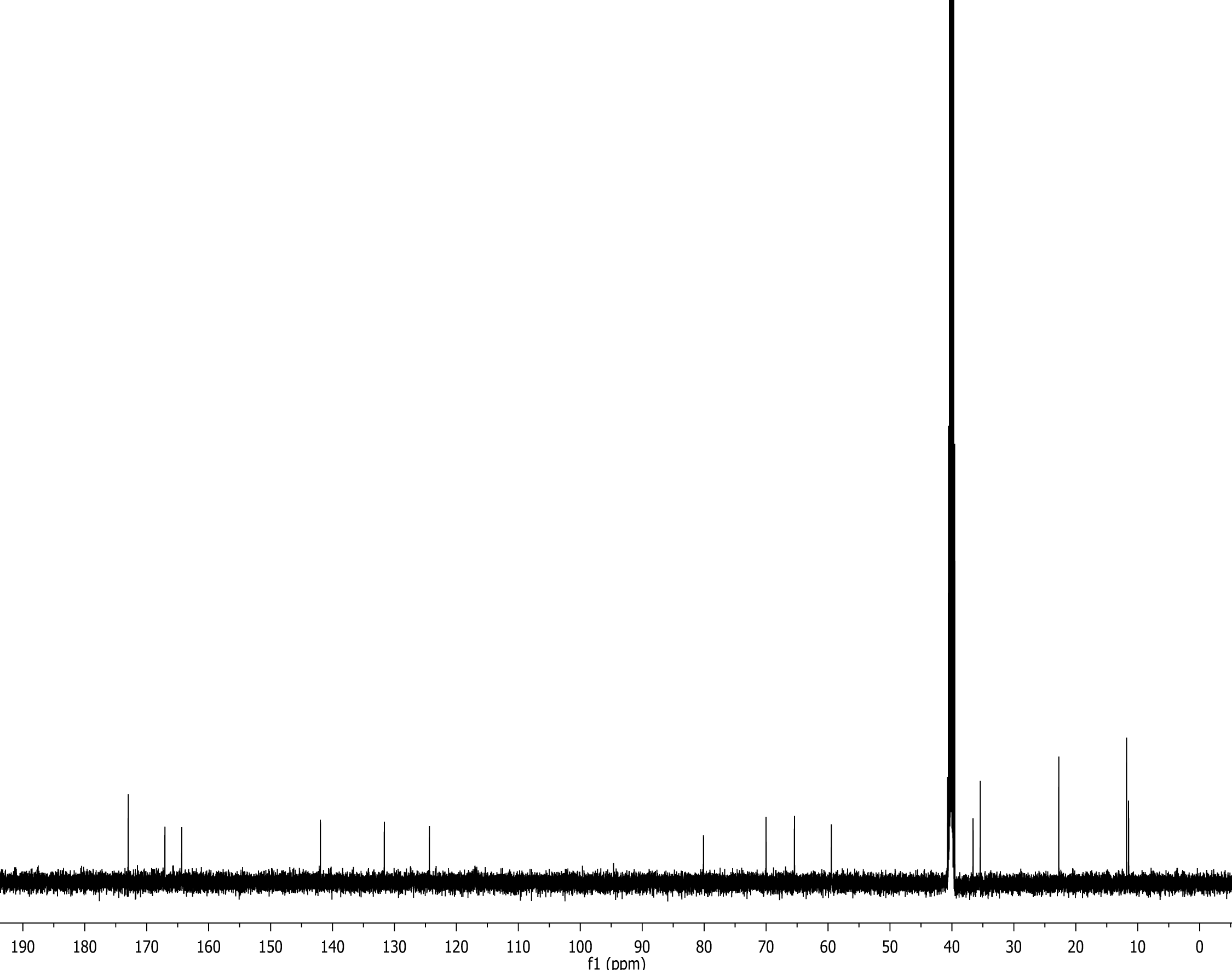

Supplement: S15 Fig — (TIF) [file pone.0166558.s015.tif]

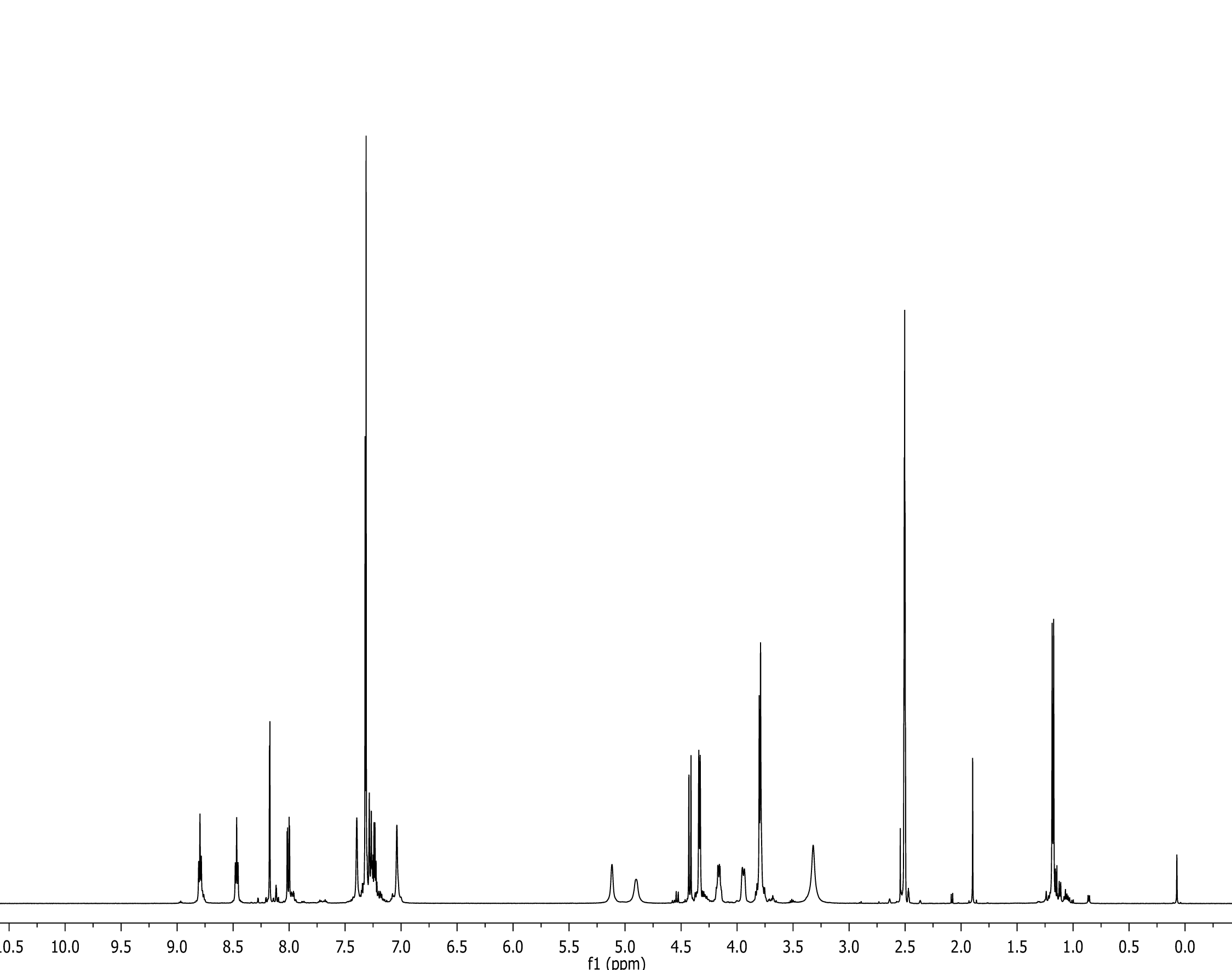

Supplement: S16 Fig — (TIF) [file pone.0166558.s016.tif]

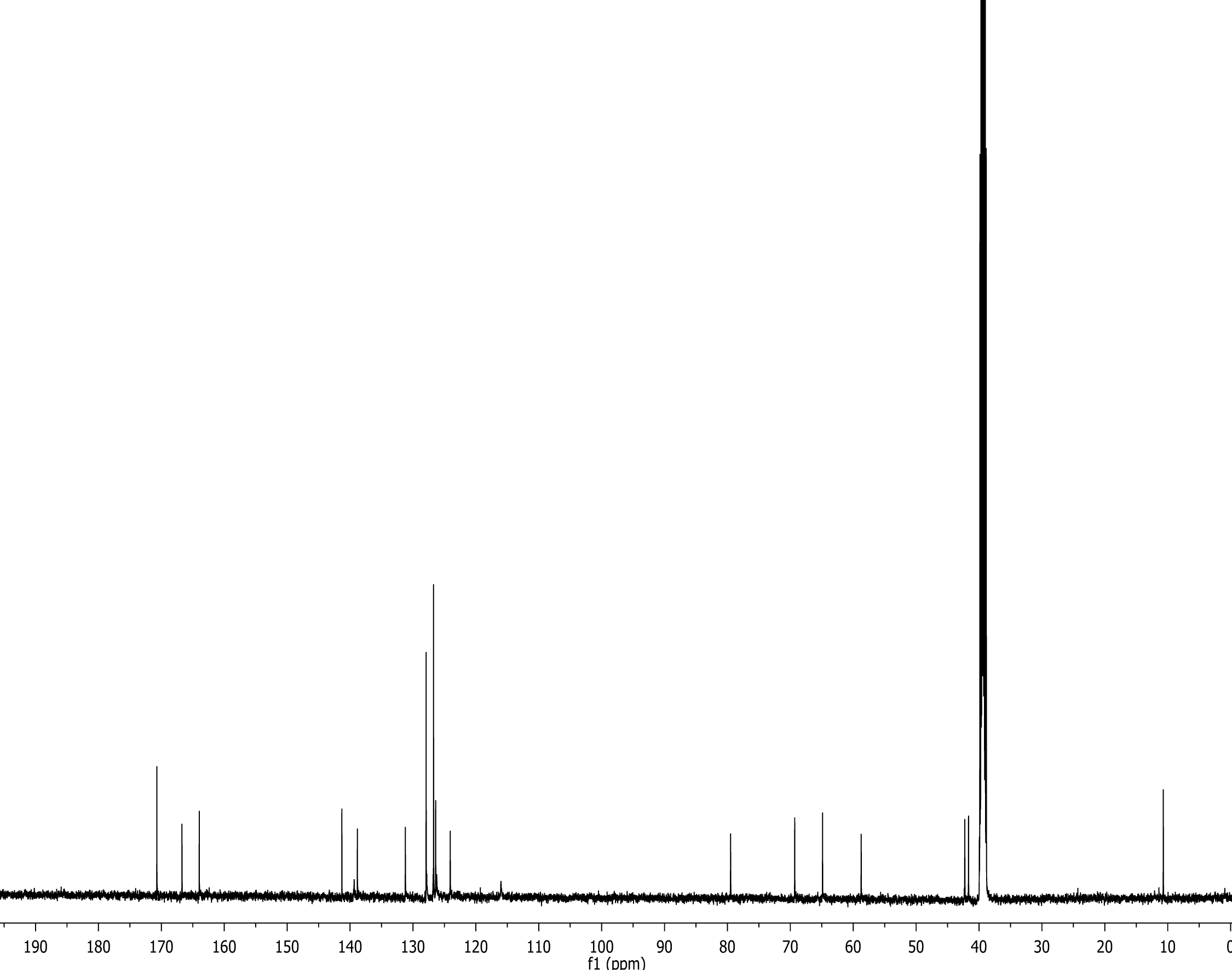

Supplement: S17 Fig — (TIF) [file pone.0166558.s017.tif]

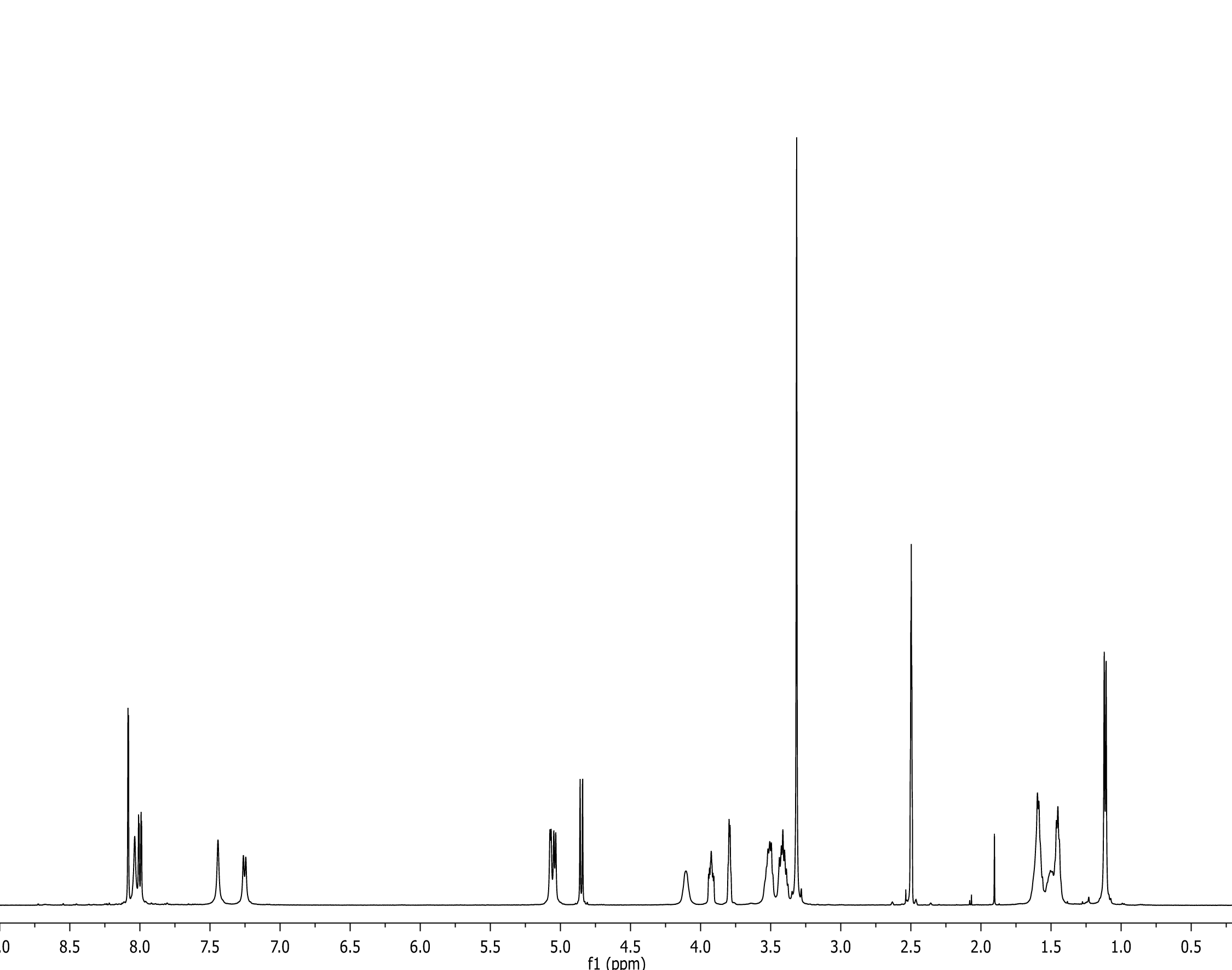

Supplement: S18 Fig — (TIF) [file pone.0166558.s018.tif]

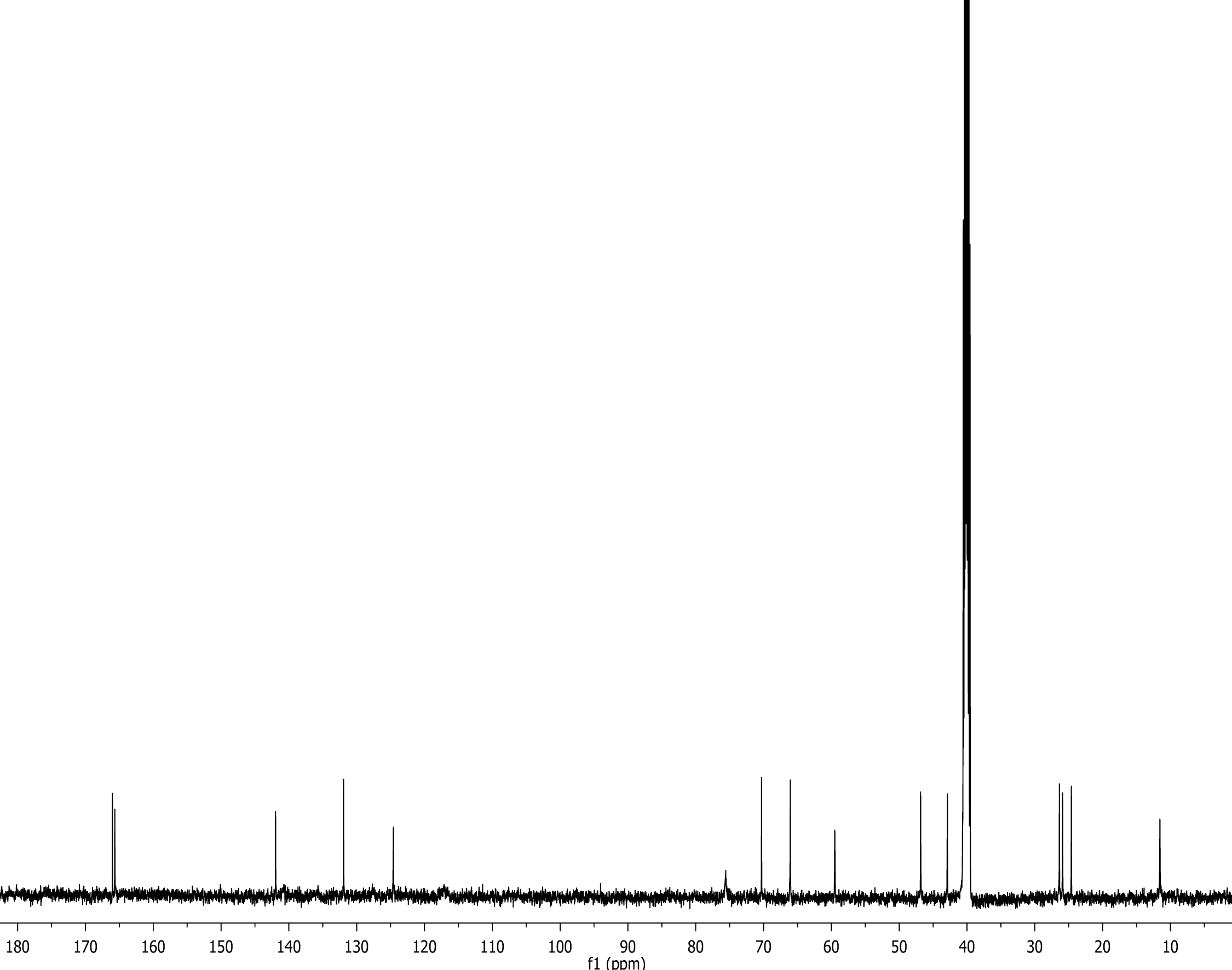

Supplement: S19 Fig — (TIF) [file pone.0166558.s019.tif]

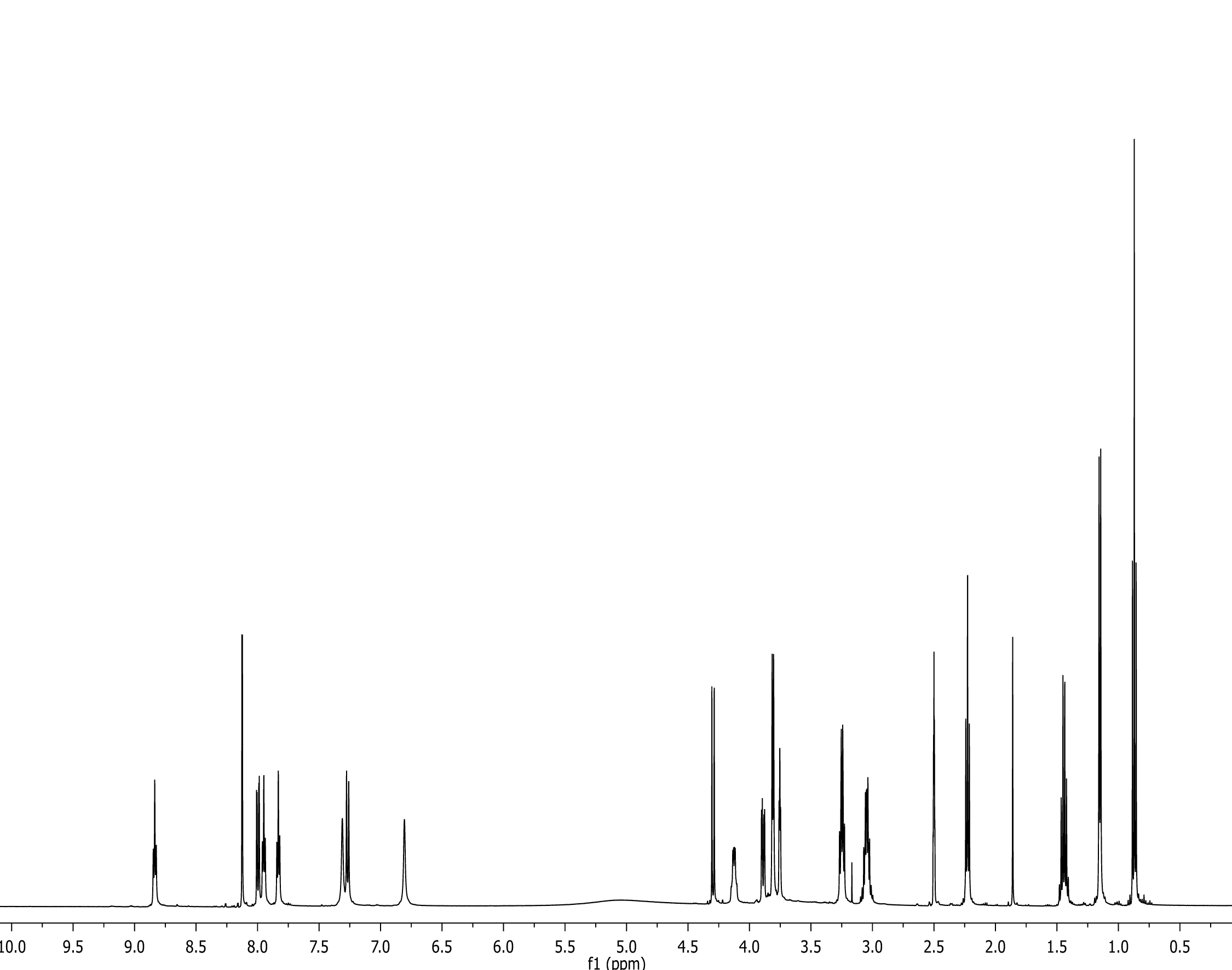

Supplement: S20 Fig — (TIF) [file pone.0166558.s020.tif]

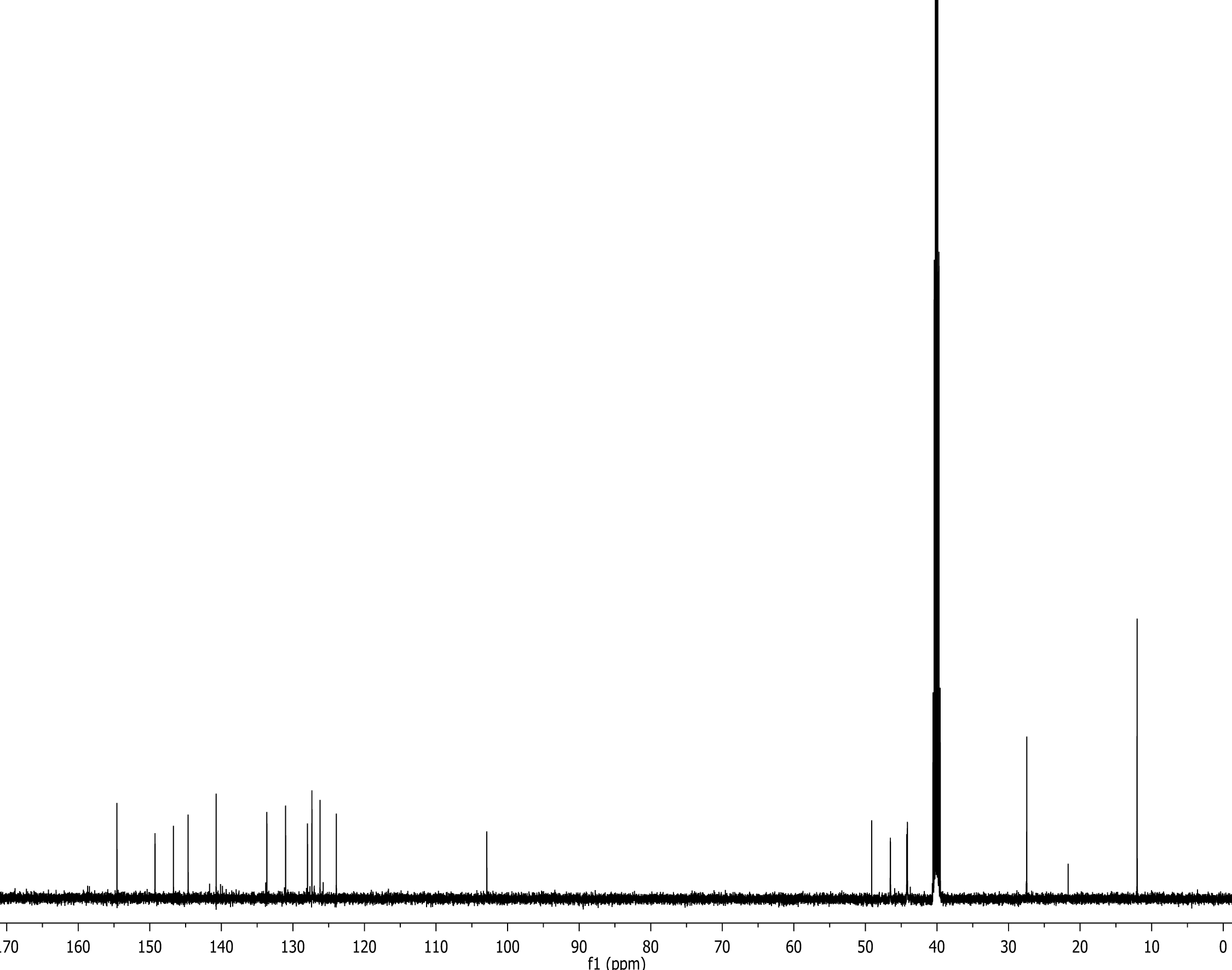

Supplement: S21 Fig — (TIF) [file pone.0166558.s021.tif]

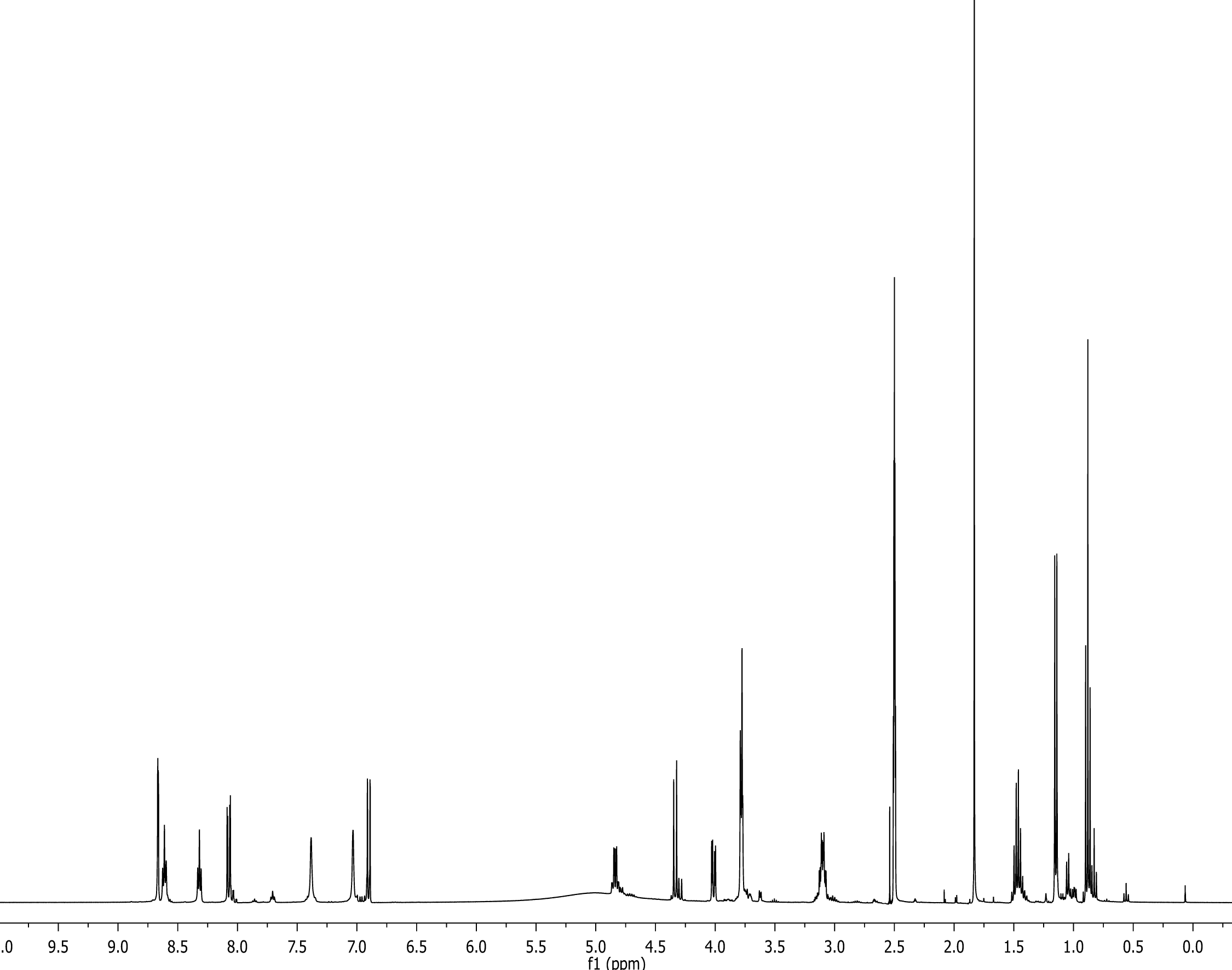

Supplement: S22 Fig — (TIF) [file pone.0166558.s022.tif]

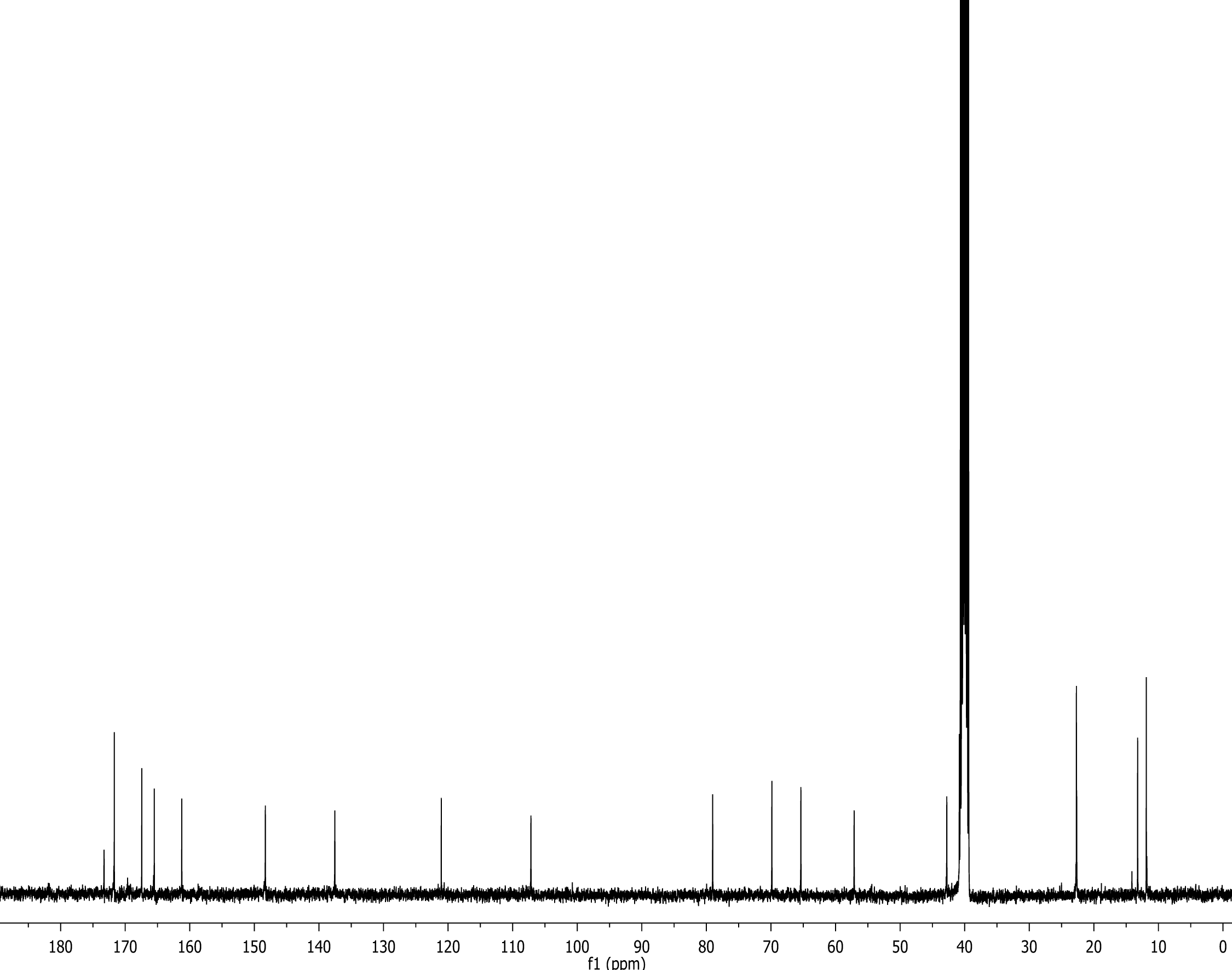

Supplement: S23 Fig — (TIF) [file pone.0166558.s023.tif]

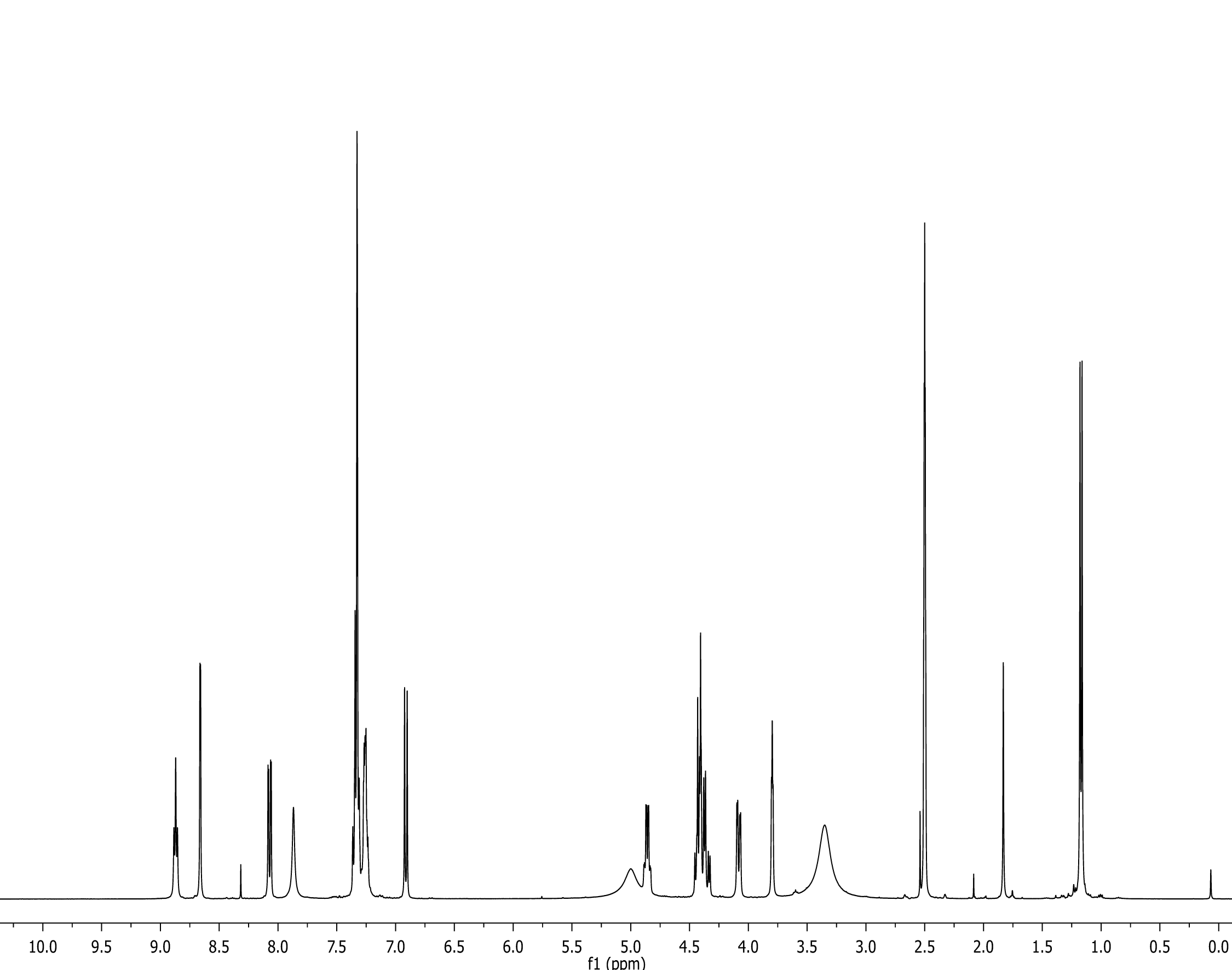

Supplement: S24 Fig — (TIF) [file pone.0166558.s024.tif]

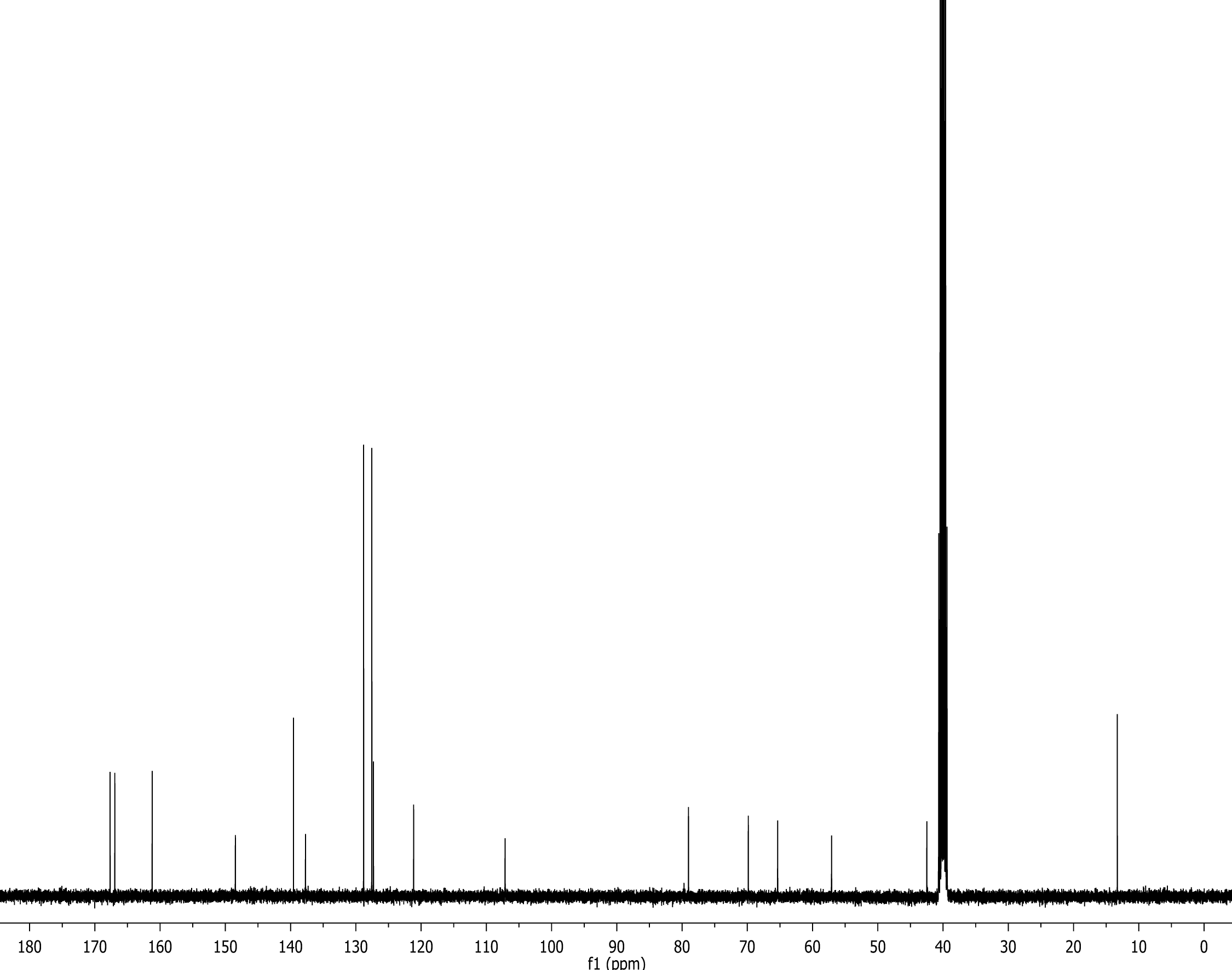

Supplement: S25 Fig — (TIF) [file pone.0166558.s025.tif]

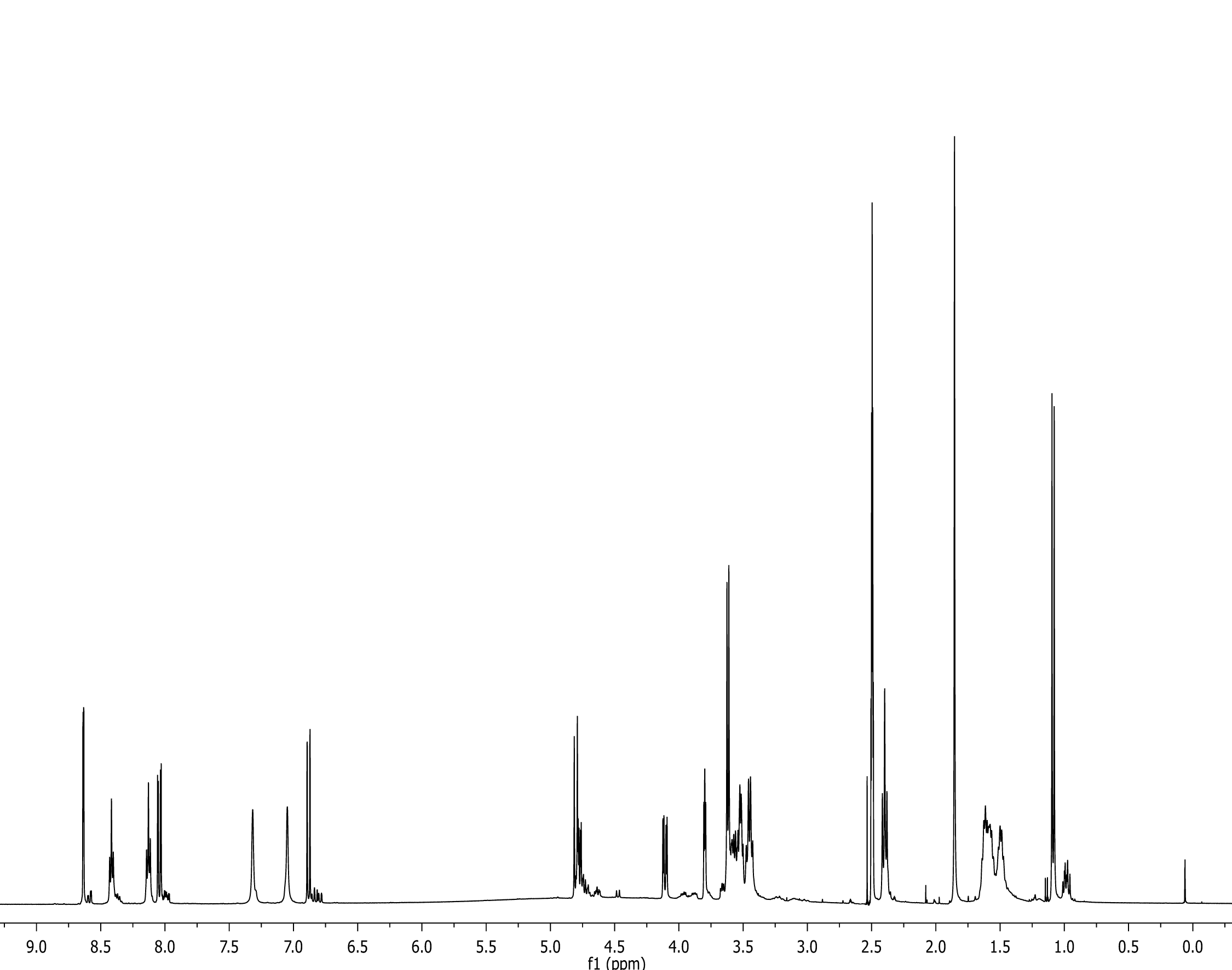

Supplement: S26 Fig — (TIF) [file pone.0166558.s026.tif]

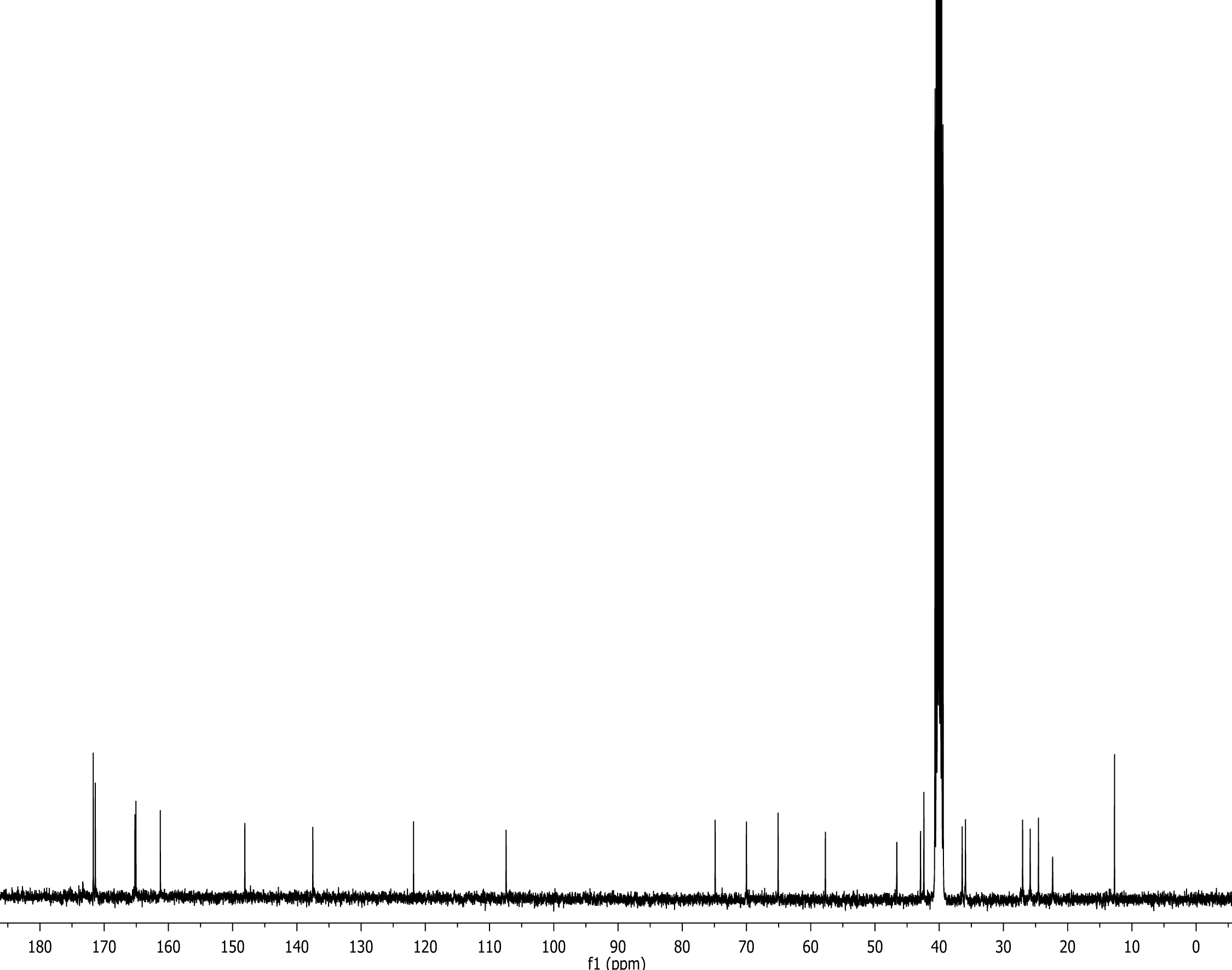

Supplement: S27 Fig — (TIF) [file pone.0166558.s027.tif]

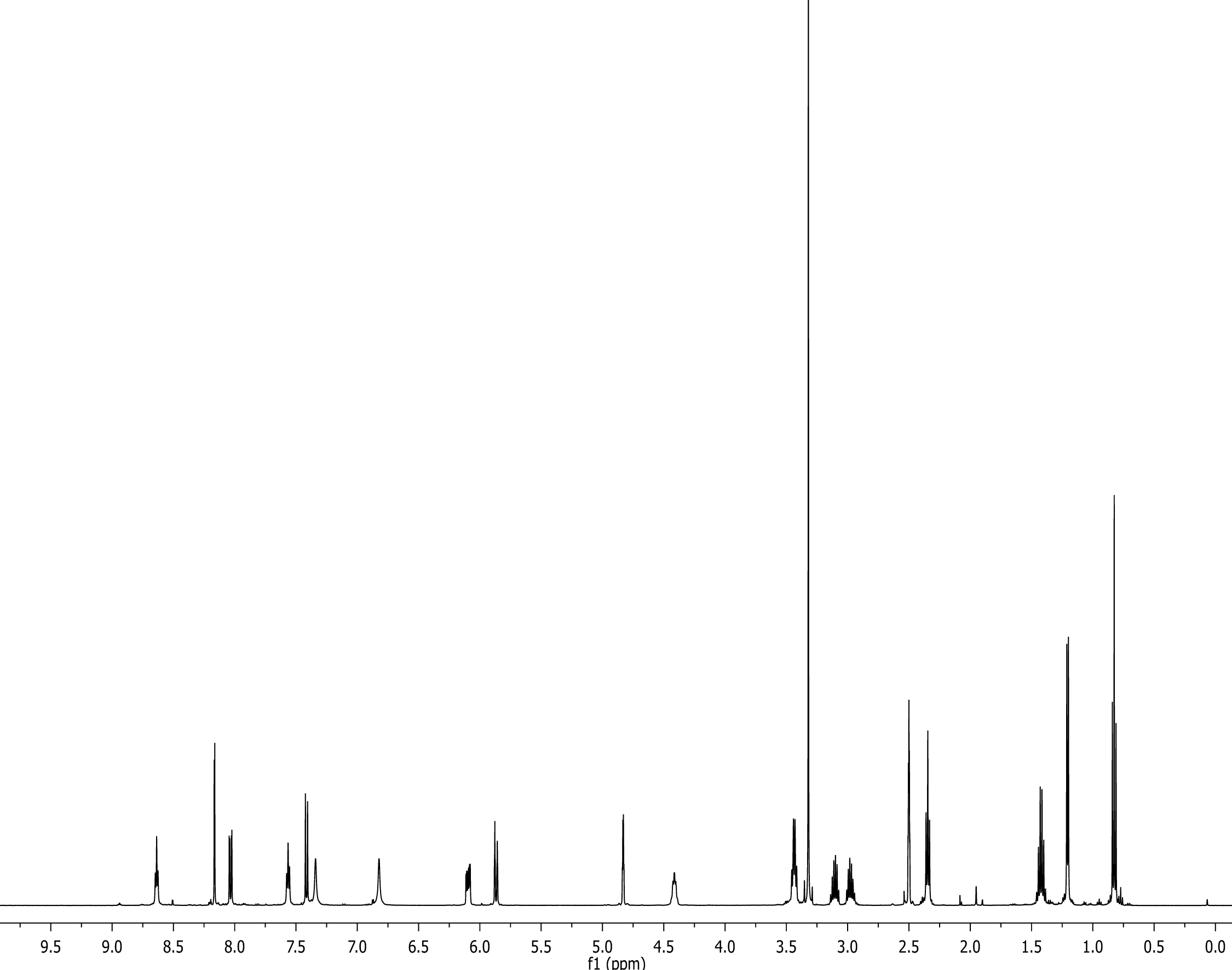

Supplement: S28 Fig — (TIF) [file pone.0166558.s028.tif]

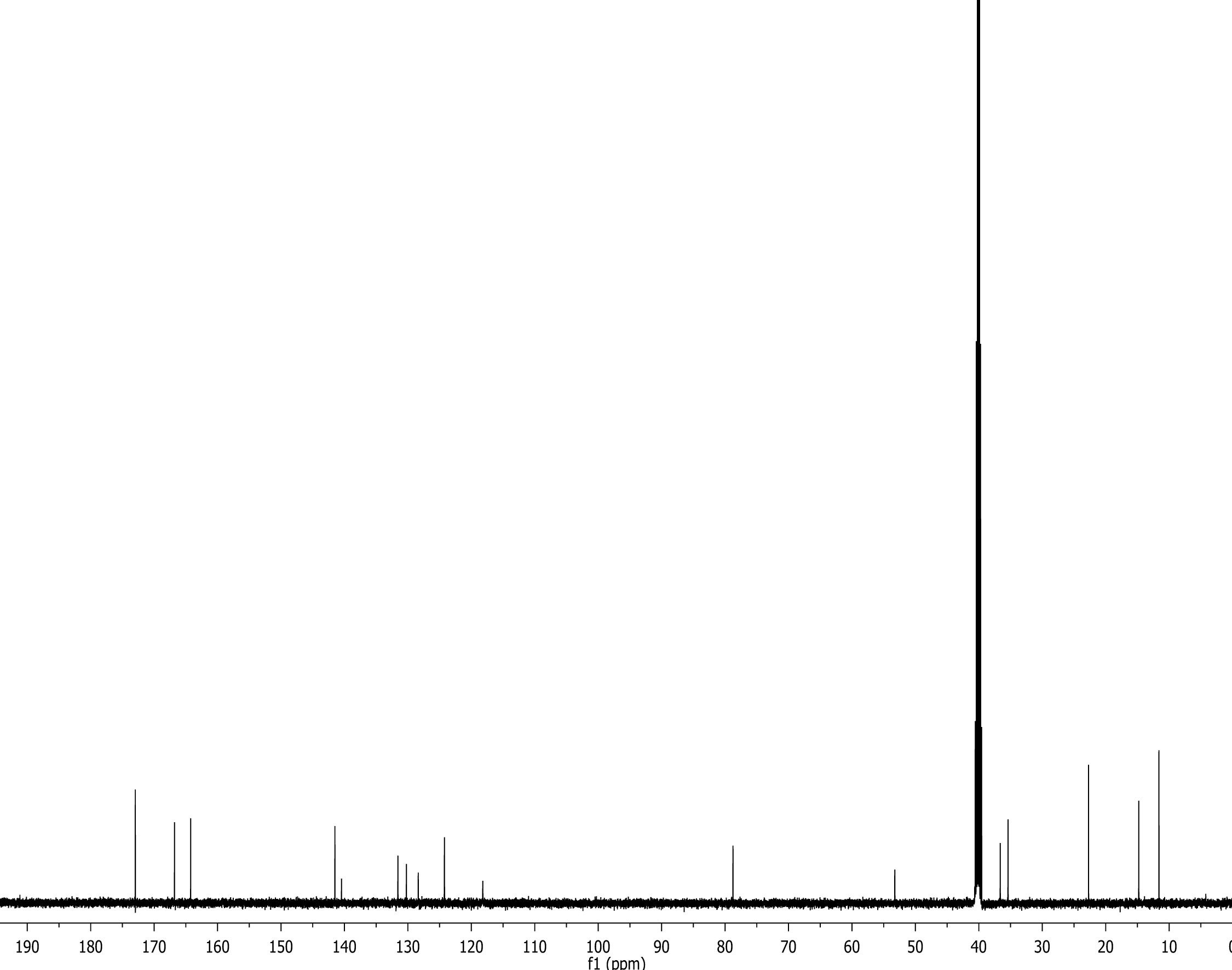

Supplement: S29 Fig — (TIF) [file pone.0166558.s029.tif]

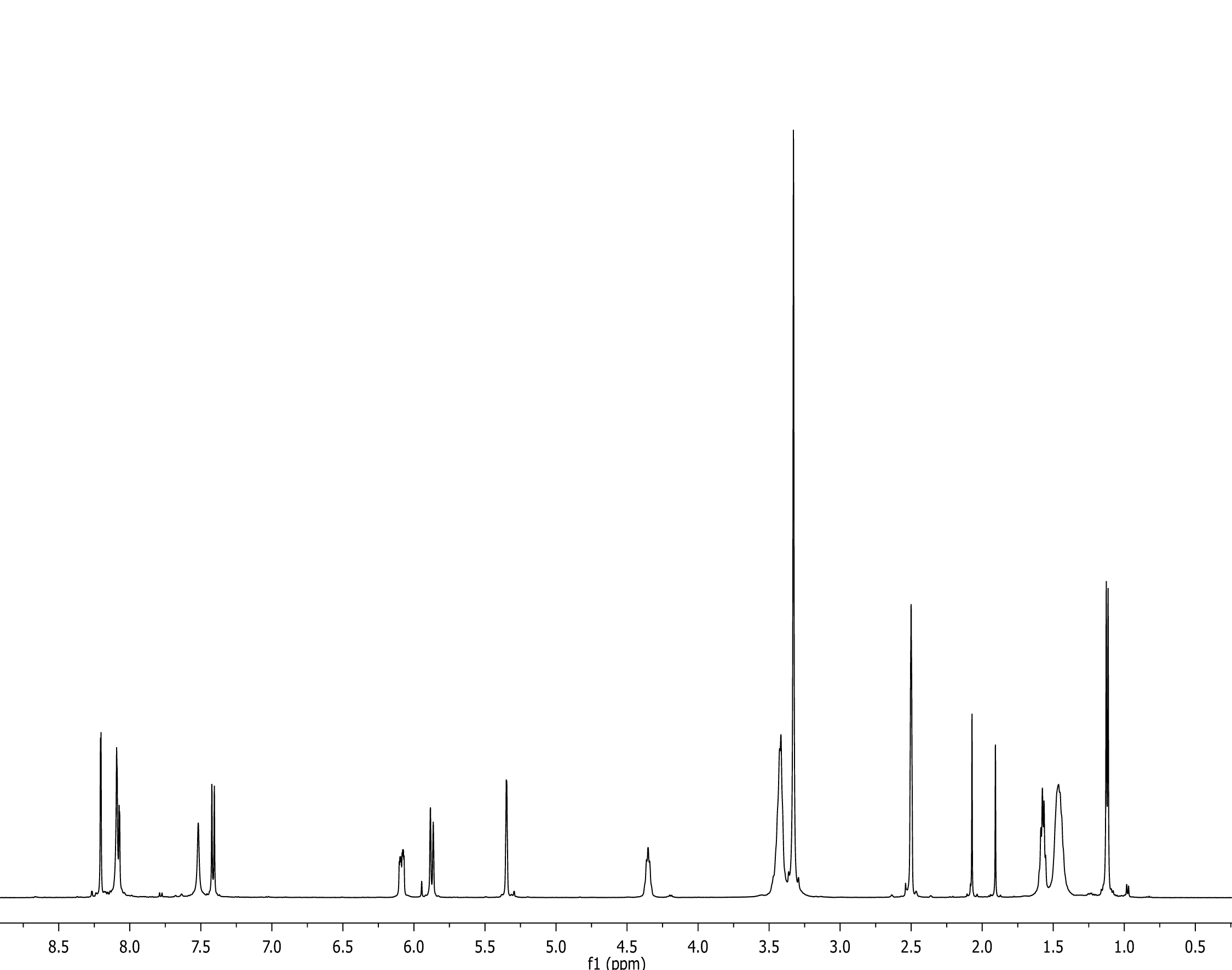

Supplement: S30 Fig — (TIF) [file pone.0166558.s030.tif]

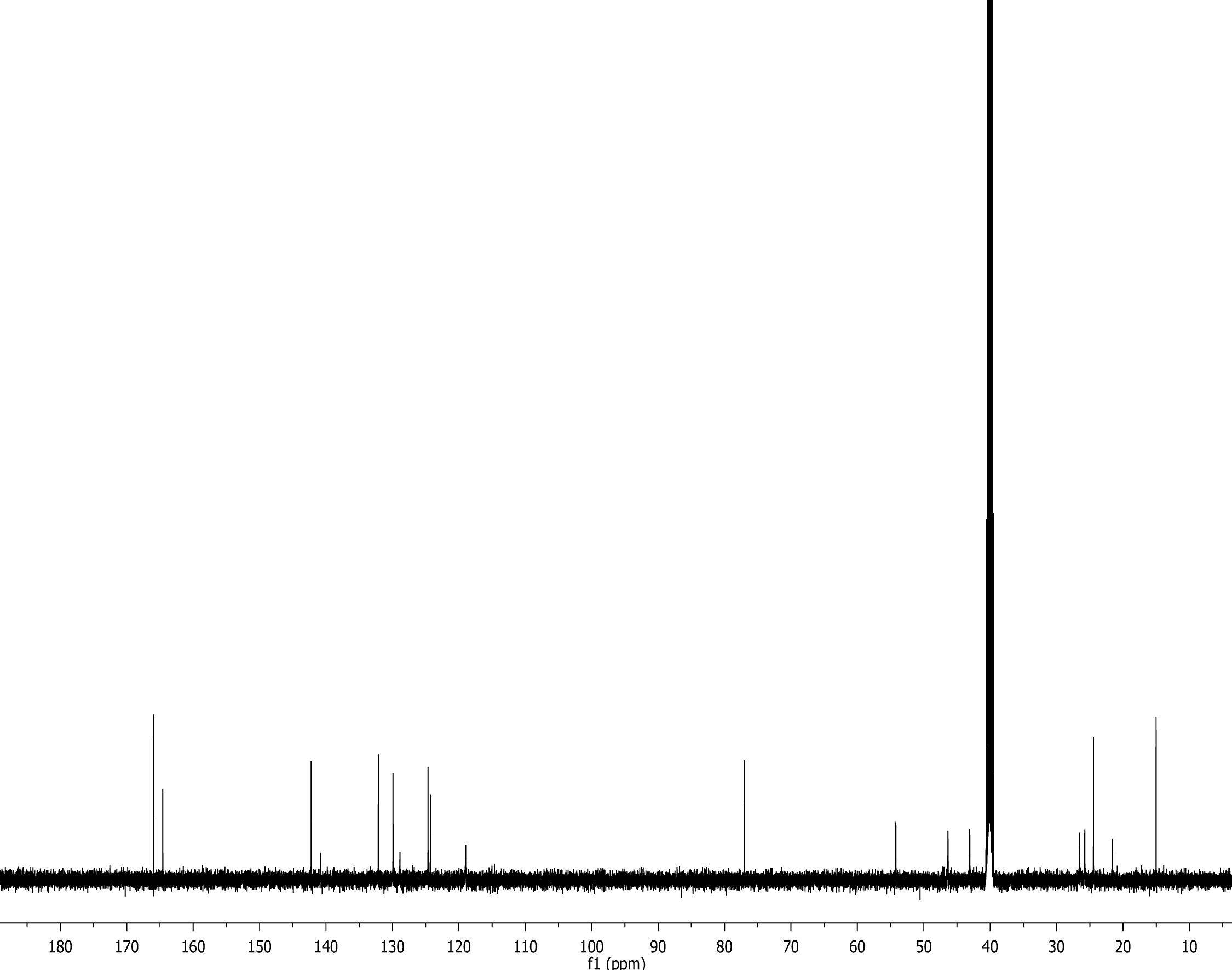

Supplement: S31 Fig — (TIF) [file pone.0166558.s031.tif]

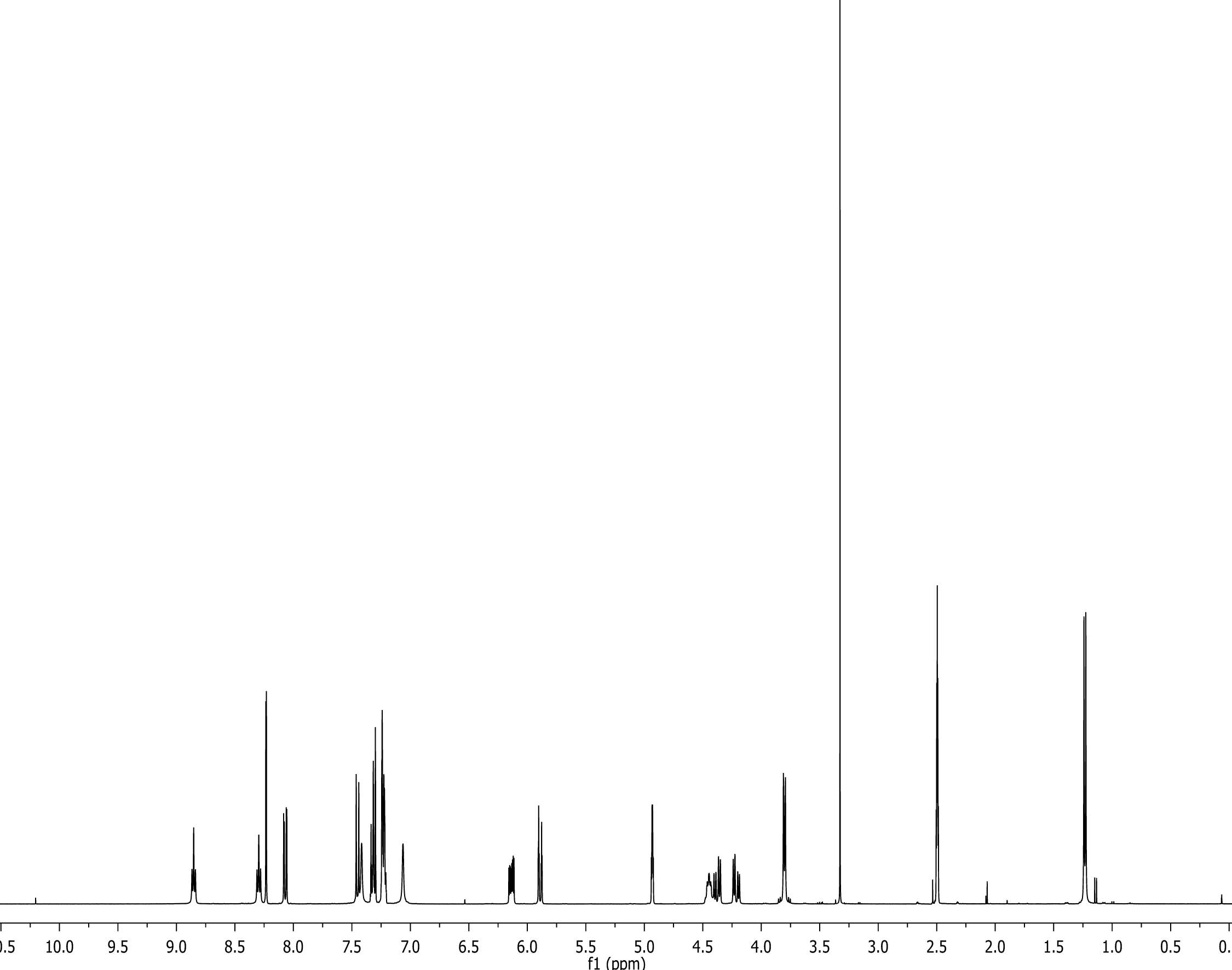

Supplement: S32 Fig — (TIF) [file pone.0166558.s032.tif]

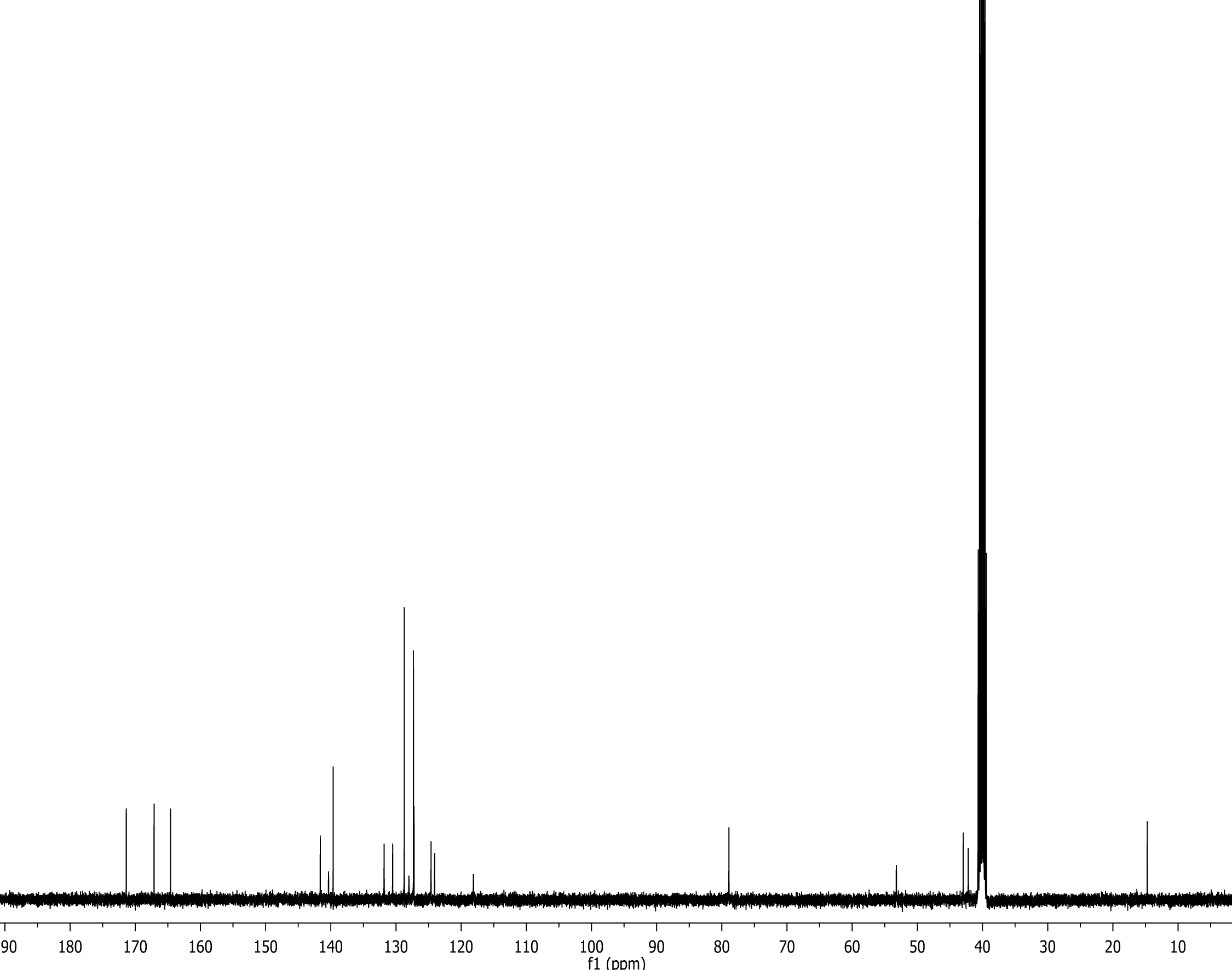

Supplement: S33 Fig — (TIF) [file pone.0166558.s033.tif]

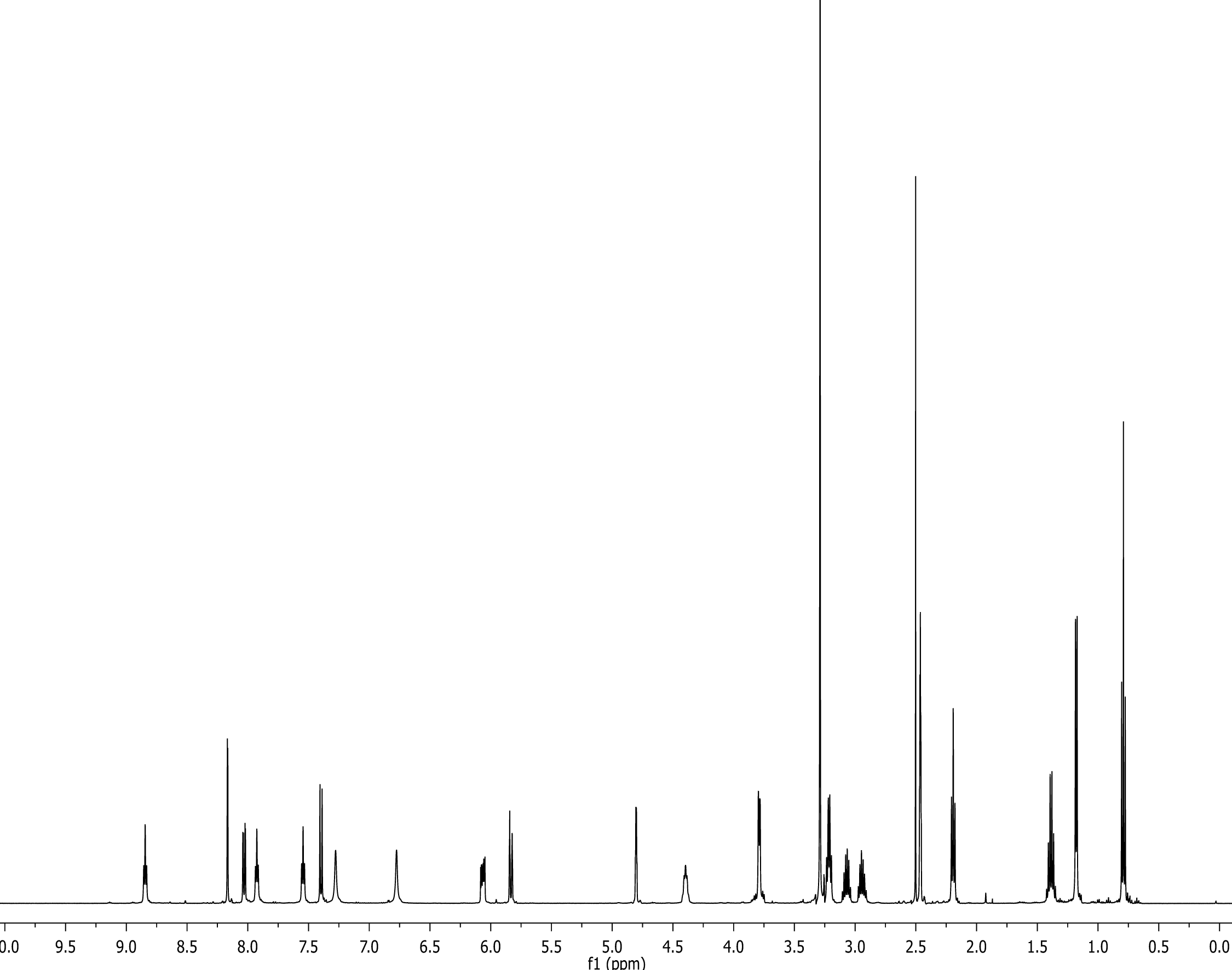

Supplement: S34 Fig — (TIF) [file pone.0166558.s034.tif]

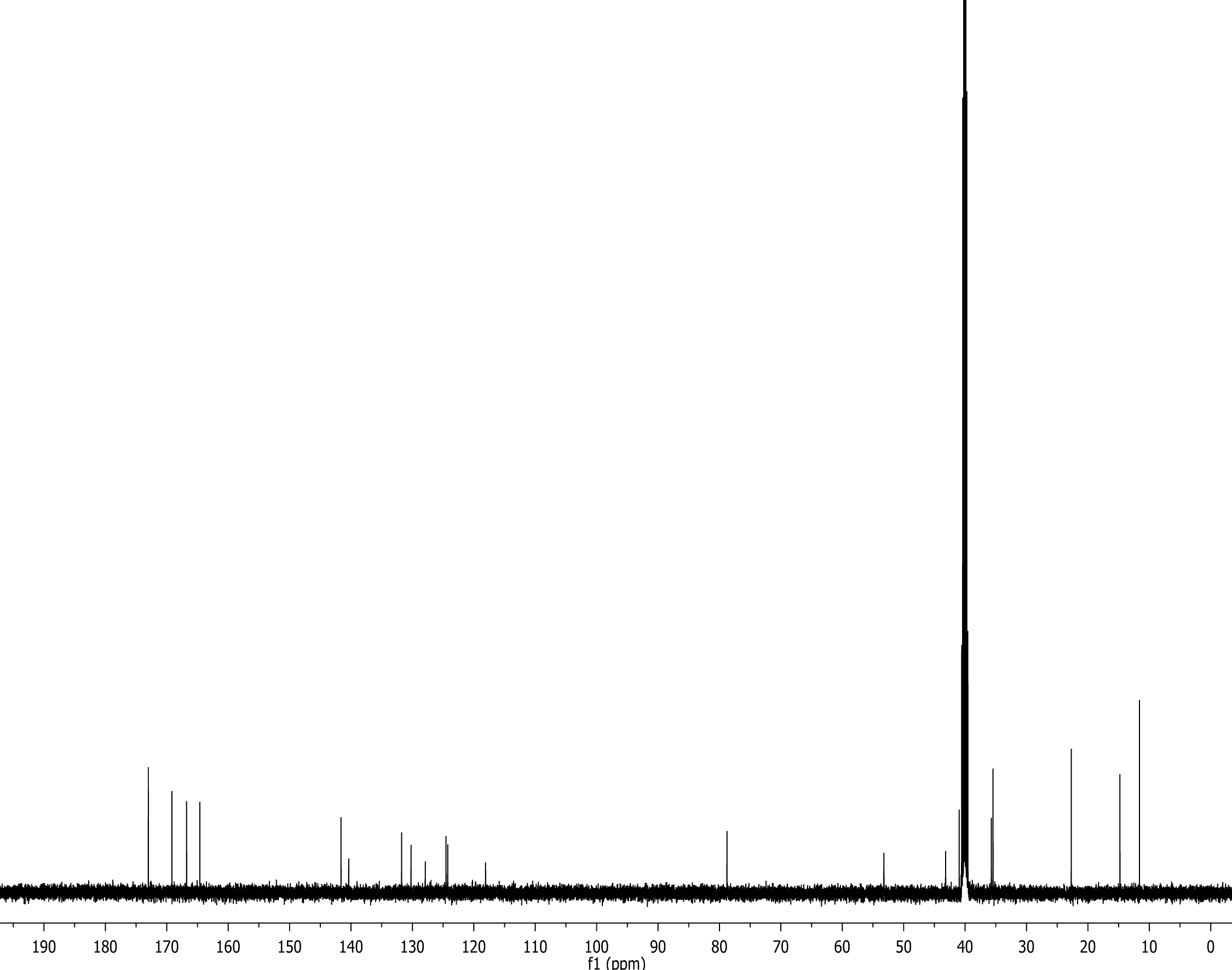

Supplement: S35 Fig — (TIF) [file pone.0166558.s035.tif]

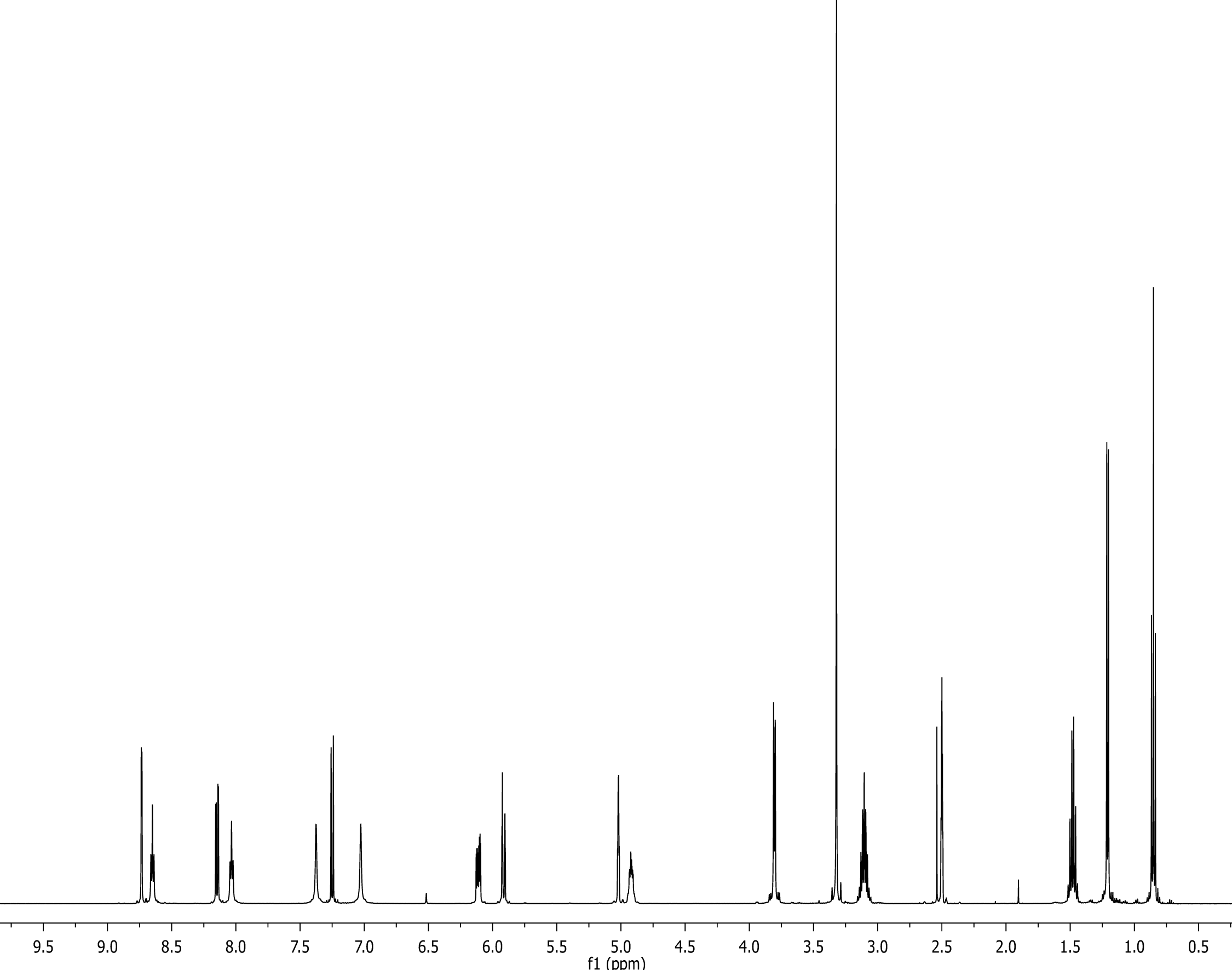

Supplement: S36 Fig — (TIF) [file pone.0166558.s036.tif]

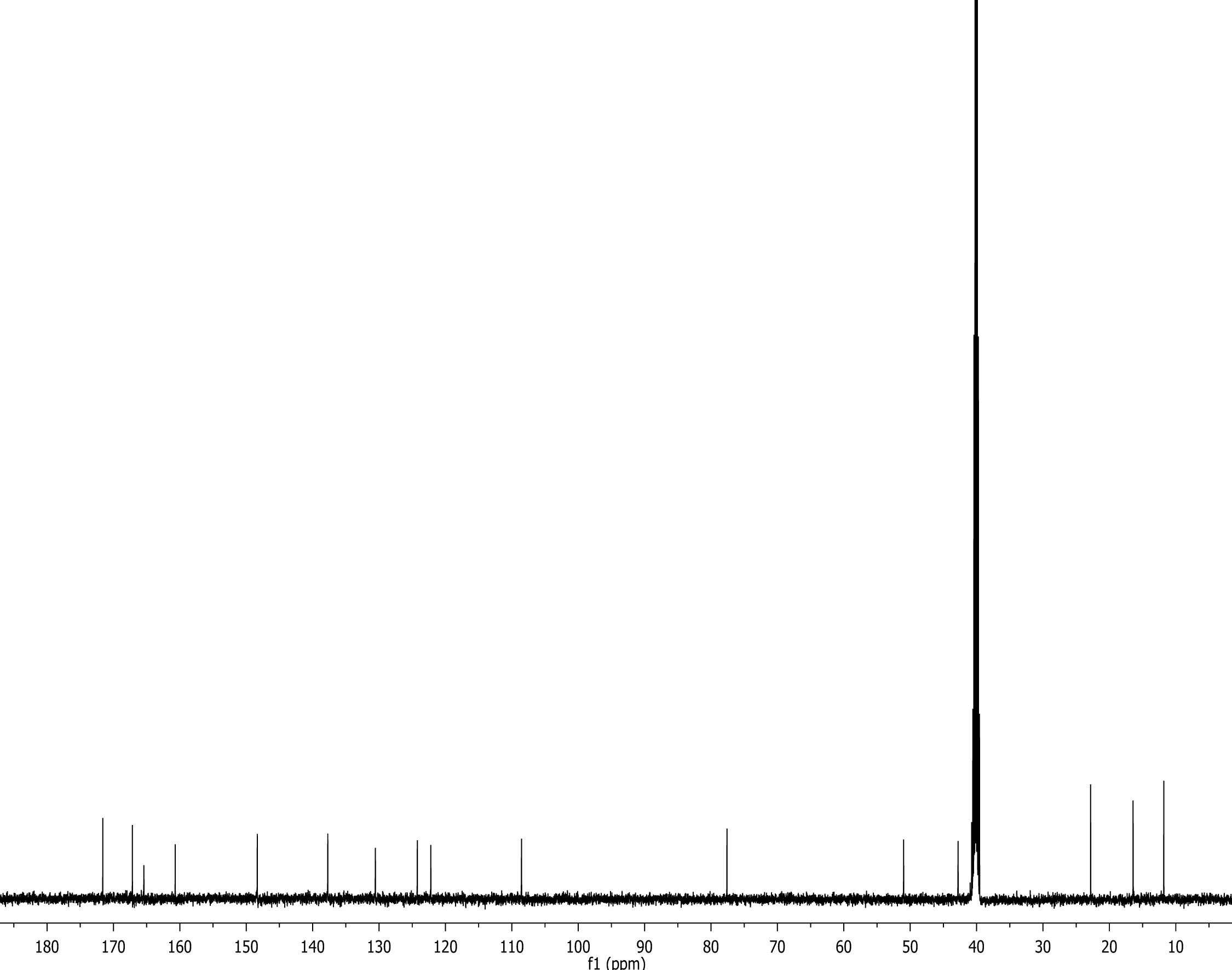

Supplement: S37 Fig — (TIF) [file pone.0166558.s037.tif]

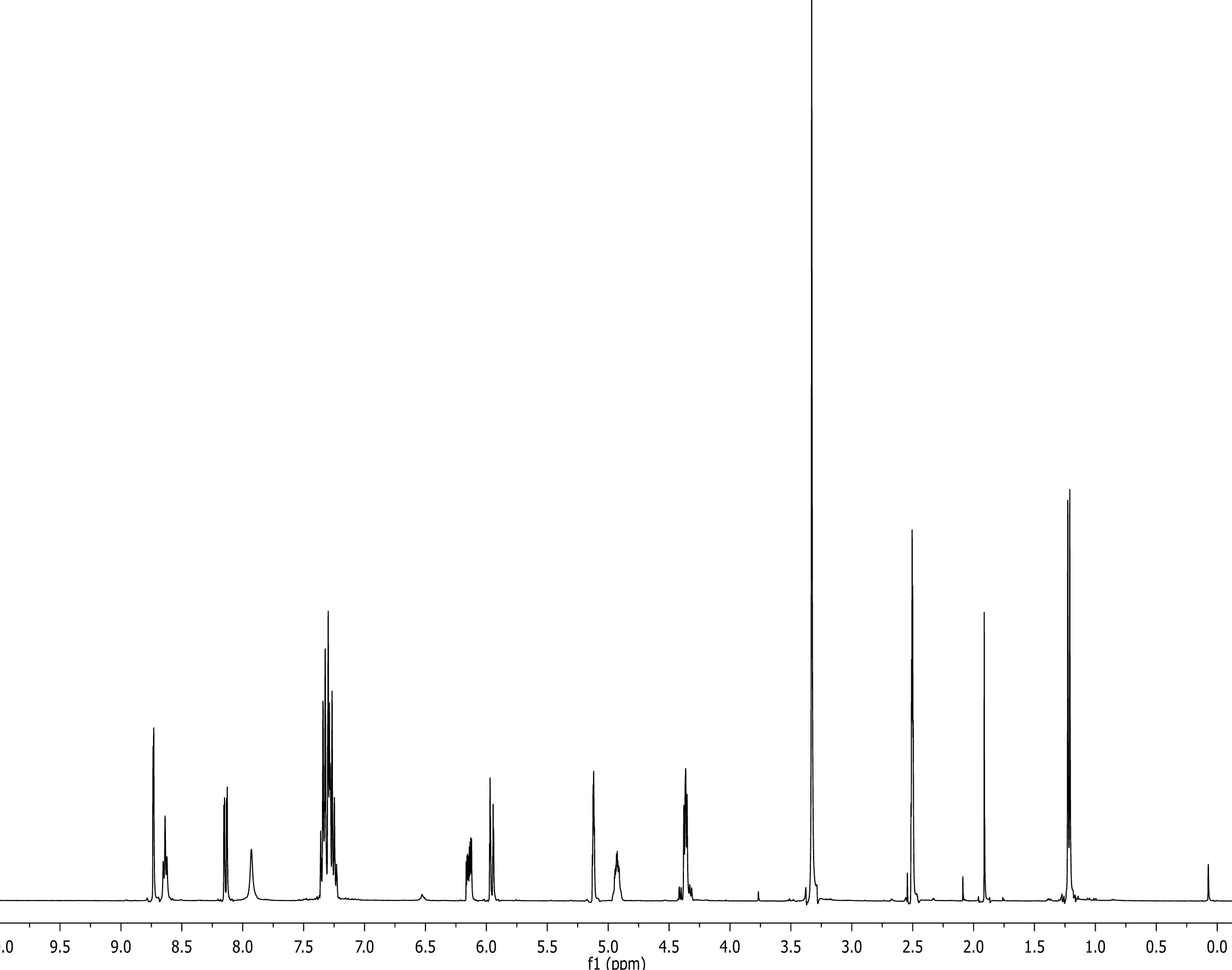

Supplement: S38 Fig — (TIF) [file pone.0166558.s038.tif]

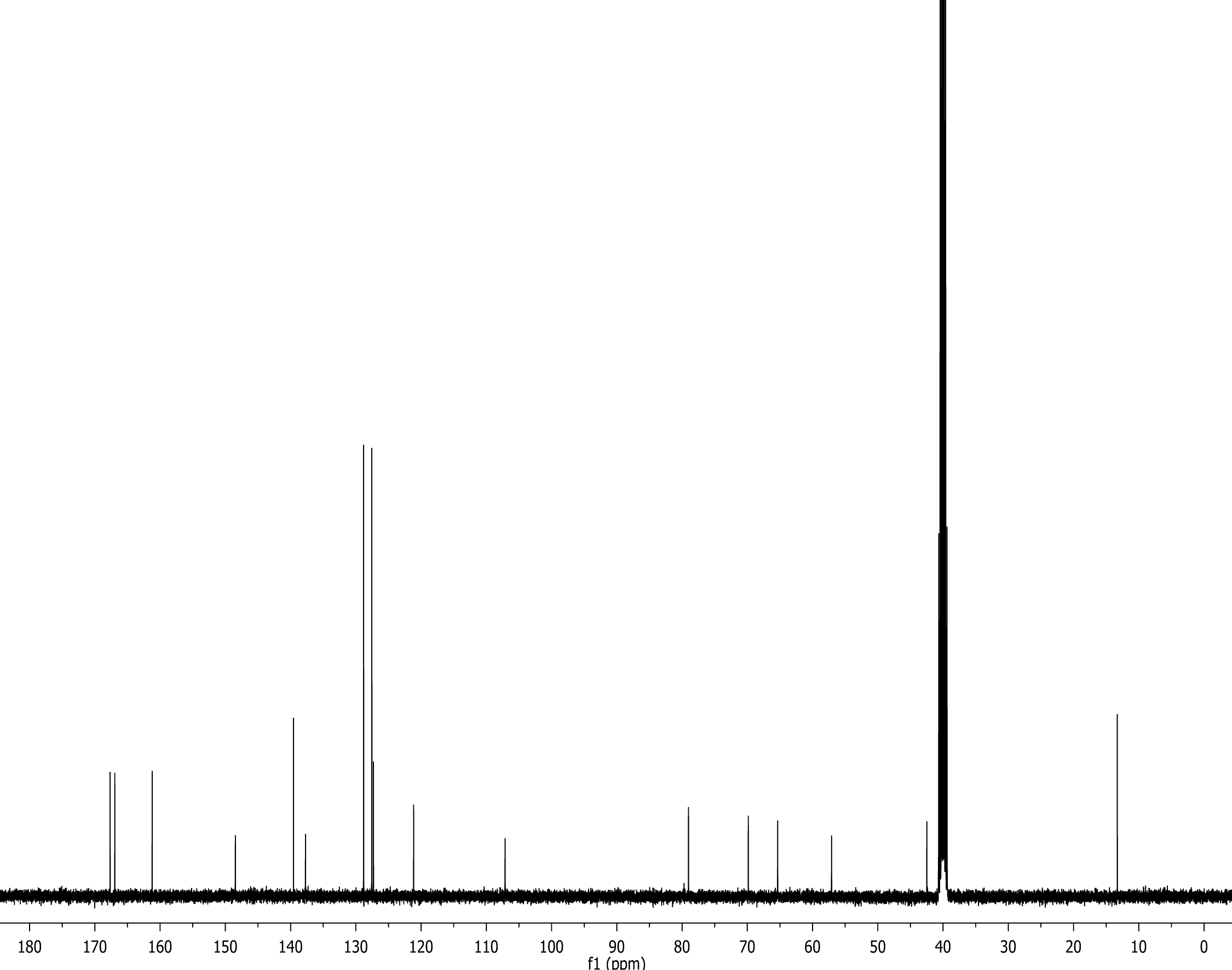

Supplement: S39 Fig — (TIF) [file pone.0166558.s039.tif]

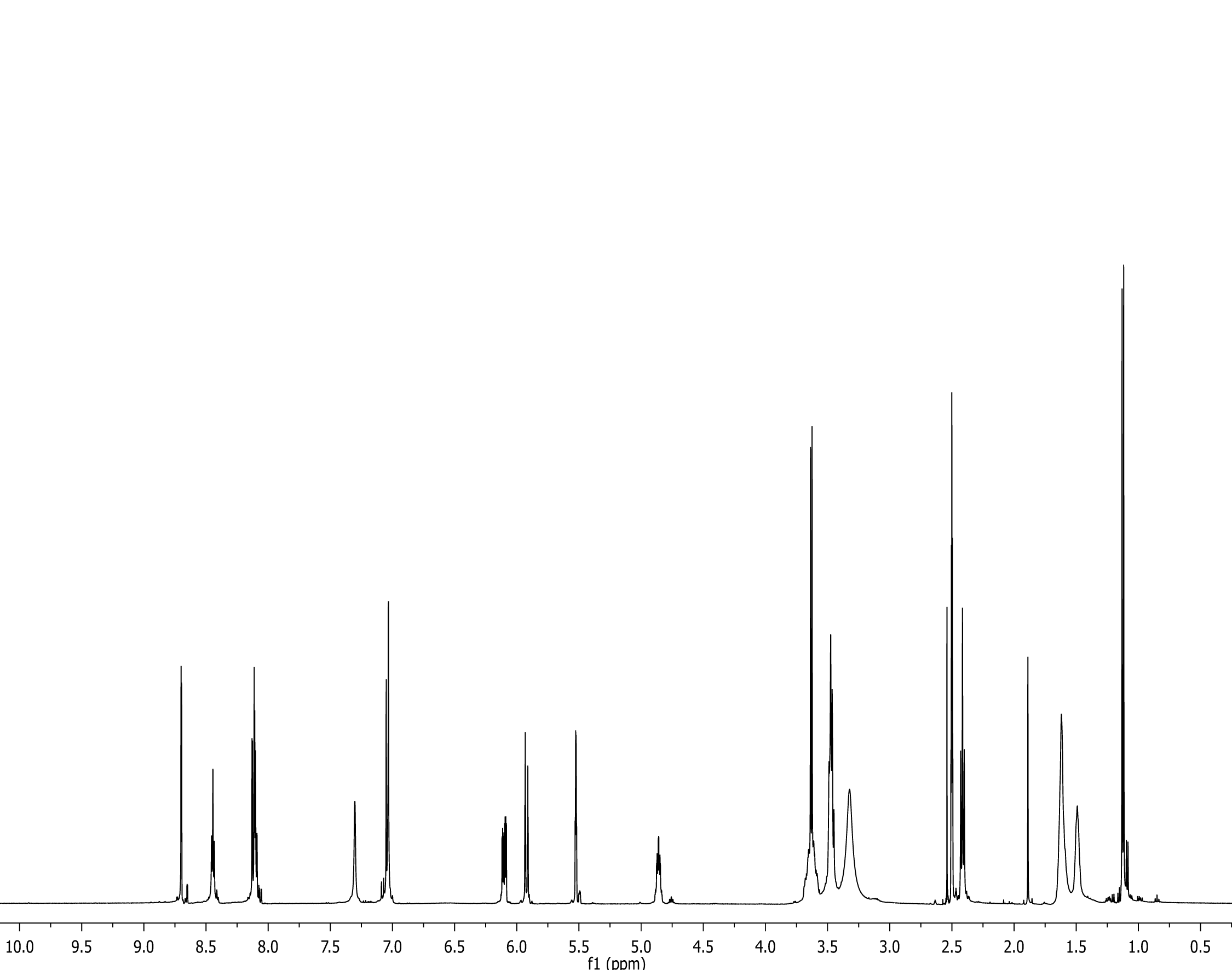

Supplement: S40 Fig — (TIF) [file pone.0166558.s040.tif]

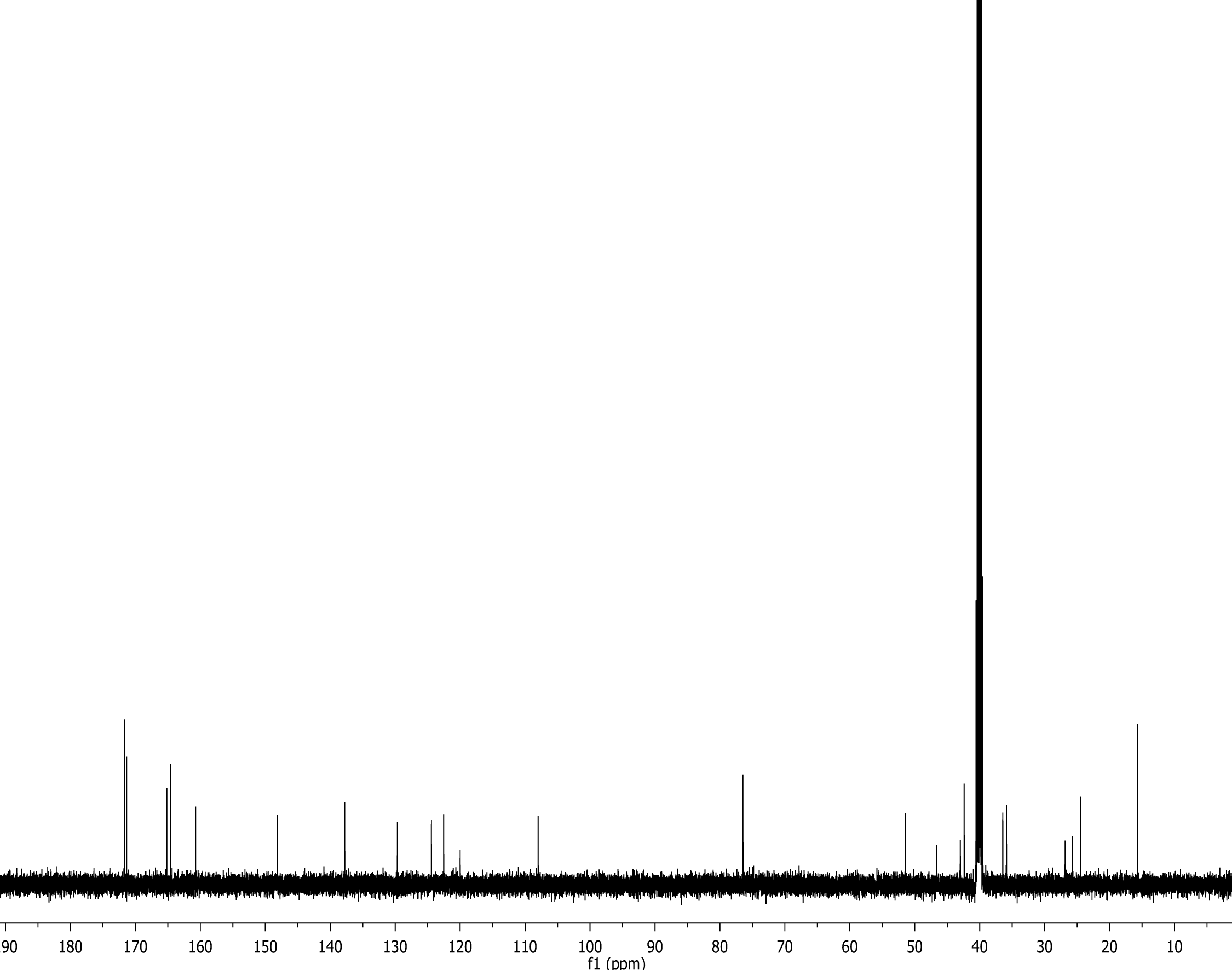

Supplement: S41 Fig — (TIF) [file pone.0166558.s041.tif]

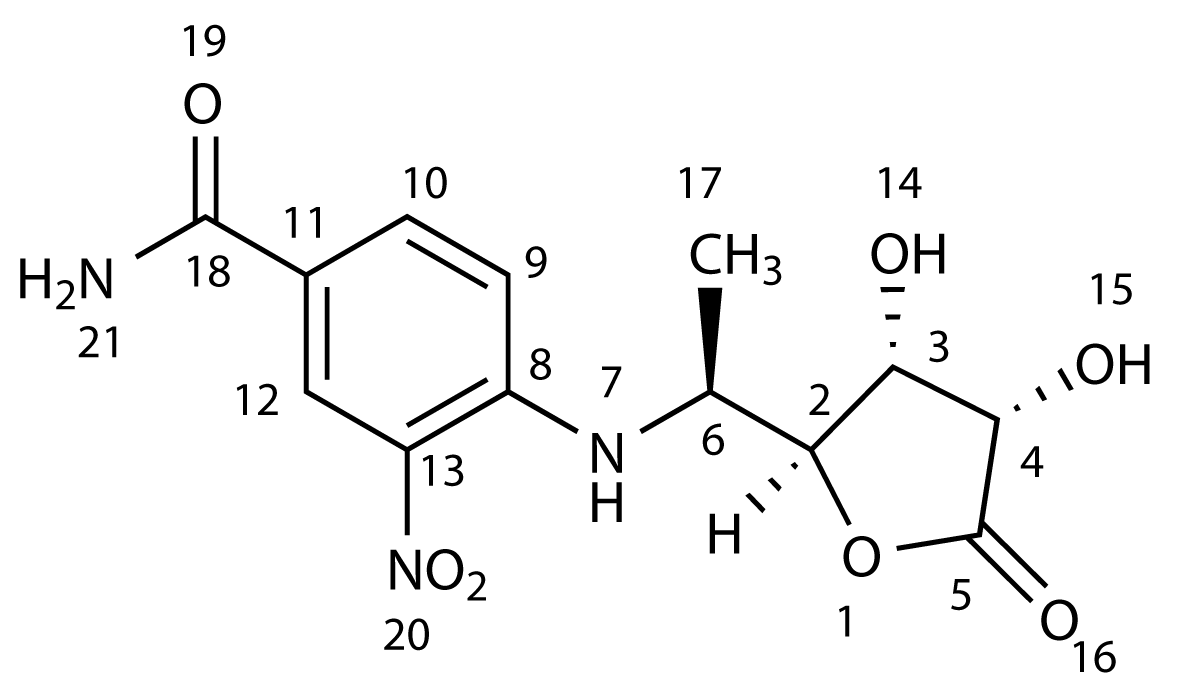

Supplement: S42 Fig — (TIF) [file pone.0166558.s042.tif]

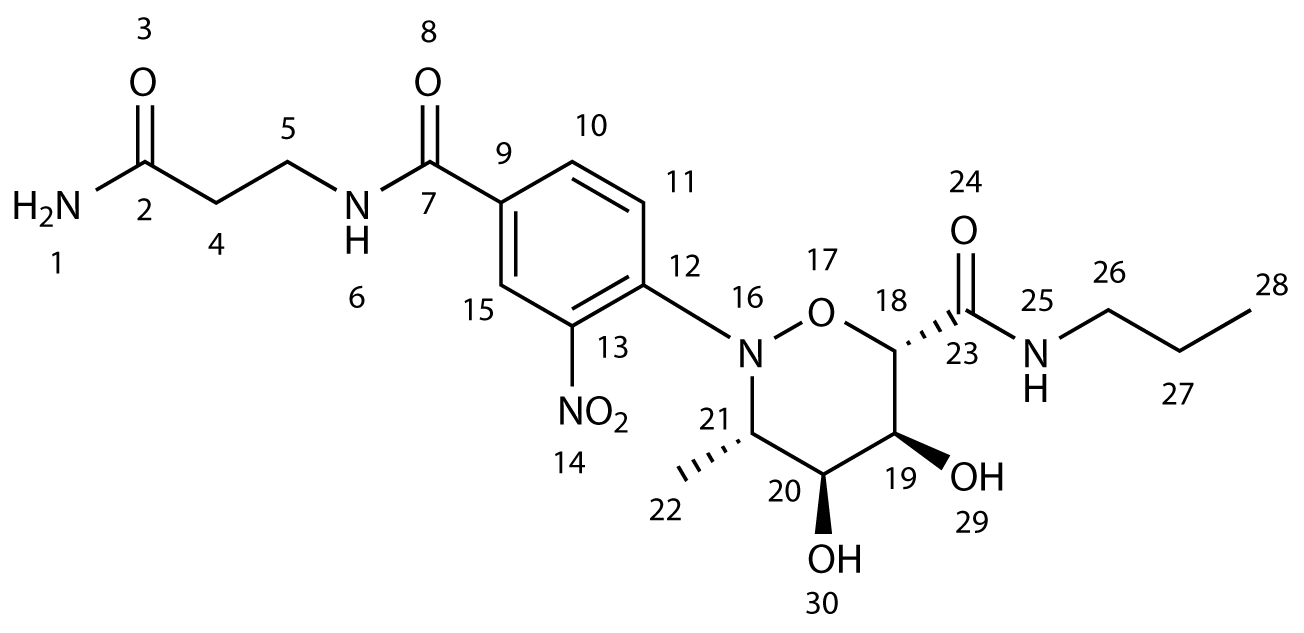

Supplement: S43 Fig — (TIF) [file pone.0166558.s043.tif]

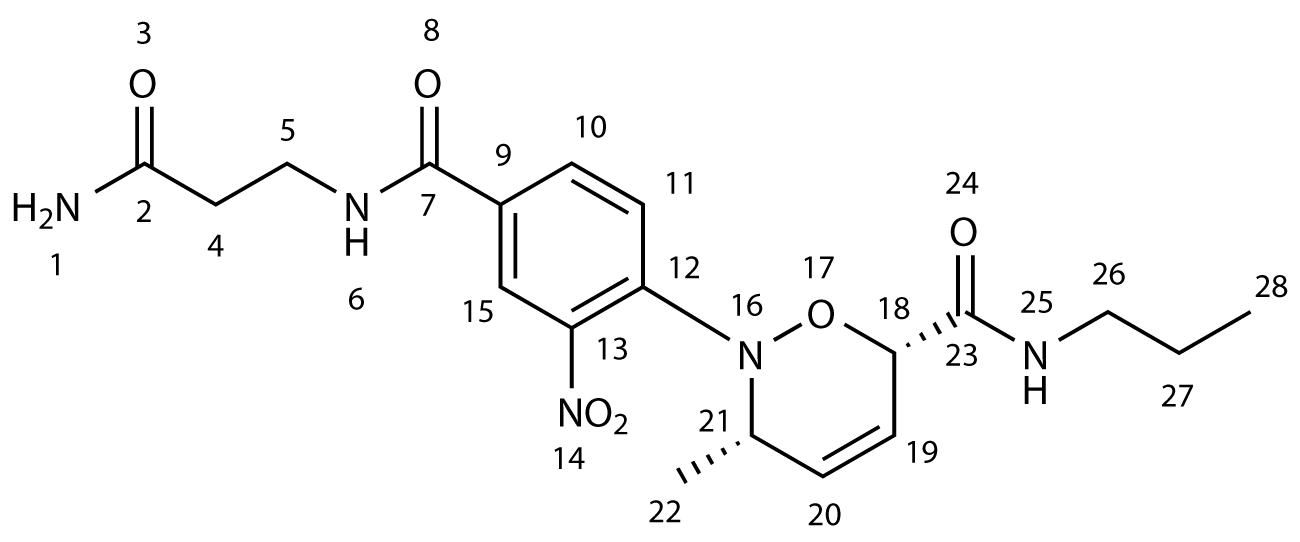

Supplement: S44 Fig — (TIF) [file pone.0166558.s044.tif]
